# Circle‐Seq reveals genomic and disease‐specific hallmarks in urinary cell‐free extrachromosomal circular DNAs

**DOI:** 10.1002/ctm2.817

**Published:** 2022-04-26

**Authors:** Wei Lv, Xiaoguang Pan, Peng Han, Ziyu Wang, Weijia Feng, Xue Xing, Qingqing Wang, Kunli Qu, Yuchen Zeng, Cailin Zhang, Zhe Xu, Yi Li, Tianyu Zheng, Ling Lin, Chengxun Liu, Xuemei Liu, Hanbo Li, Rasmus Amund Henriksen, Lars Bolund, Lin Lin, Xin Jin, Huanming Yang, Xiuqing Zhang, Tailang Yin, Birgitte Regenberg, Fan He, Yonglun Luo

**Affiliations:** ^1^ College of Life Sciences University of Chinese Academy of Science Beijing China; ^2^ IBMC‐BGI Center the Cancer Hospital of the University of Chinese Academy of Sciences (Zhejiang Cancer Hospital) Institute of Basic Medicine and Cancer (IBMC) Chinese Academy of Sciences Hangzhou China; ^3^ Lars Bolund Institute of Regenerative Medicine Qingdao‐Europe Advanced Institute for Life Sciences BGI‐Qingdao Qingdao China; ^4^ Ecology and Evolution, Department of Biology University of Copenhagen Copenhagen Denmark; ^5^ Department of Biochemistry and Molecular Biology, School of Basic Medicine Qingdao University Qingdao Shandong China; ^6^ Department of Nephrology Tongji Hospital Affiliated to Tongji Medical College Huazhong University of Science and Technology Wuhan China; ^7^ Beijing Institutes of Life Science Chinese Academy of Sciences Beijing China; ^8^ College of Life Sciences Tianjin University Tianjin China; ^9^ Section for GeoGenetics GLOBE Institute University of Copenhagen Copenhagen Denmark; ^10^ BGI‐Shenzhen Shenzhen China; ^11^ Department of Biomedicine Aarhus University Aarhus Denmark; ^12^ Steno Diabetes Center Aarhus Aarhus University Hospital Aarhus Denmark; ^13^ Guangdong Provincial Academician Workstation of BGI Synthetic Genomics BGI‐Shenzhen Shenzhen China; ^14^ Department of Clinical Laboratory Renmin Hospital of Wuhan University Wuhan China

**Keywords:** cell‐free DNA, chronic kidney disease, early diagnosis, extrachromosomal circular DNA, miRNA, next generation sequencing, noninvasive biomarkers

## Abstract

**Background:**

Extrachromosomal circular deoxyribonucleic acid (eccDNA) is evolving as a valuable biomarker, while little is known about its presence in urine.

**Methods:**

Here, we report the discovery and analysis of urinary cell‐free eccDNAs (ucf‐eccDNAs) in healthy controls and patients with advanced chronic kidney disease (CKD) by Circle‐Seq.

**Results:**

Millions of unique ucf‐eccDNAs were identified and comprehensively characterised. The ucf‐eccDNAs are GC‐rich. Most ucf‐eccDNAs are less than 1000 bp and are enriched in four pronounced peaks at 207, 358, 553 and 732 bp. Analysis of the genomic distribution of ucf‐eccDNAs shows that eccDNAs are found on all chromosomes but enriched on chromosomes 17, 19 and 20 with a high density of protein‐coding genes, CpG islands, short interspersed transposable elements (SINEs) and simple repeat elements. Analysis of eccDNA junction sequences further suggests that microhomology and palindromic repeats might be involved in eccDNA formation. The ucf‐eccDNAs in CKD patients are significantly higher than those in healthy controls. Moreover, eccDNA with miRNA genes is highly enriched in CKD ucf‐eccDNA.

**Conclusions:**

This work discovers and provides the first deep characterisation of ucf‐eccDNAs and suggests ucf‐eccDNA as a valuable noninnvasive biomarker for urogenital disorder diagnosis and monitoring.

## Graphical abstract

- Urinary cell‐free extrachromosomal circular DNA (ucf‐eccDNA) is common in humans.
- Ucf‐eccDNAs are less than 1000 bp, high in GC content and frequently derived from gene‐rich regions, short interspersed transposable elements (SINEs) and simple repeat elements.
- Patients with advanced chronic kidney diseases (CKDs) have higher ucf‐eccDNA.
- Enrichment of the miRNA gene ucf‐eccDNAs is observed in CKD patients.

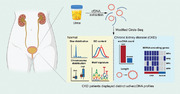

## 1 INTRODUCTION

Over the past decades, considerable interest has been focused on the clinical application of circulating cell‐free deoxyribonucleic acid (cfDNA).^1^, ^2^, ^3^ cfDNA molecules are extracellular DNA fragments originating from illness‐specific cell death and normal cell turnover.^4^ cfDNA can be detected in a variety of human body fluids, such as urine, saliva, cerebrospinal fluid and blood.^5^ When cell apoptosis/death occurs, cfDNA is released into the circulatory system, and a fraction of cfDNA can pass through the glomerular filtration barrier into the urine (also known as transrenal DNA).^6^ Meanwhile, cells lining the urinary tract can also directly secrete cfDNA into urine.^7^ Since urinary cfDNA derives from both the circulatory system and the urinary tract, it can reflect the systemic status of individuals. In addition, previous studies of the fragmentation patterns, copy number aberration, DNA mutation, methylation, integrity and concentrations of urinary cfDNA revealed that urinary cfDNA has emerged as an informative biomarker for prenatal screening, organ transplantation monitoring and infectious/urological malignant disease diagnosis.^3^, ^8^, ^9^, ^10^, ^11^, ^12^, ^13^

Extrachromosomal circular DNA (eccDNA) refers to a group of nonplasmid, circular DNAs that are not part of the nuclear chromosome but originate from and are homologous to chromosomal DNA.^14^ This specific type of DNA molecule reflects genomic plasticity and stability.^15^ eccDNA is commonly present in all cell types and has been found in many organisms, ranging from animals and plants to microorganisms.^16^, ^17^, ^18^, ^19^, ^20^, ^21^ Recent studies have discovered that eccDNA is present in murine and human plasma under both cancerous and healthy conditions.^22^, ^23^, ^24^ Owing to the covalently closed circular structure of eccDNA, plasma eccDNA is considered more resistant to exonucleases and more stable than linear cfDNA. Therefore, plasma eccDNA is expected and has been explored as a novel biomarker in liquid biopsies.^23^, ^24^, ^25^, ^26^

Urine is an attractive clinical source for early disease detection, especially in urogenital disorders.^3^, ^13^ However, the eccDNA profiles in human urine remain unclear, even in healthy individuals. We hypothesised that cell‐free eccDNA also exists in human urine because 1) DNA molecules with molecular weights less than 70 kDa (equal to ∼1.9 kb) have been shown to be freely filtered at the glomerulus and 2) under both pathophysiological and physiological conditions, tissues are known to release eccDNA to the external environment.^23^, ^27^ Thus, we modified the Circle‐Seq method^14^, ^18^ and conducted a systematic characterisation of urinary cell‐free eccDNAs (hereafter depicted as ucf‐eccDNAs) from 28 adult healthy volunteers. Chronic kidney disease (CKD) is an umbrella term for a spectrum of diseases characterised by persistent and nonrecoverable deterioration of renal function and has become a major renal disease globally.^28^ Most CKD patients have already reached an advanced stage with poor prognosis due to a lack of obvious clinical manifestations in the early stages. Therefore, it is critical to discover early diagnostic and reliable therapeutic targets and biomarkers for CKD.^29^ To further explore the potential clinical application of ucf‐eccDNAs, we analysed ucf‐eccDNAs from 21 patients with advanced CKD and found that ucf‐eccDNAs exhibit a distinct disease‐specific profile in CKD patients. To our knowledge, this work, for the first time, systematically characterises the features of cell‐free eccDNA in human urine and reports a framework to leverage ucf‐eccDNAs as potential biomarkers for CKD, which provides comprehensive baseline information for future studies.

## 2 METHODS

### 2.1 Case recruitment and urine sample collections

The primary aims of this study were to explore the presence of cell‐free eccDNA in human urine and to investigate the possibility of ucf‐eccDNAs as biomarkers for clinical application. Twenty‐eight healthy volunteers (mean age 28.9 years [range 22‐39 years]; 19 males/9 females) without any urological system disorders were recruited from the Qingdao‐Europe Advanced Institute for Life Sciences and signed an informed consent form approved by the Institutional Review Board (IRB) of BGI‐Shenzhen (BGI‐IRB) (Table S1). Twenty‐one advanced CKD patients (mean age 50.5 years [range 27‐73 years]; 12 males/9 females) were recruited from Tongji Hospital, affiliated with Tongji Medical College, HUST, and signed another informed consent form approved by the IRB of HUST. CKD stage was assessed based on the estimated glomerular filtration rate (EGFR) (Table S2). The day before urine sample collection, all volunteers were asked to abstain from drinking water for 12 hours. For the healthy control group, approximately 30 ml of the first morn48 hing‐voided midstream urine sample supplemented with .6 ml of 500 mM EDTA was collected between 7 and 8 am on each day of donation. The urine samples were stored at 4°C for further processing. During the sample collection processes, all urine samples from female participants were collected by the participants at their home using a self‐collection urine kit, and urine samples from male participants were collected at our laboratory. However, all samples were delivered to our laboratory within 1 hour at 4°C. For the CKD group, 10 ml of the first morning‐voided midstream urine sample supplemented with .2 ml of 500 mM EDTA was collected between 6 and 7 am on each day of donation. All urine samples were transferred to a nearby laboratory within 3 hours at 4°C.

### 2.2 cfDNA isolation from urine samples

All urine samples from healthy individuals were processed within 2 hours after collection. However, the urine samples from patients with advanced CKD were processed after 3‐4 hours of collection due to some unavoidable circumstances. To obtain the cell‐free portion and remove cellular matter, urine samples were spun 16 000 × g for 10 minutes at 4°C, and the resulting supernatant fluids were passed through a .45‐μm filter. cfDNA was isolated from 10/5 ml (healthy group/CKD group) cell‐free urine supernatant using a MGIEasy Circulating DNA Extraction Kit (MGI‐BGI, China) and eluted in 55 μl RNase‐free water. The cfDNA concentration was measured by a Qubit dsDNA HS Assay kit (Invitrogen).

### 2.3 Plasmids

The pAAV‐saCas9‐Backb (P895; 7447 bp) and BE4‐Gam (P1035; 9444 bp) plasmids were purified from *Escherichia coli* using the TIANGEN mini plasmid purification kit.

### 2.4 Cell‐free eccDNA purification and amplification

To remove the linear portions of urinary cfDNA, 40 μl of urinary cfDNA with added spike‐in plasmid control (∼ 100 copies of P895 and ∼ 100 copies of P1035) was treated with 25 units of Plasmid‐Safe Dnase (Epicenter) at 37°C for 24 hours. The Plasmid‐Safe Dnase was then inactivated by incubating samples at 70°C for 30 minutes. The digestion products were cleaned up from the reaction mixes using 2.0 × WAHTS® DNA clean beads (Vazyme) and eluted in 30 μl RNase‐free water. Approximately 50% (14 μl) of the total volume of resultant circular DNA was subjected to a RCA reaction with incubation at 30°C for 16 hours. The RCA reaction system involved 1 μl Phi29 polymerase (Thermo), 4 μl 10 × Phi29 buffer (Thermo), 2 μl exonuclease‐resistant random primer (Thermo), 4 μl 2.5 μM dNTP mixture (Takara), .8 μl 100 mM DTT and 14.2 μl RNase‐free water. The phi29‐amplified products were recovered by 2.0 × WAHTS® DNA clean beads, eluted in 80 μl RNase‐free water and quantified by Qubit 3.0.

### 2.5 Quality control by agarose gel electrophoresis

Successful amplification was assessed by agarose gel electrophoresis (.7%). Moreover, 1 μl of RCA products was double‐digested with MssI and NotI restriction enzymes (Thermo) for 30 minutes at 37°C. The digestion products were also subjected to agarose gel electrophoresis (.7%).

### 2.6 Library preparation and sequencing

Approximately 500 ng of amplified DNA was sheared by sonication (Covaris LE220) to generate a median size of 400 bp. Then, 50 ng of fragmented DNA was input for library construction using the MGIEasy DNA Library Preparation Kit (MGI‐BGI, China). The length distribution and quality of each library were examined by a Bioanalyzer 2100 (Agilent). DNA libraries were sequenced (PE150) on the MGISeq‐2000 platform (BGI‐Qingdao, Qingdao, China).

### 2.7 eccDNA identification from Circle‐Seq data

To detect eccDNA from PE150 high‐throughput sequencing data, we applied Circle‐Map (v1.1.4) software (https://github.com/iprada/Circle‐Map).^30^ Sequencing reads were first aligned to the human reference genome (hg38 genome download from UCSC) using BWA‐MEM. Then, two BAM files were sorted according to read names and coordinates for the extraction of circular reads. The above files were used for detecting the coordinates of each eccDNA.

To improve the accuracy of eccDNA detection, several filtering steps were conducted. The specific settings were as follows: (1) split reads ≥ 2, (2) circle score ≥ 20, (3) coverage increase in the start coordinate ≥ .33, (4) coverage increase in the end coordinate ≥ .33, (5) coverage continuity ≤ .1 and (6) The SD of coverage smaller than the mean coverage over the whole eccDNA region. The detailed information of each parameter is described in Iñigo et al.^30^

### 2.8 Genomic feature acquisition

The genomic features, such as the length of each chromosome, the number of Alu‐ elements and coding genes in each chromosome, and the position of each gene and miRNA‐coding elements on chromosomes, were retrieved from Ensembl database assembly GRCh38.

### 2.9 Genomic annotation of ucf‐eccDNAs

The genomic annotation data were downloaded from the UCSC table browser (https://genome.ucsc.edu/cgi‐bin/hgTables/). Seven major classes of genomic elements (3′UTR, 5′UTR, CpG island, exon, Gene2KbD, Gene2KbU and intron) were used to map ucf‐eccDNAs with junction sites. We used the “observed/expected ratio of genomic elements” for statistics, and the “observed/expected ratio of genomic elements” was calculated according to the following formula:

$$\begin{array}{ccc} & & observed/\;expected\;ratio\;of\;genomic\;elements\\ & & \;=\frac{Percentage\;of\;unique\;eccDNA\;falling\;in\;a\;certain\;type\;of\;element}{\;Percentage\;of\;the\;length\;of\;that\;element\;over\;the\;length\;of\;whole\;genome}\% \end{array}$$

Repetitive sequence analysis was performed as previously reported.^19^ Briefly, using BedTools multicov and BedTools groupby, we counted the number of reads mapped to the specific repetitive element (RepeatMasker open‐4.0.5; http://repeatmasker.org). The normalised mapping ratio was calculated as the percent of reads mapped to the specific repetitive element divided by the percent of the specific repetitive element in the nuclear genome.

### 2.10 Generation of random eccDNA datasets

Based on the weighted average of chromosome length, we generated 28 random data sets of *in silico* eccDNAs across the genome. The number of *in silico* eccDNAs in each data set was in one‐to‐one correspondence with the number of ucf‐eccDNAs in each urine sample. To better emulate our detected ucf‐eccDNAs, the size of *in silico* eccDNAs ranged randomly between 150 and 850 bp.

### 2.11 Characterisation of the ucf‐eccDNA fragment junction site

Bedtools 2.29.2 was used to extract 10 bp up‐ and downstream DNA sequences of each eccDNA junction site. HomerTools was used to calculate the mean per base mononucleotide frequencies. The motif signature of the eccDNA fragment junction site was visualised by the R package ggseqlogo‐0.1.

### 2.12 Validation of potential biomarkers

miRNA‐related eccDNA validation was performed by outward polymerase chain reaction (PCR) and Sanger sequencing, and the PCR primers are listed in Table S3. Each 30 μl PCR system included 50 μg phi29‐amplified DNA products, 500 nM primer and 15 μl NEBNext High‐Fidelity 2X PCR Master Mix (NEB) and PCR for 32 cycles. All reactions were performed with a nontemplate control (NTC). The PCR products were tested by agarose (2.5%) gel electrophoresis, and the target products were recovered by a QIAEX II Gel Extraction Kit. Sanger sequencing of target products confirmed the junction site of all selected miRNA‐related circles.

### 2.13 Statistical analysis

All statistical tests were implemented by R‐4.1.1. The difference comparison of two groups was performed by Wilcoxon test. A Pearson's correlation test was used for correlation analysis. *p* < .05 indicated statistical significance.

## 3 RESULTS

### 3.1 A modified method for purifying ucf‐eccDNAs

**FIGURE 1:**
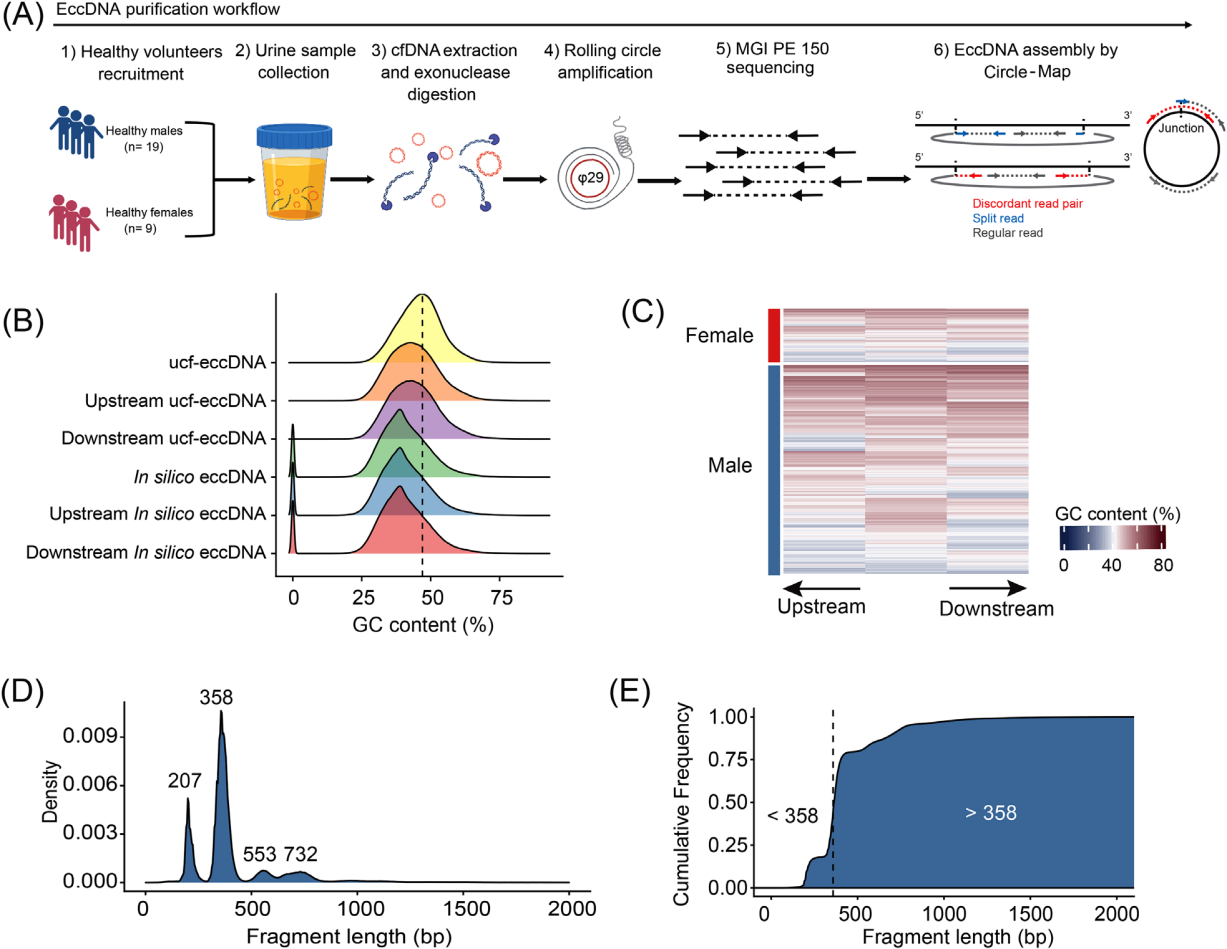
Modified Circle‐Seq method for mapping ucf‐eccDNAs. (A) Workflow of cell‐free eccDNA purification and identification from urine samples. Total cell‐free DNAs consisting of both circular and linear DNAs were isolated from the urine samples. To purify circular DNAs, linear DNA molecules were removed by exonuclease digestion. The enriched circular DNAs were then amplified by rolling circle amplification (RCA). The RCA‐amplified products were finally subjected to library construction and sequencing. Circle‐Map software was used to detect eccDNAs from sequencing data based on plit read and discordant read pairs. (B‐E) GC content and length distributions of ucf‐eccDNAs from healthy individuals (pooled data from 28 cases). (B) GC content distribution of ucf‐eccDNAs, *in silico* eccDNAs and their downstream and upstream regions of equivalent length. (C) Heatmap showing the GC content of 4000 randomly selected ucf‐eccDNAs and their downstream and upstream regions of equivalent length. (D) Length distribution of ucf‐eccDNAs. (E) Cumulative frequency plot of ucf‐eccDNAs. Ucf‐eccDNAs: urinary cell‐free extrachromosomal circular deoxyribonucleic acid

To explore the presence of cell‐free eccDNA in human urine, we modified the Circle‐Seq method, which was developed previously for enriching eccDNAs from yeast and human somatic tissues (see methods).^14^, ^18^ Briefly, we isolated total cfDNA from urine samples using a magnetic bead‐based DNA purification approach. Then, as a positive control of circular DNA, plasmids were spiked into the total urinary cfDNA before exonuclease digestion. Linear DNAs were then enzymatically removed from the total cfDNA by plasmid‐safe adenosine triphosphate (ATP)‐dependent exonuclease treatment. The enriched circular DNAs were subjected to rolling circle amplification (RCA) using a highly processive phi29 DNA polymerase. *MssI* and *NotI* restrict enzyme digestion, which cleaves the spike‐in plasmids, showed that the spike‐in plasmid control was amplified by RCA (Figure S1). In addition to the spike‐in plasmids, a strong DNA staining signal was also detected for urine samples but not the plasmid‐only control, indicating that eccDNAs are present in the urine samples and were captured by the modified Circle‐Seq protocol (Figure S1). Based on the optimised protocol of eccDNA purification from urine samples, as depicted in Figure 1A, we first isolated ucf‐eccDNAs from 28 adult healthy volunteers (mean age 28.9 years [range 22‐39 years]; 19 males/9 females). The RCA‐amplified ucf‐eccDNAs were subjected to deep sequencing with an MGI 150‐bp pair end (PE) read sequencing strategy. Circle‐Map software, a highly sensitive circular DNA detection method,^30^ was used to identify ucf‐eccDNAs from sequencing data based on split read and discordant read pairs (ref. genome hg38) (Figure 1A).

### 3.2 Genome‐scale map of ucf‐eccDNAs

**FIGURE 2:**
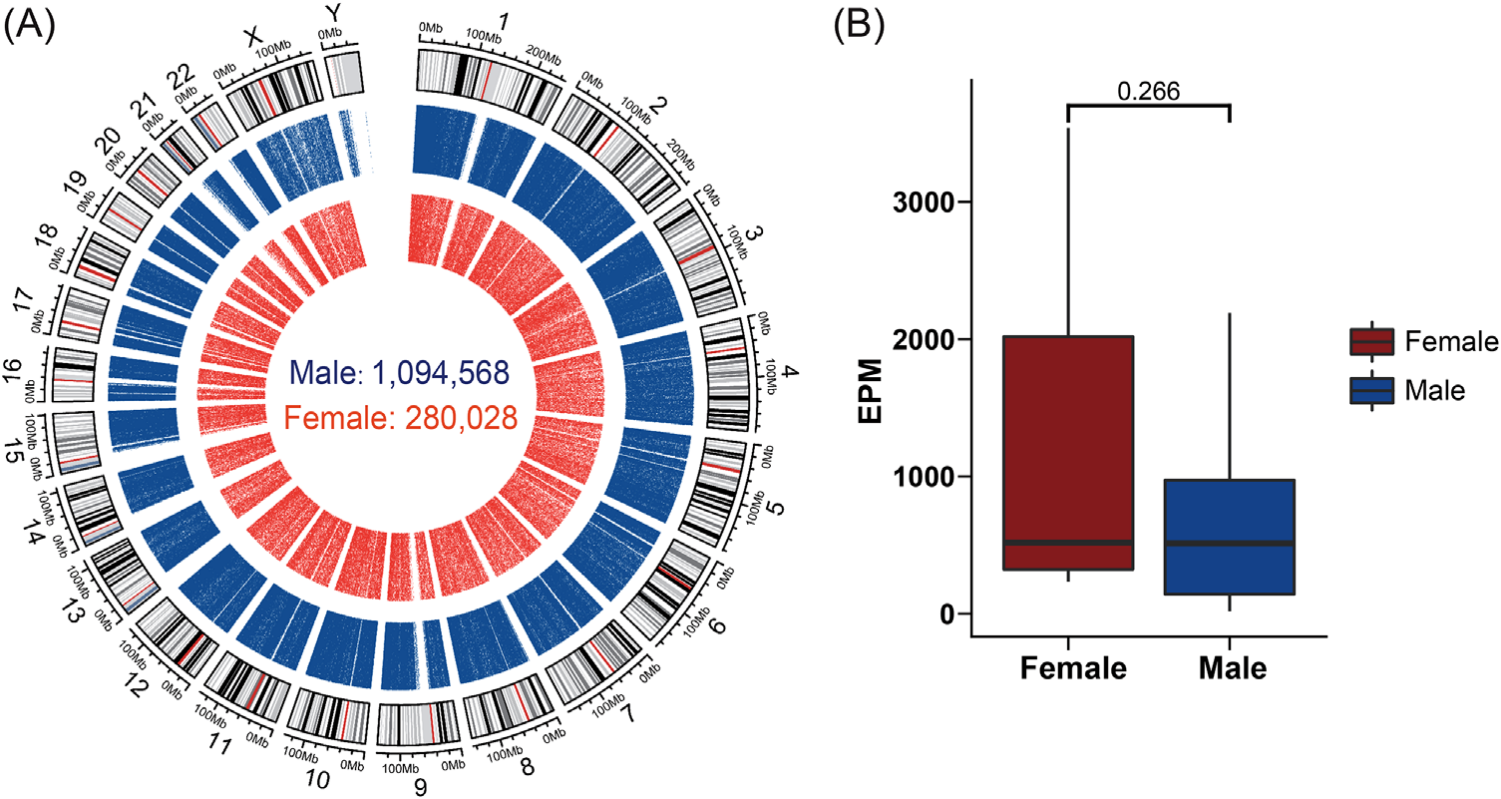
Genome‐scale map of ucf‐eccDNAs. (A) Circos plot presenting the genome‐wide distribution of ucf‐eccDNAs. From inside to outside, the red and blue dots represent female‐derived ucf‐eccDNAs (pooled data from nine cases) and male‐derived ucf‐eccDNAs (pooled data from 19 cases), respectively. The count of detected ucf‐eccDNAs in males and females is marked in the middle of the Circos plot. (B) Comparison of the number of ucf‐eccDNA per million mapped reads (EMP) between all of the healthy males and females (*p* value = .266). Ucf‐eccDNAs: urinary cell‐free extrachromosomal circular deoxyribonucleic acid

In total, we detected over one million (*N* = 1,374,596) unique ucf‐eccDNAs from all urine samples, and the number of eccDNAs varied appreciably among each urine sample (male, average: 57,609, range 2991‐198,120; female, average: 31,114, range 8322–81,691) (Table S1). Since the number of detected eccDNAs is affected by the sequencing depth, we normalized it by calculating the numbers of eccDNAs per million mapped reads (EPM). There was no significant difference in the normalized number of eccDNAs between males and females (male: mean, 679; 95% CI, 378–980 vs. female: mean, 1244; 95% CI, 190–2298) (*p* value = .266, Wilcoxon test) (Figure 2B). Thus, male and female ucf‐eccDNAs were merged for most downstream analyses unless specified. Most (64.4%) ucf‐eccDNAs were mapped to intragenic regions, and 49.7% of ucf‐eccDNAs were mapped to protein coding genes. The number of eccDNAs also varies greatly between different protein coding genes. Further analysis showed that there was a significantly positive correlation between the length of protein coding genes and the number of eccDNAs (Pearson's R = .88) (Figure S2). Because intragenic regions and protein‐coding genes are better annotated than other DNA regions, to ensure that this was not caused by mapping and annotation issues, we generated 28 random data sets with ∼1.2 million *in silico* eccDNAs (size from 150 to 850 bp). Compared to ucf‐eccDNAs, the randomly generated *in silico* eccDNAs were less mapped to intragenic regions (64.4% vs. 57% of ucf‐eccDNA vs. *in silico* eccDNA) and protein‐coding genes (41.9% vs. 49.7% of ucf‐eccDNA vs. *in silico* eccDNA), suggesting that ucf‐eccDNAs are more frequently derived from intragenetic regions. Since ucf‐eccDNAs have not been previously reported, we focused on characterising the genomic and sequence features of ucf‐eccDNAs and sought to investigate the potential clinical application of ucf‐eccDNA, thus providing a valuable reference resource for future investigations.

### 3.3 Guanine‐cytosine (GC) content and fragment length distributions of ucf‐eccDNAs

We first analysed the GC content of ucf‐eccDNAs, as it is well known that GC content is associated with DNA stability, structure, evolution and functions.^31^ The overall GC content of the human genome is approximately 45%, and previous studies have reported that eccDNA molecules more frequently originate from genomic regions with high GC content.^21^, ^32^ The GC content of ucf‐eccDNAs (peak value, 46.8%) was significantly higher than that of their downstream (peak value, 43.1%) and upstream regions (peak value, 42.8%) of equivalent length, while this high GC preference was not seen in the randomly generated in silico eccDNAs (peak value, 39%) (Figure 1B,C).

We next analysed the size distribution of ucf‐eccDNAs. Most ucf‐eccDNAs (99%) were smaller than 1000 bp. Interestingly, ucf‐eccDNAs are enriched in four characteristic peaks positioned at 207, 358, 553 and 732 bp with a periodicity of ∼160‐200 bp. Among them, the 358‐bp peak was the most pronounced (tenfold larger than the other three peaks) (Figure 1D,E). The size distribution pattern of ucf‐eccDNAs is highly conserved across most individual urine samples (Figure S3), which is reminiscent of the size of single, double, triple and quadruple nucleosomes and consistent with previous observations of eccDNAs from plasma and tissues.^33^

### 3.4 Effects of genomic context in cis on ucf‐eccDNAs

**FIGURE 3:**
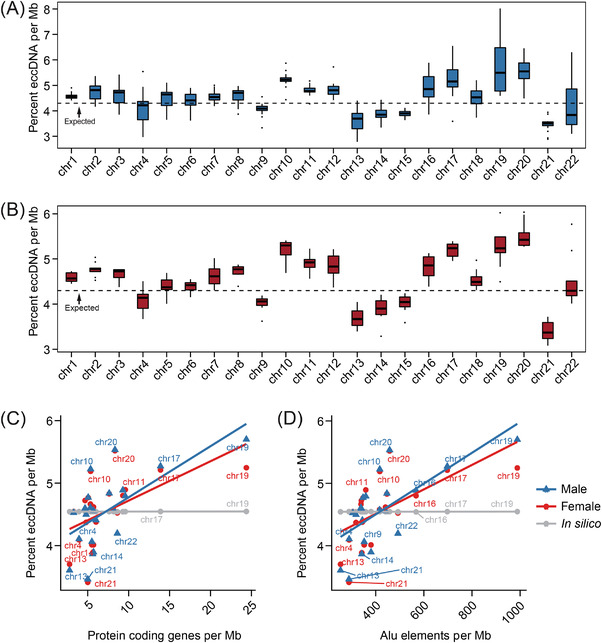
Extrachromosomal circular deoxyribonucleic acid (EccDNA) frequency correlates with chromosome and the density of protein coding genes and *Alu* elements. (A, B) The percent of the number of eccDNAs per Mb in different chromosomes. (C, D) Significant positive correlation between the frequency of eccDNA with the number of protein coding genes per Mb and the number of *Alu* elements per Mb

We next examined the genomic context of ucf‐eccDNAs by aligning all the ucf‐eccDNAs to the human reference genome. The ucf‐eccDNAs were distributed across the entire human genome (Figure 2A). We then combined and merged eccDNA sequences and found that these molecules contained approximately 14.9% (462.1 Mb) of the total human genomic information, supporting that a considerable proportion of the human genome can contribute to the formation of eccDNAs. However, we observed that the frequency of eccDNA generation from each chromosome, calculated by the normalised number of eccDNAs per Mb, was uneven. Specifically, the gene‐rich chromosome 19 hosted more eccDNAs than other chromosomes, whereas the gene‐poor chromosome 21 hosted less (Figure 3A,B). Consistently, there was a positive correlation between the frequency of eccDNA and the number of protein‐coding genes per Mb (male, *p* value = 1.27E‐3, Pearson's R = .64; female, *p* value = .91E‐2, Pearson's R = .65). The correlation of eccDNA formation frequency and (protein‐coding) gene number (Figure 3C) suggests a potential link between transcription activity and eccDNA generation, an observation that was found in previous reports.^15^, ^16^, ^21^, ^34^ Compared with other chromosomes, chromosome 19 is also enriched with *Alu* elements; therefore, we hypothesised that *Alu* elements could be correlated with eccDNA generation. We then compared the frequency of eccDNA and the normalised number of *Alu* elements per Mb. Our analysis showed that there was a positive correlation between eccDNA and *Alu* (male, *p* value = 3.95E‐4, Pearson's R = .69; female, *p* value = 3.15E‐3, Pearson's R = .60) (Figure 3D). Most importantly, there was no significant correlation between the frequency of eccDNA and the number of protein‐coding genes per Mb (*p* value = .14, Pearson's R = .33) or the frequency of eccDNA and the number of *Alu* elements per Mb (*p* value = .19, Pearson's R = .29) in the *in silico*‐generated random eccDNA data sets. Our results suggest a difference in vulnerability to genomic context‐dependent formation of eccDNAs. Together, these results demonstrated that the frequency of eccDNA generation may be influenced by the genomic context*in cis*.

### 3.5 Genomic distribution of ucf‐eccDNAs

**FIGURE 4:**
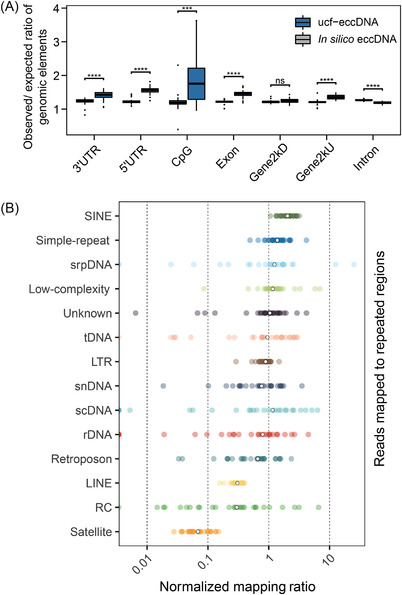
Genomic annotation of ucf‐eccDNAs. (A) Distribution of ucf‐eccDNAs in the indicated genomic elements. The “observed/expected ratio of genomic elements” is the number of eccDNAs falling in a certain type of genomic element divided by the percentage of the length of that genomic element over the length of the whole genome. (B) Normalised mapping ratio of eccDNA reads in specific repetitive elements (median, white dot). Gene2 kbD, 2 kb downstream of genes; Gene2 kbU, 2 kb upstream of genes; LINE, long interspersed nuclear element; LTR, long terminal repeat; ns, not significant; RC, rolling circle repeats. rDNA, ribosomal DNA repeats; scDNA, small conditional DNA repeats; SINE, short interspersed nuclear element; snDNA, small nuclear DNA repeats; srpDNA, signal recognition particle DNA repeats; tDNA, transport DNA repeats; Ucf‐eccDNAs, urinary cell‐free extrachromosomal circular deoxyribonucleic acid; UTR, untranslated region; *** and **** denote significant *p* values less than .001 and .0001, respectively

We further explored the genomic distribution of ucf‐eccDNAs in the human genome. To do that, we mapped the ucf‐eccDNA junctions to seven major classes of genomic elements: untranslated regions (UTR), CpG islands (CpG), exons, introns and 2 kb regions upstream/downstream of genes. Since the length of these genomic elements varied widely, using the absolute number of eccDNA in each type of genomic element for statistics cannot truly reflect its generation preference. Therefore, we used a normalised “observed/expected ratio of genomic elements” to statistically evaluate the enrichment or depletion of ucf‐eccDNAs. The “observed/expected ratio of genomic elements” was calculated as the number of eccDNAs falling in a certain type of genomic element divided by the percentage of the length of that genomic element over the length of the whole genome. Notably, our results showed that the distribution of eccDNAs in the whole genome was not stochastically distributed. Consistent with the analysis above, certain genomic regions are more vulnerable to ucf‐eccDNA formation. In particular, compared with *in silico* eccDNAs, ucf‐eccDNAs were primarily enriched in CpG islands and 5′UTRs (*p* value < .001, Wilcoxon test) and exhibited comparatively low enrichment in intronic regions (Figure 4A).

Repetitive sequences account for 52.5% of the human genome. In human cells and tissues, repetitive sequences are more vulnerable to the formation of eccDNAs.^16^, ^19^, ^35^ Therefore, we extracted ucf‐eccDNA reads from repetitive sequences in the hope of gaining more insights into the relationship between ucf‐eccDNAs and repetitive sequences. The average proportion of ucf‐eccDNA reads that aligned to repetitive elements was 70.7%, corroborating previous findings and supporting the overrepresentation of ucf‐eccDNAs in repetitive sequences. Moreover, we noticed that the enrichment of each repetitive class on ucf‐eccDNA was uneven. Specifically, elements encoding short interspersed nuclear elements (SINEs) (2.03‐fold, median), simple repeats (1.38‐fold, median) and signal recognition particle DNA repeats (srpDNA) (1.21‐fold, median) were clearly overrepresented, whereas satellites (.07‐fold, median), rolling circle repeats (RC) (.29‐fold, median) and long interspersed nuclear elements (LINEs) (.30‐fold, median) were less than expected (Figure 4B). Collectively, our results suggest that certain genomic contexts, such as CpG islands and generic regions, are more enriched in eccDNAs.

### 3.6 Simple direct and palindromic repeats are commonly found at ucf‐eccDNA junctions

**FIGURE 5:**
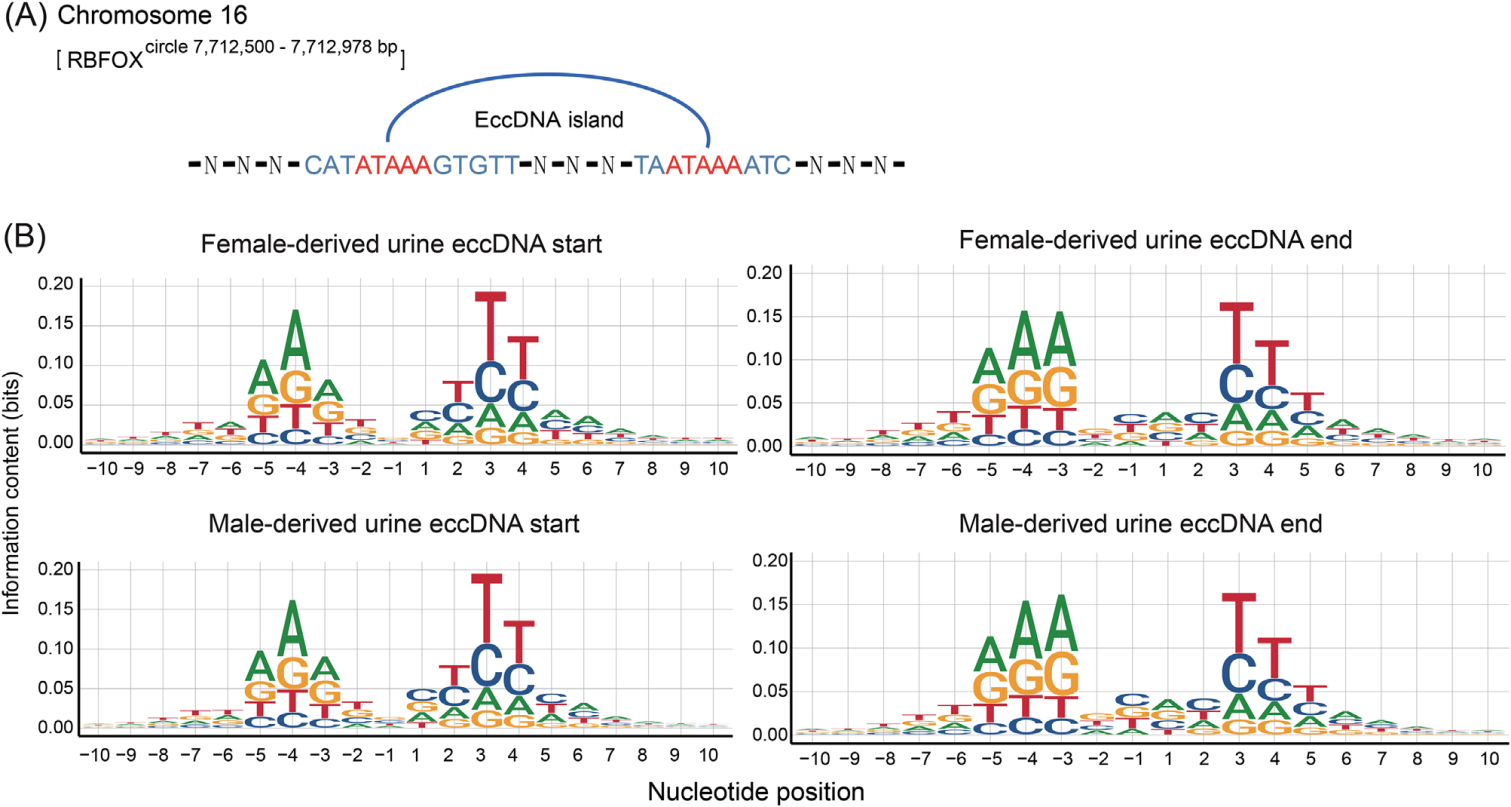
Characterization of ucf‐eccDNA fragment junction sites. (A) As one example, [*RBFOX1* ^circle 7,712,600‐7,712,978^ ^bp^] with 5 bp direct repeats (DR) flanking its start and end sites. (B) The nucleotide frequencies (palindromic repeats) surrounding the start and end sites of male‐derived ucf‐eccDNAs (pooled data from 19 cases) and female‐derived ucf‐eccDNAs (pooled data from 9 cases). Ucf‐eccDNAs: urinary cell‐free extrachromosomal circular deoxyribonucleic acid

We next sought to characterise the sequence motifs of ucf‐eccDNAs, as the generation of eccDNA in cells is associated with the endogenous DNA repair machinery. Previously, we demonstrated that the repair of DNA double‐strand breaks introduced by CRISPR can lead to the generation of eccDNAs in cells.^36^ We focused on the eccDNA start/end site (junction), which was the upstream/downstream edge in the genome domains of eccDNA origin and might be related to circularisation of certain genome domains. Therefore, we scrutinised the DNA sequences from 10 bp flanking at eccDNA start and end sites to investigate the mechanism underlying the formation of ucf‐eccDNAs. Notably, we observed that 66.36% of the total eccDNA molecules possessed 4‐ to 18‐bp direct repeats (DRs) near eccDNA junction sites. For example, [*RBFOX1* ^circle 7,712,600‐7,712,978^ ^bp^] with a fragment size of 378 bp had a 5 bp DR near the eccDNA junction site (Figure 5A). Moreover, we also found a pair of trinucleotide panlindromic repeats with 4‐bp “spacers” in between flanking the eccDNA start and end sites, which was similar to the motif signature of plasma eccDNA (Figure 5B).^22^ We then divided eccDNA molecules into bins by their peak sizes and found that this trinucleotide palindromic repeat motif pattern was also conserved regardless of fragment size (Figure S4), suggesting a potential role of palindromic repeats in the biogenesis of eccDNAs.

### 3.7 Ucf‐eccDNA profiles in patients with advanced CKD

**FIGURE 6:**
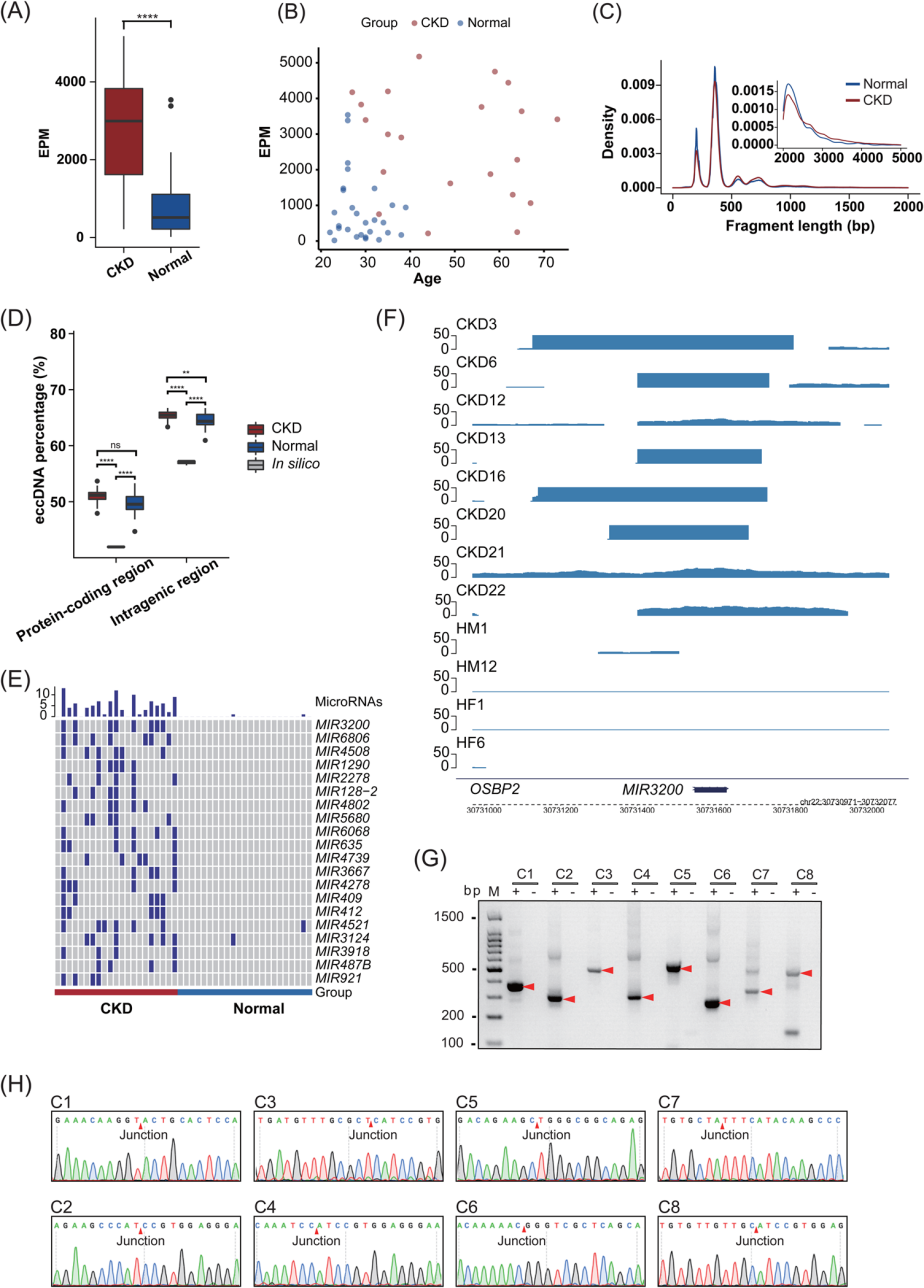
Differential ucf‐eccDNA profiles in patients with advanced CKD. (A) Comparison of the number of ucf‐eccDNA per million mapped reads (EPM) between the CKD group and the healthy control group. (B) Correlation analysis between EPM and age in all samples. (C) Fragment length distribution of ucf‐eccDNA in the CKD group and healthy control group. (D) The proportion of ucf‐eccDNA mapped to protein coding/genetic regions between the CKD group and the healthy control group. (E) Top 20 miRNAs affected by circularization in CKD urine samples. (F) The genome browser track at the MIR3200 regions from Circle‐Seq (G) PCR validation of all MIR3200‐related ucf‐eccDNAs. (H) Junction sites obtained after sequencing of PCR products. CKD: chronic kidney disease; ns, not significant; Ucf‐eccDNA: urinary cell‐free extrachromosomal circular deoxyribonucleic acid; ** and **** denote significant *p* values less than .01 and .0001, respectively

Given that ucf‐eccDNAs are commonly present in human urine, we sought to investigate whether ucf‐eccDNAs could be used as a biomarker for pathological conditions. In this regard, we collected urine samples from 21 patients with advanced CKD (stage 3–5) and attempted to explore the potential clinical application of ucf‐eccDNAs. CKD stage was assessed based on the EGFR. Using the optimised Circle‐Seq protocol, we detected 4,952,777 unique ucf‐eccDNAs from all CKD urine samples (average: 235,847, range 19,051‐455,436) (Table S2). As seen in Figures S5 and S6, ucf‐eccDNAs from advanced CKD patients exhibit a profile similar to that of controls in terms of GC content, genomic distribution and motif signature. However, the EPM of the CKD group was significantly higher than that of the healthy control group (CKD: mean, 2760; 95% CI, 2074 ‐ 3446 vs. healthy control: mean, 861; 95% CI, 495 ‐ 1227) (*p* value < .0001, Wilcoxon test) (Figure 6A). Since most CKD patients were older than the healthy controls, to investigate whether the increased EPM in CKD was caused by age, we performed correlation analysis between EPM and age in all samples. No significant correlation (CKD: Pearson's R = .16, *p* value = .476; healthy control: Pearson's R = .22, *p* value = .257) was observed between EPM and age in control and CKD samples (Figure 6B). Meanwhile, we noticed that ucf‐eccDNAs from CKD patients had larger circles than those from the healthy control group, although the difference was not particularly obvious (Figure 6C). In addition, compared with ucf‐eccDNAs in healthy individuals, CKD‐derived ucf‐eccDNAs are more likely generated from intragenic regions (*p* value < .01, Wilcoxon test) (Figure 6D). Because the ucf‐eccDNAs are too small to carry full protein‐coding genes, we analysed the miRNA gene ucf‐eccDNAs. The miRNA ucf‐eccDNAs denote a group of circles carrying full miRNA‐coding sequences, which could be a stable detection target for diagnosis. Surprisingly, we found that a number of miRNA ucf‐eccDNAs are frequently detected in CKD patients (Table S3), and the top 20 high frequency miRNAs are displayed in Figure 6F. We selected the top miRNA‐related (MIR3200) ucf‐eccDNAs, and these circles were all successfully validated by PCR and Sanger sequencing (Figure 6E‐H). Taken together, our results reveal that patients with advanced CKD have a significantly higher number of ucf‐eccDNAs in their urine.

## 4 DISCUSSION

eccDNA is commonly found in plasma and somatic tissues.^16^, ^21^, ^22^, ^23^ However, its presence in human urine is still largely unclear. This study demonstrated for the first time that cell‐free eccDNAs are commonly found in urine. These ucf‐eccDNAs are likely derived from the kidney and the urinary tract, as well as plasma eccDNAs, which may cross the filtration barrier into Bowman's capsule. This also implies a possible mechanism for the clearance of circulating circular DNA. Although several studies have reported the existence and characteristics of plasma eccDNA, it has been unclear how eccDNA is cleared from the plasma.^22^, ^23^, ^24^, ^25^ Our results suggest that filtration of eccDNA in plasma to the urinary system might be one potential route for eccDNA to be eliminated from the body, which should be addressed in future studies.

We identified millions of ucf‐eccDNAs, and these eccDNA molecules are widely distributed across the human genome. The combined eccDNA (in healthy individuals) lengths covered 14.9% of the human genome, suggesting that a considerable proportion of the genomic regions can form eccDNA. Chromosomes with a higher density of (protein‐coding) genes more frequently form eccDNA, corroborating the findings between eccDNA generation and gene transcription activity. Recent studies reveal that eccDNAs are products of, for example, cellular apoptosis,^37^ chromosome breakage (chromothripsis),^38^ transcription activity and error,^39^, ^40^ replication error and DNA mismatch repair and damage response.^41^, ^42^ In the human genome, rDNA repeats and tDNA repeats are more likely to generate circles than any other repetitive elements, and satellites on eccDNAs were also overrepresented.^16^, ^19^ However, in human urine, satellites on eccDNAs were clearly less than expected (.1‐fold, median), and SINEs preferentially give rise circles than rDNA repeats and tDNA repeats, suggesting that eccDNA formation exhibited genomic context‐specific vulnerability.

It has been reported that urine‐derived cell‐free liner DNA is highly degraded, with its main fragment length ranging from 80 to 110 bp.^3^, ^43^ Here, we found that the eccDNA number among each urine sample varied widely. We therefore speculated that cell‐free eccDNAs are also degraded to some extent in the urine, although circular DNA is more stable than linear DNA. Further studies are still needed to systematically investigate the stability of urine cell‐free eccDNAs. Moreover, ucf‐eccDNAs were significantly longer than their linear counterparts. The fragment length distribution of ucf‐eccDNAs presented four appreciable peak clusters located at 207, 358, 553 and 732 bp (Figure 1D). Such a length profile is quite similar to the length distribution of plasma eccDNAs.^21^, ^22^ The fragment patterns of urinary cfDNA were previously proven to be associated with nucleosome degradation.^3^ The nucleosome consists of two linkers (∼40 bp) and a core region (∼146 bp). We noticed that ucf‐eccDNAs showed a 160‐200 bp periodicity, suggesting that ucf‐eccDNA may be derived from nucleosomes.

Several mechanisms have been found and suggested to regulate eccDNA generation. Several studies suggested that repair of double‐stranded DNA breaks through microhomology mediated end joining (MMEJ) may mediate eccDNA formation because a large portion of circles had various repeated sequences (4‐18 bp) at their junction sites.^15^, ^16^, ^18^ A still large fraction of eccDNA molecules did not contain any repeat sequences and thus it is possible that other DNA repair pathways are involved in the regulation of eccDNA formation, such as nonhomologous end joining. Dual palindromic repeats flanking the junction sites of eccDNA were observed in this study, and this motif pattern was quite similar to that in plasma eccDNA,^22^ which will serve as microhomology for the generation of eccDNAs. All these features indicated that R‐loop formation and replication slippage may also lead to eccDNA formation (based on single‐stranded DNA).^15^, ^22^ In addition, since inverted repeats and palindromic repeats could be DNA binding motifs for DNA transposons,^44^ this inevitably leads to the postulation of a possible “enzymatic mechanism” in the generation of eccDNAs in cells.

We expect that the identification and characterisation of eccDNA in urine will allow us to explore the potential applications of ucf‐eccDNAs as noninvasive biomarkers for disease diagnosis and monitoring. In this study, we analysed ucf‐eccDNAs from patients with advanced CKD. The EGFR is commonly used in clinical practice to estimate renal function. However, this is more accurate with advanced renal failure and affected by the individual's ages and other comorbidities.^45^, ^46^ The normalized number of ucf‐eccDNA and, to a lesser extent, the eccDNA size in CKD patients is significantly higher than that in healthy controls. These results may be explained by the destruction of the glomerular filtration barrier,^28^, ^29^ which leads to more plasma eccDNAs crossing the filtration barrier into the urine. Meanwhile, since renal cellular apoptosis, DNA damage and inflammation occurred in advanced CKD patients, the CKD ucf‐eccDNAs detected in our study most likely contain eccDNAs from damaged kidney cells, although we could not distinguish plasma‐origin and renal original eccDNAs with the current Circle‐Seq method. Another interesting finding by profiling CKD ucf‐eccDNA is the enrichment of eccDNAs that contain miRNA binding sequences. Urinary miRNA is supposed to be an attractive biomarker for liquid biopsy.^47^ Our study also identified a number of miRNA gene eccDNAs that are frequently enriched in urine samples from CKD patients. Although the origin (from which cells) and function (pathogenesis in CKD) of these miRNA gene ucf‐eccDNAs are unclear and should be addressed in future studies, the consistent enrichment of miRNA genes in ucf‐eccDNAs (i.e., MIR3200, MIR6806, MIR4508) across several CKD patients is a hallmark indicating the dysregulated transcriptional regulation machinery in CKD patients. A number of the top 20 enriched miRNA‐encoding genes in the eccDNAs (i.e., MIR4508, MIR1290, MIR2278, MIR409 and MIR4521) have been reported to be associated with renal and urinary disorders.^48^, ^49^, ^50^, ^51^, ^52^, ^53^ For example, serum MIR4508 was abnormal in patients with transplant glomerulopathy.^48^ MIR1290 mediated early molecular events related to ischemia/reperfusion injury in kidney transplantation.^49^ Since eccDNAs are apoptotic products with high innate immunostimulatory activity,^37^ the increase in ucf‐eccDNAs in CKD is in good agreement with the pathogeneses of cell death, chronic inflammation, and activated innate immunity commonly appearing in CKD patients.^54^, ^55^ Collectively, our study suggests ucf‐eccDNA as an attractive biomarker for urogenital disease diagnosis and monitoring, such as CKD. However, we also highlight the limitation that our study only included a relatively small sample size and lacked early‐stage CKD patients. This phenomenon remains to be addressed in a large cohort in further studies.

## 5 CONCLUSIONS

This study reports that cell‐free eccDNA is common in human urine and systematically describes the features (e.g., genomic distribution, size profiles, GC content and motif signatures) of these special types of DNA molecules. Moreover, compared with ucf‐eccDNAs from healthy individuals, CKD patient‐derived ucf‐eccDNAs displayed distinct genomic profiles. These interesting features of ucf‐eccDNAs may provide an important reference for the further exploration of ucf‐eccDNAs as diagnostic biomarkers for multiple urogenital disorders.

## CONFLICT OF INTERESTS

The authors declare that they have no known competing financial interests or personal relationships that could have appeared to influence the work reported in this study.

## Supporting information


**Figure S1** Quality control of RCA products by agarose gel electrophoresis (0.7%). (A) Gel image of RCA products. (B) Gel image of the double‐digested RCA products with MSMSSI and NotI restriction enzymes. Urine samples from healthy males and healthy females are marked with blue and red, respectively. RCA: rolling circle amplification.
**Figure S2** Analysis of the correlation between the length of coding gene and the number of eccDNA.
**Figure S3** Fragment length distribution of ucf‐eccDNAs from 28 healthy individual cases. HF: healthy female; HM: healthy male; Ucf‐eccDNAs: urinary cell‐free eccDNAs.
**Figure S4** The nucleotide frequencies surrounding the start and end sites of ucf‐eccDNAs with different peak sizes. Ucf‐eccDNAs: urinary cell‐free eccDNAs
**Figure S5** Fragment length distribution of ucf‐eccDNAs from 21 patients with advanced CKD. CKD: chronic kidney disease; Ucf‐eccDNAs: urinary cell‐free eccDNAs.
**Figure S6** Basic genomic and sequence features of ucf‐eccDNAs from the CKD group. (A) Fragment length distribution and (B) GC content distribution of ucf‐eccDNAs (pooled data from 21 cases). (C) Distribution of ucf‐eccDNAs in the indicated genomic elements. (D) Normalised mapping ratio of eccDNA reads in specific repetitive elements (median, white dot). (E) The nucleotide frequencies surrounding the start and end sites of ucf‐eccDNAs. CKD: chronic kidney disease; Ucf‐eccDNAs: urinary cell‐free eccDNA.
**Table S1**. The number of ucf‐eccDNAs in each healthy volunteer. Ucf‐eccDNA: urinary cell‐free eccDNA.
**Table S2**. The number of ucf‐eccDNAs in each CKD patient. CKD: chronic kidney disease; Ucf‐eccDNA: urinary cell‐free eccDNA.
**Table S3**. List of ucf‐eccDNA‐related miRNAs that frequently occurred in CKD urine samples.
**Table S4**. Description of MIR3200‐eccDNAs validated in Figure 6F‐H.

## Data Availability

The data that support the findings of this study have been deposited into CNGB Sequence Archive (CNSA) of China National GeneBank DataBase (CNGBdb) with accession number CNP0002377 and CNP0002782.
