# Supplementary material for: Circle‐Seq reveals genomic and disease‐specific hallmarks in urinary cell‐free extrachromosomal circular DNAs
Source: Clin Transl Med. 2022 Apr 26;12(4):e817. doi: 10.1002/ctm2.817 (PMC9042798; doi:10.1002/ctm2.817)
Supplement: Supplementary file 1 — Figure S1 Quality control of RCA products by agarose gel electrophoresis (0.7%). (A) Gel image of RCA products. (B) Gel image of the double‐digested RCA products with MSMSSI and NotI restriction enzymes. Urine samples from healthy males and healthy females are marked with blue and red, respectively. RCA: rolling circle amplification. Figure S2 Analysis of the correlation between the length of coding gene and the number of eccDNA. Figure S3 Fragment length distribution of ucf‐eccDNAs from 28 healthy individual cases. HF: healthy female; HM: healthy male; Ucf‐eccDNAs: urinary cell‐free eccDNAs. Figure S4 The nucleotide frequencies surrounding the start and end sites of ucf‐eccDNAs with different peak sizes. Ucf‐eccDNAs: urinary cell‐free eccDNAs Figure S5 Fragment length distribution of ucf‐eccDNAs from 21 patients with advanced CKD. CKD: chronic kidney disease; Ucf‐eccDNAs: urinary cell‐free eccDNAs. Figure S6 Basic genomic and sequence features of ucf‐eccDNAs from the CKD group. (A) Fragment length distribution and (B) GC content distribution of ucf‐eccDNAs (pooled data from 21 cases). (C) Distribution of ucf‐eccDNAs in the indicated genomic elements. (D) Normalised mapping ratio of eccDNA reads in specific repetitive elements (median, white dot). (E) The nucleotide frequencies surrounding the start and end sites of ucf‐eccDNAs. CKD: chronic kidney disease; Ucf‐eccDNAs: urinary cell‐free eccDNA. Table S1. The number of ucf‐eccDNAs in each healthy volunteer. Ucf‐eccDNA: urinary cell‐free eccDNA. Table S2. The number of ucf‐eccDNAs in each CKD patient. CKD: chronic kidney disease; Ucf‐eccDNA: urinary cell‐free eccDNA. Table S3. List of ucf‐eccDNA‐related miRNAs that frequently occurred in CKD urine samples. Table S4. Description of MIR3200‐eccDNAs validated in Figure 6F‐H. [file CTM2-12-e817-s001.pdf]

Figure S1

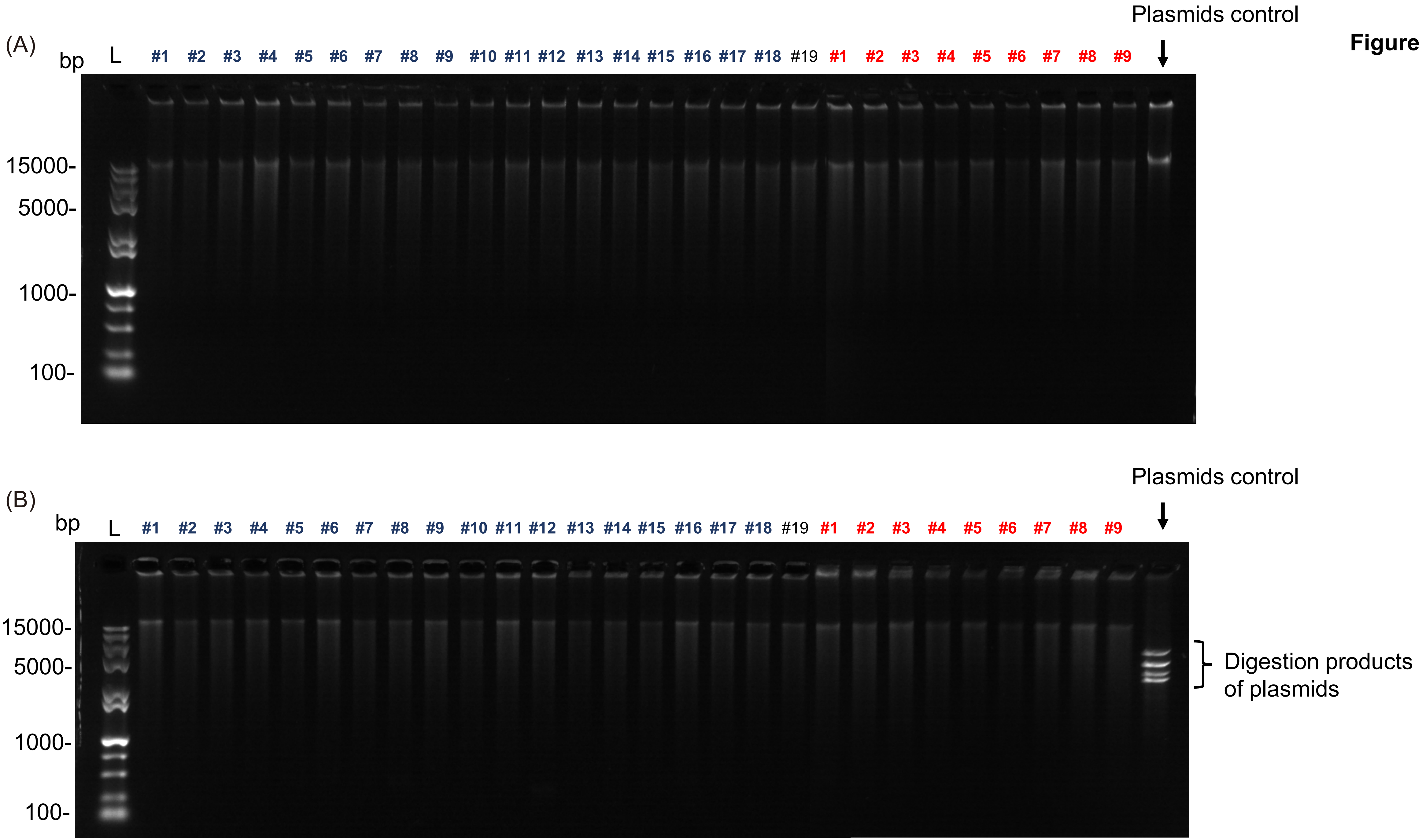
(A)
Plasmids control
bp
L
#1 #2 #3 #4 #5 #6 #7 #8 #9 #10 #11 #12 #13 #14 #15 #16 #17 #18 #19 #1 #2 #3 #4 #5 #6 #7 #8 #9
15000-
5000-
1000-
100-
(B)
Plasmids control
bp
L
#1 #2 #3 #4 #5 #6 #7 #8 #9 #10 #11 #12 #13 #14 #15 #16 #17 #18 #19 #1 #2 #3 #4 #5 #6 #7 #8 #9
15000-
5000-
1000-
100-
Digestion products of plasmids

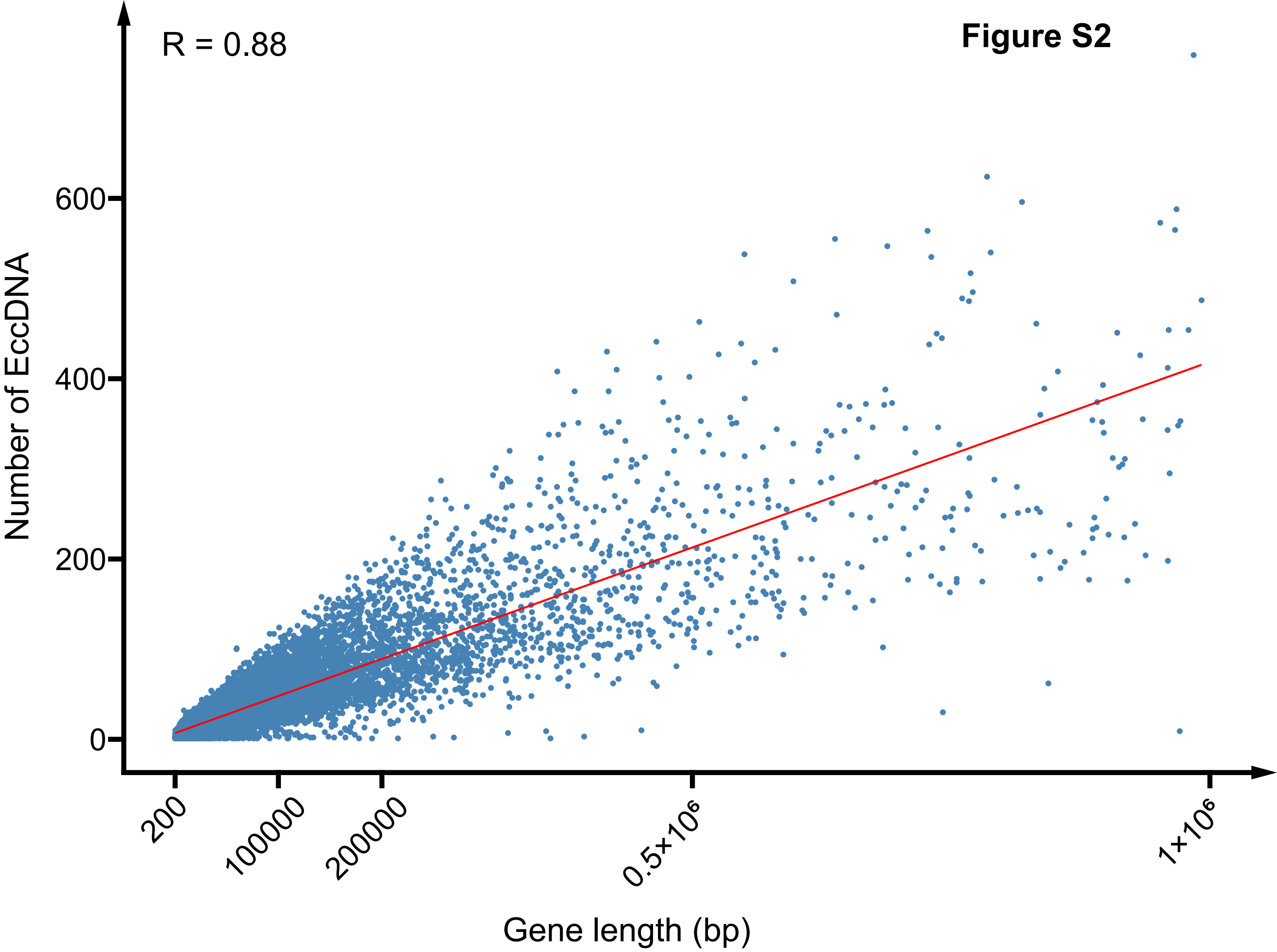

Figure S2
R = 0.88
Number of EccDNA
600
400
200
0
200
100000
200000
0.5×10^6
1×10^6
Gene length (bp)

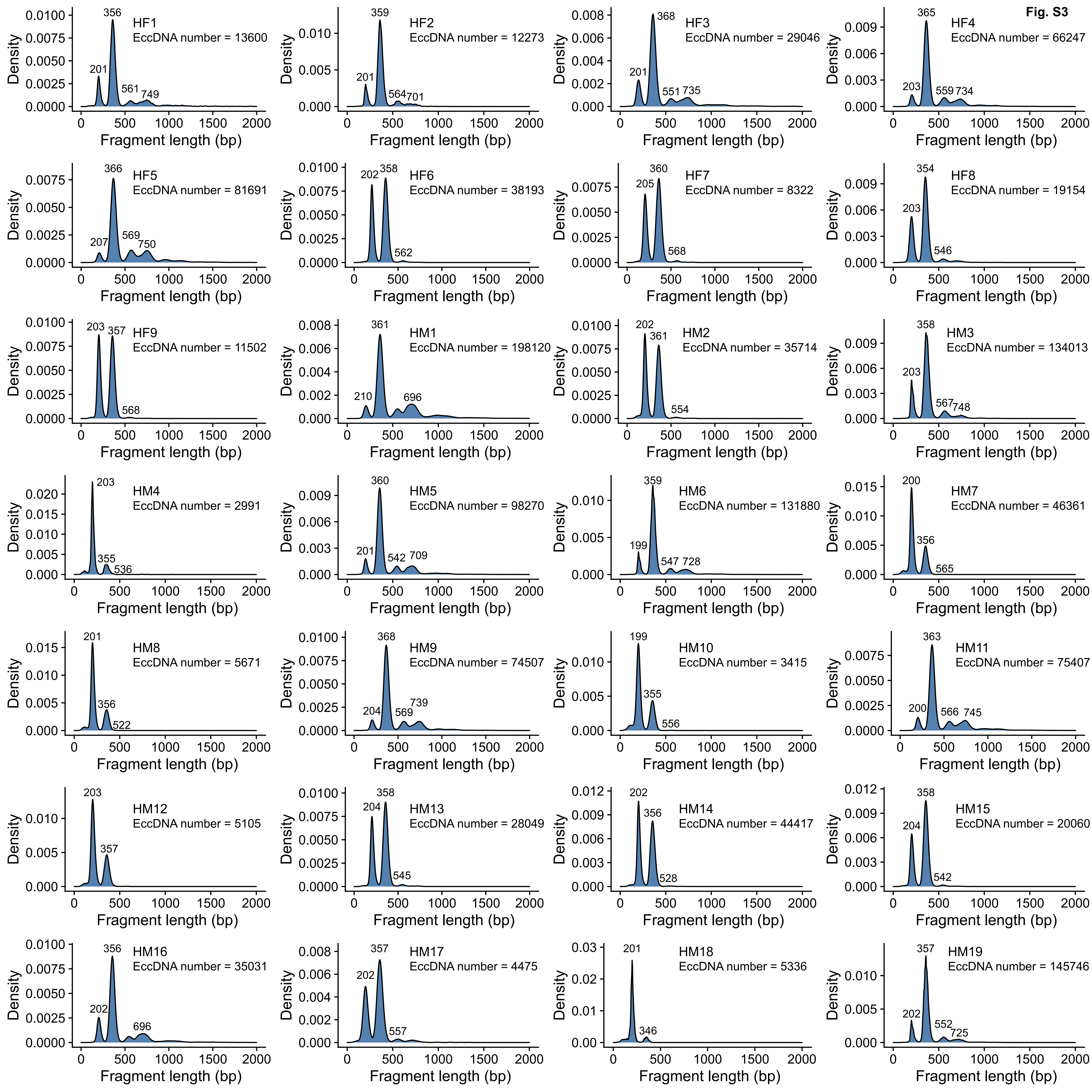
HF1
EccDNA number = 13600
HF2
EccDNA number = 12273
HF3
EccDNA number = 29046
HF4
EccDNA number = 66247
HF5
EccDNA number = 81691
HF6
EccDNA number = 38193
HF7
EccDNA number = 8322
HF8
EccDNA number = 19154
HF9
EccDNA number = 11502
HM1
EccDNA number = 198120
HM2
EccDNA number = 35714
HM3
EccDNA number = 134013
HM4
EccDNA number = 2991
HM5
EccDNA number = 98270
HM6
EccDNA number = 131880
HM7
EccDNA number = 46361
HM8
EccDNA number = 5671
HM9
EccDNA number = 74507
HM10
EccDNA number = 3415
HM11
EccDNA number = 75407
HM12
EccDNA number = 5105
HM13
EccDNA number = 28049
HM14
EccDNA number = 44417
HM15
EccDNA number = 20060
HM16
EccDNA number = 35031
HM17
EccDNA number = 4475
HM18
EccDNA number = 5336
HM19
EccDNA number = 145746
Density
Fragment length (bp)
Fig. S3

Figure S4

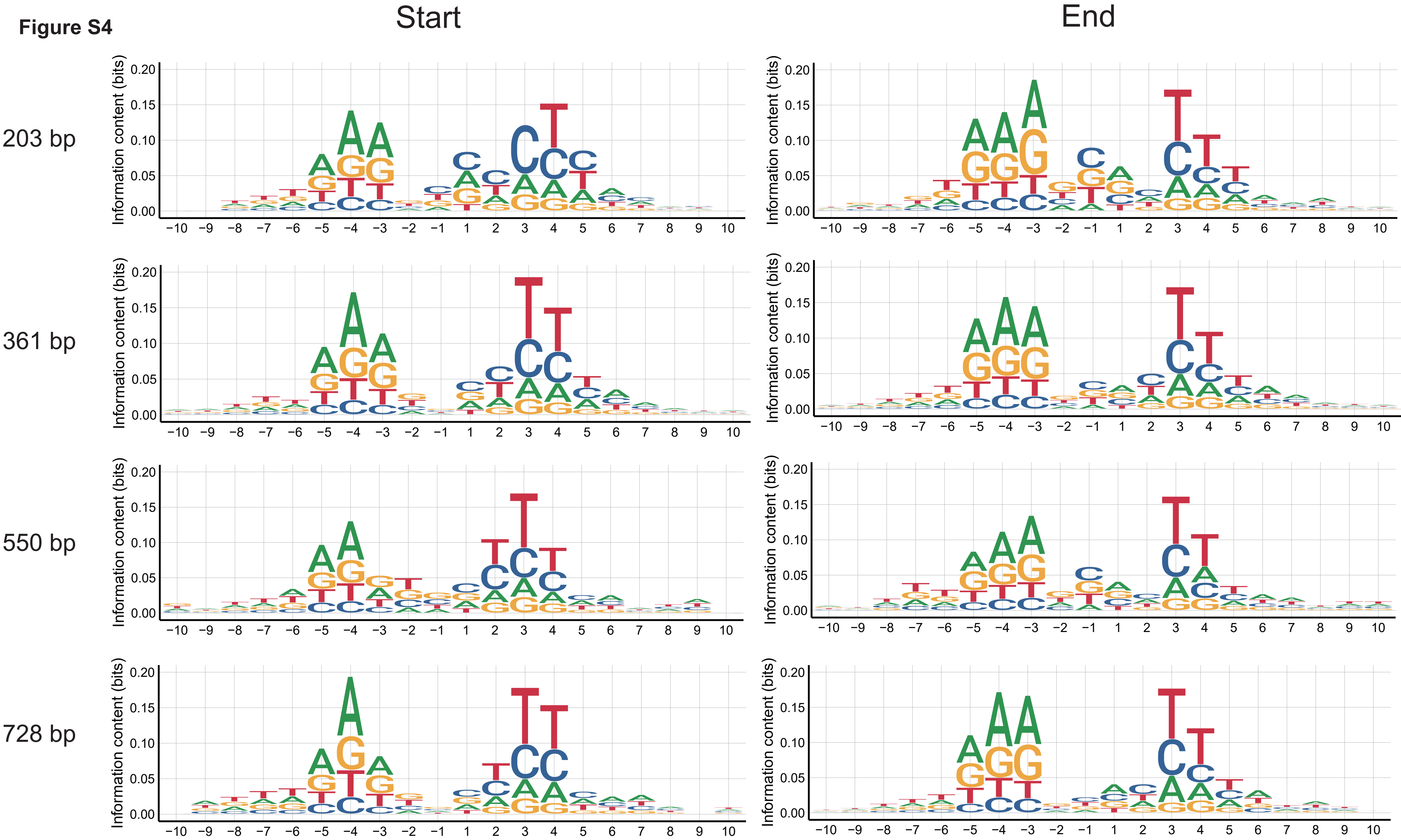
Start
End
203 bp
361 bp
550 bp
728 bp
Information content (bits)

Fig. S5

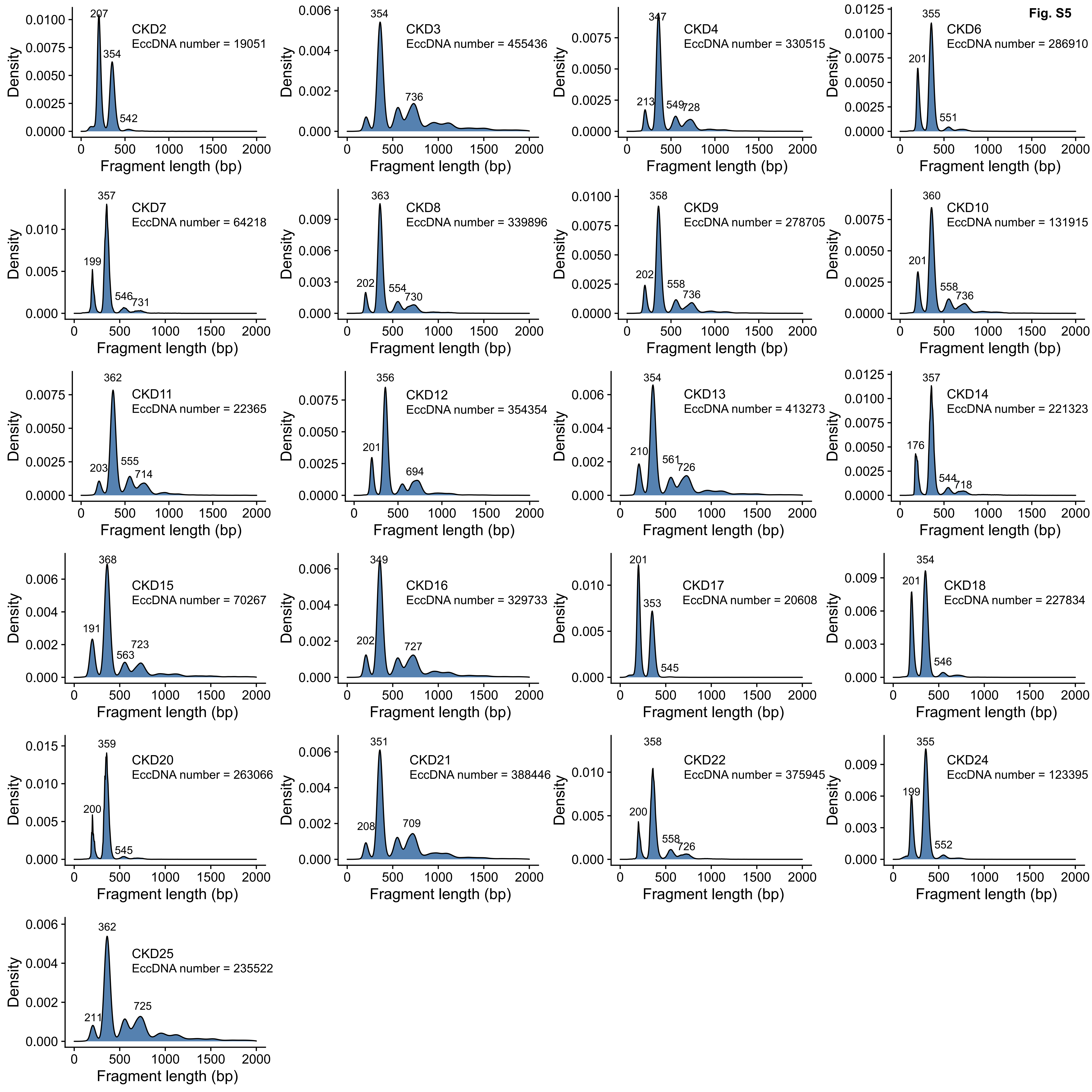
CKD2
EccDNA number = 19051
CKD3
EccDNA number = 455436
CKD4
EccDNA number = 330515
CKD6
EccDNA number = 286910
CKD7
EccDNA number = 64218
CKD8
EccDNA number = 339896
CKD9
EccDNA number = 278705
CKD10
EccDNA number = 131915
CKD11
EccDNA number = 22365
CKD12
EccDNA number = 354354
CKD13
EccDNA number = 413273
CKD14
EccDNA number = 221323
CKD15
EccDNA number = 70267
CKD16
EccDNA number = 329733
CKD17
EccDNA number = 20608
CKD18
EccDNA number = 227834
CKD20
EccDNA number = 263066
CKD21
EccDNA number = 388446
CKD22
EccDNA number = 375945
CKD24
EccDNA number = 123395
CKD25
EccDNA number = 235522
Density
Fragment length (bp)

Figure S6

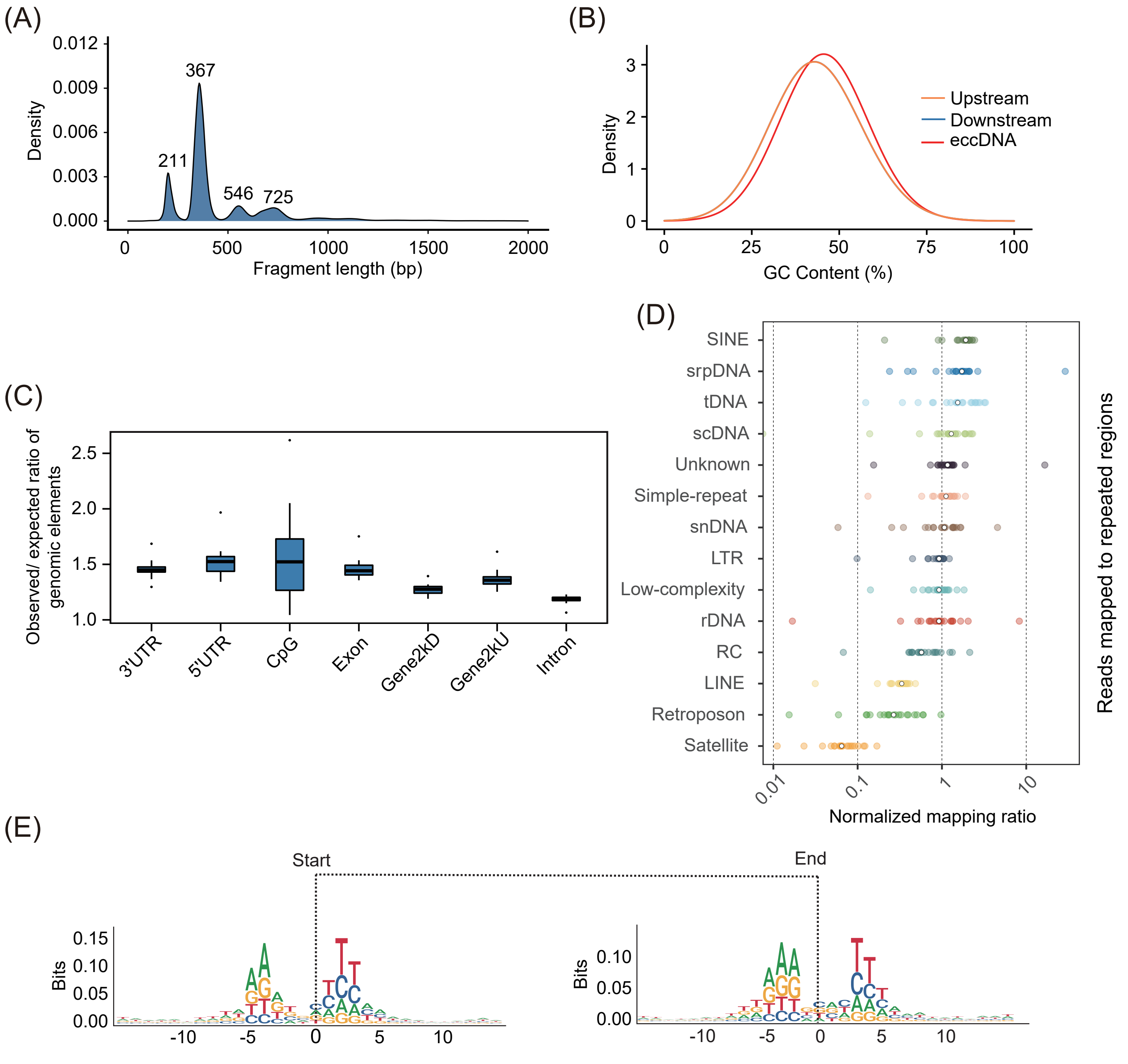
(A)
0.012
0.009
0.006
0.003
0.000
Density
367
211
546
725
0
500
1000
1500
2000
Fragment length (bp)
(B)
3
2
1
0
Density
Upstream
Downstream
eccDNA
0
25
50
75
100
GC Content (%)
(C)
Observed/ expected ratio of genomic elements
2.5
2.0
1.5
1.0
3'UTR
5'UTR
CpG
Exon
Gene2kD
Gene2kU
Intron
(D)
SINE
srpDNA
tDNA
scDNA
Unknown
Simple-repeat
snDNA
LTR
Low-complexity
rDNA
RC
LINE
Retroposon
Satellite
0.01
0.1
1
10
Normalized mapping ratio
Reads mapped to repeated regions
(E)
Start
End
Bits
0.15
0.10
0.05
0.00
-10
-5
0
5
10

Table S1: The number of ucf-eccDNAs in each healthy volunteers. Ucf-eccDNA: urinary cell free eccDNA.

Sample#	Gender	Age	ucf-eccDNA#
HF1	Female	22	198120
HF2	Female	26	35714
HF3	Female	24	134013
HF4	Female	26	2991
HF5	Female	26	98270
HF6	Female	32	131880
HF7	Female	34	46361
HF8	Female	35	5671
HF9	Female	26	74507
HM1	Male	26	3415
HM2	Male	38	75407
HM3	Male	39	5105
HM4	Male	33	28049
HM5	Male	32	44417
HM6	Male	25	20060
HM7	Male	29	35031
HM8	Male	31	4475
HM9	Male	36	5336
HM10	Male	30	145746
HM11	Male	27	13600
HM12	Male	28	12273
HM13	Male	28	29046
HM14	Male	24	66247
HM15	Male	30	81691
HM16	Male	23	38193
HM17	Male	30	8322
HM18	Male	23	19154
HM19	Male	25	11502

Table S2. The number of ucf-eccDNAs in each CKD patients. Ucf-eccDNA: urinary cell free eccDNA; CKD: chronic kidney disease.

Sample#	Gender	Age	Stage	ucf-eccDNA#
CKD2	Male	44	5	19051
CKD3	Male	35	5	455436
CKD4	Male	56	5	330515
CKD6	Female	35	5	286910
CKD7	Male	33	4	64218
CKD8	Male	65	3	339896
CKD9	Male	38	5	278705
CKD10	Female	49	5	131915
CKD11	Female	58	5	22365
CKD12	Male	42	4	354354
CKD13	Male	27	5	413273
CKD14	Female	29	5	221323
CKD15	Female	63	5	70267
CKD16	Female	73	5	329733
CKD17	Male	64	5	20608
CKD18	Female	34	5	227834
CKD20	Male	64	5	263066
CKD21	Male	59	5	388446
CKD22	Female	30	5	375945
CKD24	Male	67	5	123395
CKD25	Female	62	5	235522

Table S3. List of ucf-eccDNA-related miRNAs frequently occurred in CKD urine samples.

eccDNA	Chromosome	Start	End	Discordant	Splits	MiRNA	eccDNA size (bp)	Sample
chr22:29333119-29334121	chr22	29333119	29334121	7	8	AC002059.2	1002	CKD12
chr14:75604137-75604371	chr14	75604137	75604371	0	15	AC007182.3	234	CKD18
chr3:24521117-24521454	chr3	24521117	24521454	0	7	AC012087.1	337	CKD8
chr3:24519268-24521455	chr3	24519268	24521455	9	10	AC012087.1	2187	CKD12
chr3:24521204-24521452	chr3	24521204	24521452	0	6	AC012087.1	248	CKD12
chr3:24521068-24521478	chr3	24521068	24521478	28	15	AC012087.1	410	HM1
chr5:54075394-54075773	chr5	54075394	54075773	9	12	AC016601.1	379	HF1
chr8:100023759-100024118	chr8	100023759	100024118	24	27	AC021590.1	359	CKD13
chr8:100023721-100024539	chr8	100023721	100024539	4	9	AC021590.1	818	CKD16
chr12:128244380-128245280	chr12	128244380	128245280	4	16	AC061709.1	900	CKD25
chr4:78820513-78820877	chr4	78820513	78820877	8	8	AC098818.3	364	CKD8
chr4:78820612-78820951	chr4	78820612	78820951	11	12	AC098818.3	339	HF5
chr1:52928043-52929081	chr1	52928043	52929081	12	30	AC099677.4	1038	CKD12
chr1:52928276-52928955	chr1	52928276	52928955	21	9	AC099677.4	679	CKD21
chr1:52940270-52940634	chr1	52940270	52940634	0	4	AC099677.5	364	CKD4
chr1:52940133-52940481	chr1	52940133	52940481	0	10	AC099677.5	348	CKD22
chr1:52940157-52940479	chr1	52940157	52940479	8	11	AC099677.5	322	HM6
chr3:50172665-50176216	chr3	50172665	50176216	64	121	AC104450.1	3551	CKD15
chr20:57895163-57895523	chr20	57895163	57895523	14	6	AL162291.1	360	CKD9
chr20:57895221-57895555	chr20	57895221	57895555	8	26	AL162291.1	334	CKD9
chr20:57895156-57895506	chr20	57895156	57895506	3	36	AL162291.1	350	CKD18
chr20:57895240-57895623	chr20	57895240	57895623	5	8	AL162291.1	383	HF5
chr20:57895240-57895623	chr20	57895240	57895623	15	79	AL162291.1	383	HM19
chr1:10227545-10227893	chr1	10227545	10227893	2	9	AL358013.1	348	CKD10
chr10:68759133-68759492	chr10	68759133	68759492	3	11	AL513534.1	359	CKD3
chr10:23393126-23393478	chr10	23393126	23393478	4	5	AL606469.2	352	CKD6
chr10:23393028-23393527	chr10	23393028	23393527	3	19	AL606469.2	499	CKD13
chr10:23393083-23393612	chr10	23393083	23393612	3	12	AL606469.2	529	CKD16
chr10:23393341-23393532	chr10	23393341	23393532	4	13	AL606469.2	191	HM6
chr11:119018836-119019345	chr11	119018836	119019345	300	326	AP003392.15	509	HM16
chr20:3916680-3918157	chr20	3916680	3918157	12	15	MIR103A2	1477	CKD15
chr20:3917168-3918032	chr20	3917168	3918032	10	25	MIR103A2	864	HM1
chr20:3916680-3918157	chr20	3916680	3918157	12	15	MIR103B2	1477	CKD15
chr20:3917168-3918032	chr20	3917168	3918032	10	25	MIR103B2	864	HM1
chrX:152391936-152392644	chrX	152391936	152392644	6	15	MIR105-1	708	CKD15
chrX:152391732-152392327	chrX	152391732	152392327	0	6	MIR105-1	595	HF5
chrX:134170077-134170741	chrX	134170077	134170741	31	73	MIR106A	664	CKD6
chr10:89592607-89593121	chr10	89592607	89593121	2	15	MIR107	514	CKD3
chr10:89592223-89593023	chr10	89592223	89593023	2	11	MIR107	800	CKD16
chr17:48579268-48580006	chr17	48579268	48580006	4	4	MIR10A	738	CKD21
chr2:176150228-176150534	chr2	176150228	176150534	0	6	MIR10B	306	CKD25
chr12:119713463-119713986	chr12	119713463	119713986	28	14	MIR1178	523	CKD12
chr12:119713577-119713966	chr12	119713577	119713966	12	38	MIR1178	389	CKD12
chr12:119713473-119713873	chr12	119713473	119713873	14	19	MIR1178	400	HM6
chr12:119713258-119713987	chr12	119713258	119713987	662	1188	MIR1178	729	HM11
chr1:231019449-231020212	chr1	231019449	231020212	15	24	MIR1182	763	CKD12
chr1:231019763-231020842	chr1	231019763	231020842	6	8	MIR1182	1079	CKD12
chr1:231019778-231020305	chr1	231019778	231020305	22	37	MIR1182	527	CKD13
chr1:231019794-231020168	chr1	231019794	231020168	9	9	MIR1182	374	HM19
chr14:101042901-101044351	chr14	101042901	101044351	30	10	MIR1185-1	1450	CKD13
chr14:101042762-101043122	chr14	101042762	101043122	28	22	MIR1185-1	360	CKD21
chr14:101042785-101043154	chr14	101042785	101043154	24	6	MIR1185-1	369	CKD21
chr14:101042798-101043612	chr14	101042798	101043612	20	20	MIR1185-1	814	CKD25
chr14:101042769-101043117	chr14	101042769	101043117	103	513	MIR1185-1	348	HM14
chr14:101042901-101044351	chr14	101042901	101044351	30	10	MIR1185-2	1450	CKD13
chr14:101043524-101044356	chr14	101043524	101044356	27	92	MIR1185-2	832	CKD22
chr14:101043210-101044556	chr14	101043210	101044556	1	15	MIR1185-2	1346	CKD25
chr14:101029991-101030340	chr14	101029991	101030340	3	34	MIR1193	349	CKD3
chr14:101024993-101025719	chr14	101024993	101025719	21	44	MIR1197	726	CKD4
chr14:101025330-101025665	chr14	101025330	101025665	0	8	MIR1197	335	CKD21
chr7:36918497-36919755	chr7	36918497	36919755	12	16	MIR1200	1258	CKD3
chr7:36919123-36919784	chr7	36919123	36919784	10	7	MIR1200	661	CKD3
chr7:36919293-36919643	chr7	36919293	36919643	4	11	MIR1200	350	CKD10
chr7:36918787-36919807	chr7	36918787	36919807	3	15	MIR1200	1020	CKD12
chr6:155946651-155947002	chr6	155946651	155947002	32	32	MIR1202	351	HM5
chr17:48156193-48156862	chr17	48156193	48156862	38	152	MIR1203	669	HM1
chr8:127795852-127796262	chr8	127795852	127796262	10	6	MIR1204	410	CKD9
chr8:127795745-127796475	chr8	127795745	127796475	5	25	MIR1204	730	CKD12
chr8:127795743-127796291	chr8	127795743	127796291	8	14	MIR1204	548	CKD22
chr8:128008302-128009778	chr8	128008302	128009778	3	8	MIR1206	1476	CKD13
chr8:128048981-128049315	chr8	128048981	128049315	2	4	MIR1207	334	CKD12
chr8:128048793-128049420	chr8	128048793	128049420	70	58	MIR1207	627	CKD21
chr8:128149709-128150718	chr8	128149709	128150718	3	37	MIR1208	1009	CKD3
chr8:128149926-128150285	chr8	128149926	128150285	17	34	MIR1208	359	HF6
chr18:58450990-58451193	chr18	58450990	58451193	0	21	MIR122	203	CKD9

chr18:58451054-58451408	chr18	58451054	58451408	2	7	MIR122	354	CKD9
chr18:58450517-58451547	chr18	58450517	58451547	24	6	MIR122	1030	CKD12
chr3:184241188-184241533	chr3	184241188	184241533	6	3	MIR1224	345	CKD8
chr3:184241382-184241776	chr3	184241382	184241776	10	8	MIR1224	394	CKD10
chr3:47849468-47849658	chr3	47849468	47849658	0	17	MIR1226	190	CKD20
chr3:47849004-47849696	chr3	47849004	47849696	78	100	MIR1226	692	CKD25
chr5:179797956-179798617	chr5	179797956	179798617	26	82	MIR1229	661	CKD9
chr1:201808097-201808795	chr1	201808097	201808795	20	40	MIR1231	698	CKD8
chr1:201808594-201808943	chr1	201808594	201808943	0	6	MIR1231	349	CKD11
chr1:201808556-201808907	chr1	201808556	201808907	12	73	MIR1231	351	CKD13
chr1:201808556-201808927	chr1	201808556	201808927	15	4	MIR1231	371	CKD13
chr1:201808419-201808963	chr1	201808419	201808963	5	12	MIR1231	544	CKD14
chr8:144400066-144400410	chr8	144400066	144400410	100	91	MIR1234	344	CKD24
chr8:144400082-144400439	chr8	144400082	144400439	4	18	MIR1234	357	HM1
chr19:10552020-10552361	chr19	10552020	10552361	0	7	MIR1238	341	CKD7
chr12:12111455-12112164	chr12	12111455	12112164	34	20	MIR1244-4	709	CKD9
chr12:12111844-12112200	chr12	12111844	12112200	6	8	MIR1244-4	356	CKD18
chr12:12110009-12112489	chr12	12110009	12112489	22	69	MIR1244-4	2480	HM1
chr3:186786560-186787905	chr3	186786560	186787905	4	4	MIR1248	1345	CKD3
chr3:186786575-186788088	chr3	186786575	186788088	2	14	MIR1248	1513	CKD16
chr22:45200873-45201239	chr22	45200873	45201239	1	5	MIR1249	366	CKD4
chr22:45200743-45201083	chr22	45200743	45201083	10	15	MIR1249	340	CKD13
chr22:45200879-45201600	chr22	45200879	45201600	36	73	MIR1249	721	HM1
chr12:79419142-79419557	chr12	79419142	79419557	18	18	MIR1252	415	CKD9
chr12:79419229-79419566	chr12	79419229	79419566	7	54	MIR1252	337	CKD16
chr17:2747639-2748729	chr17	2747639	2748729	9	10	MIR1253	1090	CKD13
chr4:101330110-101330505	chr4	101330110	101330505	9	5	MIR1255A	395	CKD3
chr4:101330272-101330462	chr4	101330272	101330462	0	18	MIR1255A	190	HM1
chr4:36425858-36426445	chr4	36425858	36426445	0	5	MIR1255B1	587	CKD21
chr1:167998626-167999302	chr1	167998626	167999302	14	5	MIR1255B2	676	CKD15
chr1:20988241-20988629	chr1	20988241	20988629	5	4	MIR1256	388	CKD8
chr1:20988298-20988638	chr1	20988298	20988638	4	10	MIR1256	340	CKD8
chr1:20988180-20988555	chr1	20988180	20988555	18	30	MIR1256	375	CKD13
chr1:20988237-20988888	chr1	20988237	20988888	2	5	MIR1256	651	CKD21
chr1:20988244-20988457	chr1	20988244	20988457	0	11	MIR1256	213	CKD25
chr1:20988166-20988508	chr1	20988166	20988508	48	147	MIR1256	342	HF3
chr2:179860530-179862110	chr2	179860530	179862110	6	28	MIR1258	1580	CKD25
chr2:179860805-179861408	chr2	179860805	179861408	5	14	MIR1258	603	HM6
chr11:122099648-122100175	chr11	122099648	122100175	4	6	MIR125B1	527	CKD8
chr11:122099754-122100125	chr11	122099754	122100125	2	13	MIR125B1	371	CKD14
chr14:77265946-77266724	chr14	77265946	77266724	100	214	MIR1260A	778	CKD9
chr11:96340966-96341780	chr11	96340966	96341780	116	105	MIR1260B	814	HM19
chr11:90867057-90869205	chr11	90867057	90869205	15	10	MIR1261	2148	CKD3
chr11:90868794-90869362	chr11	90868794	90869362	1	40	MIR1261	568	CKD16
chr1:68183084-68183674	chr1	68183084	68183674	16	5	MIR1262	590	CKD13
chr1:68183333-68183740	chr1	68183333	68183740	0	4	MIR1262	407	HM6
chr10:14435088-14437958	chr10	14435088	14437958	0	13	MIR1265	2870	CKD3
chr10:14436171-14436711	chr10	14436171	14436711	4	6	MIR1265	540	CKD8
chr10:14435110-14438843	chr10	14435110	14438843	3	15	MIR1265	3733	CKD16
chr15:22225006-22225790	chr15	22225006	22225790	3	12	MIR1268A	784	HM19
chr17:80098531-80099794	chr17	80098531	80099794	10	15	MIR1268B	1263	CKD3
chr17:12916816-12917542	chr17	12916816	12917542	21	41	MIR1269B	726	CKD3
chr19:20398987-20399935	chr19	20398987	20399935	28	48	MIR1270	948	CKD25
chr5:176366729-176368086	chr5	176366729	176368086	10	18	MIR1271	1357	HM5
chr5:176367160-176368054	chr5	176367160	176368054	17	31	MIR1271	894	HM11
chr15:64761957-64762795	chr15	64761957	64762795	2	5	MIR1272	838	CKD3
chr15:64762169-64762533	chr15	64762169	64762533	0	6	MIR1272	364	CKD8
chr6:154853351-154853666	chr6	154853351	154853666	15	45	MIR1273C	315	CKD3
chr6:154853296-154853647	chr6	154853296	154853647	9	13	MIR1273C	351	CKD4
chr6:154853296-154853647	chr6	154853296	154853647	2	7	MIR1273C	351	CKD12
chr6:154853207-154853777	chr6	154853207	154853777	20	45	MIR1273C	570	CKD22
chr16:24202947-24203303	chr16	24202947	24203303	1	6	MIR1273H	356	CKD4
chr16:24202979-24203533	chr16	24202979	24203533	12	14	MIR1273H	554	CKD9
chr16:24202948-24203677	chr16	24202948	24203677	39	24	MIR1273H	729	CKD16
chr6:33999857-34000277	chr6	33999857	34000277	12	8	MIR1275	420	CKD4
chr6:33999656-34000450	chr6	33999656	34000450	18	18	MIR1275	794	CKD8
chr6:33999869-34000451	chr6	33999869	34000451	108	101	MIR1275	582	HM6
chr15:85770360-85770678	chr15	85770360	85770678	0	6	MIR1276	318	CKD6
chr15:85770366-85770701	chr15	85770366	85770701	0	4	MIR1276	335	CKD12
chr15:85770234-85770875	chr15	85770234	85770875	6	10	MIR1276	641	CKD12
chr15:85770362-85771138	chr15	85770362	85771138	48	156	MIR1276	776	HM1
chr1:193135458-193136583	chr1	193135458	193136583	0	4	MIR1278	1125	CKD3
chr1:193136492-193136840	chr1	193136492	193136840	0	15	MIR1278	348	CKD12
chr15:43793476-43794568	chr15	43793476	43794568	10	15	MIR1282	1092	HM9
chr3:35744054-35744749	chr3	35744054	35744749	14	8	MIR128-2	695	CKD6
chr3:35743206-35744575	chr3	35743206	35744575	22	18	MIR128-2	1369	CKD9
chr3:35744342-35744559	chr3	35744342	35744559	0	8	MIR128-2	217	CKD12
chr3:35744381-35744618	chr3	35744381	35744618	0	15	MIR128-2	237	CKD12

chr3:35744112-35744674	chr3	35744112	35744674	4	7	MIR128-2	562	CKD13
chr3:35744205-35744725	chr3	35744205	35744725	0	6	MIR128-2	520	CKD13
chr3:35744474-35744695	chr3	35744474	35744695	0	17	MIR128-2	221	CKD16
chr19:53688432-53688817	chr19	53688432	53688817	0	10	MIR1283-1	385	HM7
chr19:53758007-53758633	chr19	53758007	53758633	9	12	MIR1283-2	626	CKD14
chr3:71541750-71542272	chr3	71541750	71542272	4	13	MIR1284	522	CKD8
chr3:71541779-71542114	chr3	71541779	71542114	0	5	MIR1284	335	CKD21
chr2:70252618-70253347	chr2	70252618	70253347	114	38	MIR1285-2	729	CKD6
chr2:70252585-70253028	chr2	70252585	70253028	22	21	MIR1285-2	443	CKD18
chr2:70252534-70253095	chr2	70252534	70253095	10	24	MIR1285-2	561	CKD22
chr2:70252895-70253109	chr2	70252895	70253109	1	6	MIR1285-2	214	CKD24
chr10:98395013-98395347	chr10	98395013	98395347	10	30	MIR1287	334	HM1
chr17:16281897-16282255	chr17	16281897	16282255	3	13	MIR1288	358	CKD22
chr5:133427278-133428091	chr5	133427278	133428091	27	19	MIR1289-2	813	CKD9
chr1:18897046-18897739	chr1	18897046	18897739	34	43	MIR1290	693	CKD10
chr1:18896836-18897201	chr1	18896836	18897201	4	14	MIR1290	365	CKD12
chr1:18897025-18897247	chr1	18897025	18897247	0	5	MIR1290	222	CKD12
chr1:18896937-18897485	chr1	18896937	18897485	34	56	MIR1290	548	CKD13
chr1:18896883-18897254	chr1	18896883	18897254	0	3	MIR1290	371	CKD14
chr1:18897038-18897256	chr1	18897038	18897256	0	8	MIR1290	218	CKD16
chr12:48654195-48654556	chr12	48654195	48654556	10	23	MIR1291	361	CKD3
chr12:48654332-48655024	chr12	48654332	48655024	10	13	MIR1291	692	CKD3
chr7:128207864-128208197	chr7	128207864	128208197	2	15	MIR129-1	333	HM1
chr20:2652710-2653336	chr20	2652710	2653336	16	12	MIR1292	626	CKD3
chr11:43580992-43581595	chr11	43580992	43581595	4	22	MIR129-2	603	CKD9
chr11:43581301-43582053	chr11	43581301	43582053	8	10	MIR129-2	752	CKD21
chr11:43581287-43581621	chr11	43581287	43581621	0	6	MIR129-2	334	HM13
chr12:50233744-50234267	chr12	50233744	50234267	0	4	MIR1293	523	CKD6
chr12:50234015-50234358	chr12	50234015	50234358	0	5	MIR1293	343	CKD12
chr12:50233767-50234432	chr12	50233767	50234432	9	6	MIR1293	665	CKD20
chr1:171101539-171101896	chr1	171101539	171101896	4	14	MIR1295A	357	CKD20
chr1:171101539-171101896	chr1	171101539	171101896	4	14	MIR1295B	357	CKD20
chr10:63372819-63373164	chr10	63372819	63373164	0	6	MIR1296	345	CKD7
chr10:63372889-63373526	chr10	63372889	63373526	6	12	MIR1296	637	CKD22
chrX:114715093-114715696	chrX	114715093	114715696	6	6	MIR1298	603	HM19
chr9:40928899-40929272	chr9	40928899	40929272	15	25	MIR1299	373	CKD6
chr9:40928926-40929299	chr9	40928926	40929299	14	40	MIR1299	373	CKD18
chr9:40928963-40929299	chr9	40928963	40929299	4	16	MIR1299	336	CKD18
chr9:40928891-40929202	chr9	40928891	40929202	3	7	MIR1299	311	HM13
chr2:25328638-25329315	chr2	25328638	25329315	1826	3910	MIR1301	677	CKD24
chr2:207266870-207269786	chr2	207266870	207269786	28	34	MIR1302-4	2916	HF4
chr20:50613972-50614838	chr20	50613972	50614838	16	6	MIR1302-5	866	CKD21
chr9:97363369-97363713	chr9	97363369	97363713	4	14	MIR1302-8	344	CKD14
chr9:97363248-97364403	chr9	97363248	97364403	15	22	MIR1302-8	1155	CKD16
chr5:154685582-154685925	chr5	154685582	154685925	18	191	MIR1303	343	CKD4
chr11:93733481-93733806	chr11	93733481	93733806	30	111	MIR1304	325	HM6
chr10:103394205-103394575	chr10	103394205	103394575	6	6	MIR1307	370	HM5
chr11:57641097-57641286	chr11	57641097	57641286	1	37	MIR130A	189	CKD2
chr11:57640942-57641453	chr11	57640942	57641453	10	8	MIR130A	511	CKD21
chr11:57641150-57642471	chr11	57641150	57642471	4	12	MIR130A	1321	CKD21
chr11:57641106-57641622	chr11	57641106	57641622	42	46	MIR130A	516	HM9
chr22:21653273-21654276	chr22	21653273	21654276	6	6	MIR130B	1003	CKD21
chrX:85834833-85836949	chrX	85834833	85836949	0	5	MIR1321	2116	HM1
chr19:53671952-53672357	chr19	53671952	53672357	6	6	MIR1323	405	CKD3
chr19:53671297-53672169	chr19	53671297	53672169	28	50	MIR1323	872	CKD8
chr19:53671741-53672190	chr19	53671741	53672190	12	17	MIR1323	449	CKD8
chr19:53671242-53672061	chr19	53671242	53672061	34	26	MIR1323	819	CKD22
chr19:53671708-53672314	chr19	53671708	53672314	99	343	MIR1323	606	HF8
chr19:53671952-53672166	chr19	53671952	53672166	0	6	MIR1323	214	HM13
chr3:75630554-75630948	chr3	75630554	75630948	27	14	MIR1324	394	CKD9
chr18:21825687-21826375	chr18	21825687	21826375	4	14	MIR133A1	688	CKD4
chr20:62564896-62565862	chr20	62564896	62565862	3	10	MIR133A2	966	CKD8
chr20:62564805-62565181	chr20	62564805	62565181	11	28	MIR133A2	376	HM19
chr11:34941594-34942083	chr11	34941594	34942083	4	10	MIR1343	489	CKD6
chr11:34941792-34942455	chr11	34941792	34942455	16	16	MIR1343	663	HM1
chr11:34941631-34942754	chr11	34941631	34942754	23	55	MIR1343	1123	HM9
chr14:100884077-100885212	chr14	100884077	100885212	2	6	MIR136	1135	CKD21
chr3:44114187-44116142	chr3	44114187	44116142	4	12	MIR138-1	1955	CKD25
chr16:56858450-56859080	chr16	56858450	56859080	13	40	MIR138-2	630	CKD12
chr16:56858443-56858795	chr16	56858443	56858795	21	119	MIR138-2	352	HM1
chr16:69932879-69933452	chr16	69932879	69933452	3	13	MIR140	573	CKD3
chr16:69933007-69933329	chr16	69933007	69933329	3	23	MIR140	322	CKD20
chr16:69932916-69933430	chr16	69932916	69933430	6	13	MIR140	514	CKD25
chr5:149428380-149429448	chr5	149428380	149429448	6	6	MIR143	1068	CKD21
chr5:149430634-149430983	chr5	149430634	149430983	48	170	MIR145	349	CKD6
chr5:149430501-149430892	chr5	149430501	149430892	8	17	MIR145	391	CKD13
chrX:63785953-63786301	chrX	63785953	63786301	2	25	MIR1468	348	CKD20
chr15:96333250-96333468	chr15	96333250	96333468	9	116	MIR1469	218	CKD20

chr5:160484795-160485562	chr5	160484795	160485562	40	46	MIR146A	767	CKD3
chr10:102436456-102436772	chr10	102436456	102436772	5	16	MIR146B	316	CKD6
chr10:102436011-102436851	chr10	102436011	102436851	5	4	MIR146B	840	CKD13
chr10:102436091-102436610	chr10	102436091	102436610	0	15	MIR146B	519	CKD20
chr2:231892223-231892423	chr2	231892223	231892423	2	82	MIR1471	200	CKD24
chr2:231891867-231893007	chr2	231891867	231893007	8	24	MIR1471	1140	CKD25
chr9:120244911-120245285	chr9	120244911	120245285	8	18	MIR147A	374	CKD8
chr9:120244860-120246005	chr9	120244860	120246005	7	7	MIR147A	1145	CKD9
chr9:120244834-120245239	chr9	120244834	120245239	8	15	MIR147A	405	HM6
chr7:25949868-25950220	chr7	25949868	25950220	9	9	MIR148A	352	CKD12
chr7:25949812-25950166	chr7	25949812	25950166	3	9	MIR148A	354	CKD20
chr7:25949542-25950178	chr7	25949542	25950178	48	24	MIR148A	636	CKD21
chr12:54337149-54337394	chr12	54337149	54337394	0	31	MIR148B	245	CKD6
chr2:240455304-240456872	chr2	240455304	240456872	6	20	MIR149	1568	CKD13
chr2:240455022-240456140	chr2	240455022	240456140	0	6	MIR149	1118	HM6
chr8:140732259-140733037	chr8	140732259	140733037	10	17	MIR151A	778	CKD3
chr8:140731257-140733291	chr8	140731257	140733291	0	5	MIR151A	2034	CKD21
chr14:100109387-100109919	chr14	100109387	100109919	52	42	MIR151B	532	CKD8
chr14:100109388-100109772	chr14	100109388	100109772	6	7	MIR151B	384	CKD20
chr17:48036995-48037326	chr17	48036995	48037326	0	5	MIR152	331	CKD4
chr17:48036819-48037347	chr17	48036819	48037347	0	7	MIR152	528	CKD16
chr7:157574088-157574869	chr7	157574088	157574869	9	25	MIR153-2	781	CKD3
chr7:157574140-157575090	chr7	157574140	157575090	12	28	MIR153-2	950	CKD12
chr7:157573532-157575703	chr7	157573532	157575703	8	26	MIR153-2	2171	CKD14
chr1:235852198-235854537	chr1	235852198	235854537	0	5	MIR1537	2339	CKD3
chr1:235852677-235854270	chr1	235852677	235854270	24	28	MIR1537	1593	CKD3
chr1:235852791-235854084	chr1	235852791	235854084	48	59	MIR1537	1293	CKD22
chr18:49487354-49487550	chr18	49487354	49487550	1	4	MIR1539	196	CKD22
chr14:101059722-101060060	chr14	101059722	101060060	4	18	MIR154	338	CKD3
chr14:101059474-101059853	chr14	101059474	101059853	18	73	MIR154	379	CKD6
chr14:101059427-101060405	chr14	101059427	101060405	5	6	MIR154	978	CKD13
chr14:101059467-101059853	chr14	101059467	101059853	4	4	MIR154	386	CKD25
chr21:25573762-25574117	chr21	25573762	25574117	2	10	MIR155	355	CKD20
chrX:39837455-39838123	chrX	39837455	39838123	24	66	MIR1587	668	CKD16
chrX:39837513-39837862	chrX	39837513	39837862	0	20	MIR1587	349	CKD16
chrX:39836947-39837902	chrX	39836947	39837902	6	21	MIR1587	955	CKD25
chr13:50049119-50049456	chr13	50049119	50049456	5	8	MIR15A	337	CKD14
chr3:160403855-160404988	chr3	160403855	160404988	12	28	MIR15B	1133	CKD16
chr3:160403855-160404988	chr3	160403855	160404988	12	28	MIR16-2	1133	CKD16
chr1:198858802-198859168	chr1	198858802	198859168	0	14	MIR181A1	366	CKD8
chr1:198858796-198859184	chr1	198858796	198859184	4	6	MIR181A1	388	HM5
chr9:124692062-124692624	chr9	124692062	124692624	367	409	MIR181A2	562	CKD10
chr9:124692348-124693111	chr9	124692348	124693111	4	8	MIR181A2	763	CKD12
chr1:198858802-198859168	chr1	198858802	198859168	0	14	MIR181B1	366	CKD8
chr1:198858796-198859184	chr1	198858796	198859184	4	6	MIR181B1	388	HM5
chr9:124693192-124693932	chr9	124693192	124693932	9	24	MIR181B2	740	CKD3
chr9:124693479-124693845	chr9	124693479	124693845	12	4	MIR181B2	366	CKD16
chr9:124693619-124693841	chr9	124693619	124693841	0	10	MIR181B2	222	CKD18
chr9:124693579-124693884	chr9	124693579	124693884	8	20	MIR181B2	305	CKD22
chr19:13874788-13875114	chr19	13874788	13875114	1	8	MIR181D	326	CKD12
chr19:13874789-13875185	chr19	13874789	13875185	80	107	MIR181D	396	CKD14
chr7:129770242-129770616	chr7	129770242	129770616	41	71	MIR182	374	HF4
chr20:32237686-32238949	chr20	32237686	32238949	22	82	MIR1825	1263	CKD16
chr12:100189585-100190075	chr12	100189585	100190075	6	14	MIR1827	490	CKD12
chr12:100189647-100190225	chr12	100189647	100190225	18	35	MIR1827	578	HM5
chr15:79209719-79210054	chr15	79209719	79210054	12	30	MIR184	335	CKD6
chr22:20032851-20033267	chr22	20032851	20033267	2	6	MIR185	416	CKD21
chr22:20032859-20033224	chr22	20032859	20033224	0	12	MIR185	365	HM3
chr1:71067471-71068613	chr1	71067471	71068613	28	25	MIR186	1142	CKD3
chr11:61814973-61815998	chr11	61814973	61815998	778	1531	MIR1908	1025	CKD10
chr11:61815159-61815498	chr11	61815159	61815498	17	41	MIR1908	339	CKD24
chr19:1816068-1816401	chr19	1816068	1816401	3	11	MIR1909	333	CKD14
chr1:154193454-154193774	chr1	154193454	154193774	0	18	MIR190B	320	CKD16
chr3:49020264-49020799	chr3	49020264	49020799	854	1068	MIR191	535	HM16
chr16:85741362-85741732	chr16	85741362	85741732	0	6	MIR1910	370	CKD11
chr16:85740818-85742037	chr16	85740818	85742037	8	5	MIR1910	1219	HM6
chrX:114651268-114651640	chrX	114651268	114651640	3	7	MIR1912	372	CKD22
chr6:166509060-166509825	chr6	166509060	166509825	48	18	MIR1913	765	CKD13
chr6:166509061-166509825	chr6	166509061	166509825	33	109	MIR1913	764	CKD13
chr6:166509159-166509528	chr6	166509159	166509528	4	33	MIR1913	369	CKD14
chr20:63940996-63941700	chr20	63940996	63941700	3	8	MIR1914	704	HM1
chr11:64891057-64891388	chr11	64891057	64891388	2	14	MIR192	331	CKD14
chr16:14303866-14304109	chr16	14303866	14304109	0	8	MIR193B	243	CKD4
chr16:14303619-14304057	chr16	14303619	14304057	70	90	MIR193B	438	CKD9
chr16:14303887-14304894	chr16	14303887	14304894	6	5	MIR193B	1007	CKD21
chr1:220117962-220118321	chr1	220117962	220118321	4	7	MIR194-1	359	CKD13
chr17:48632187-48634447	chr17	48632187	48634447	12	12	MIR196A1	2260	CKD3
chr1:109598267-109598988	chr1	109598267	109598988	16	22	MIR197	721	CKD3

chr1:109598763-109599127	chr1	109598763	109599127	6	12	MIR197	364	CKD25
chr16:70030217-70030564	chr16	70030217	70030564	7	15	MIR1972-2	347	CKD8
chr16:70029515-70030593	chr16	70029515	70030593	3	24	MIR1972-2	1078	CKD13
chr16:70030128-70030458	chr16	70030128	70030458	2	6	MIR1972-2	330	CKD20
chr1:26554312-26554689	chr1	26554312	26554689	2	8	MIR1976	377	CKD16
chr1:26554312-26554638	chr1	26554312	26554638	3	9	MIR1976	326	CKD18
chr3:120395189-120395946	chr3	120395189	120395946	0	6	MIR198	757	CKD8
chr3:120395540-120396159	chr3	120395540	120396159	8	22	MIR198	619	CKD16
chr3:120395470-120396039	chr3	120395470	120396039	0	4	MIR198	569	CKD21
chr3:120395647-120395994	chr3	120395647	120395994	8	12	MIR198	347	HM19
chr19:10817314-10817536	chr19	10817314	10817536	6	47	MIR199A1	222	CKD13
chr19:10817275-10817611	chr19	10817275	10817611	9	60	MIR199A1	336	CKD20
chr19:10817251-10817607	chr19	10817251	10817607	10	30	MIR199A1	356	HM1
chr1:172144324-172144928	chr1	172144324	172144928	2	23	MIR199A2	604	CKD4
chr1:172144505-172145255	chr1	172144505	172145255	9	16	MIR199A2	750	HM1
chrX:134168011-134169914	chrX	134168011	134169914	9	5	MIR19B2	1903	CKD13
chrX:134167220-134170034	chrX	134167220	134170034	0	14	MIR19B2	2814	CKD16
chrX:134169464-134169820	chrX	134169464	134169820	7	13	MIR19B2	356	CKD17
chr1:1167087-1167457	chr1	1167087	1167457	1	4	MIR200B	370	CKD8
chr1:1167095-1167441	chr1	1167095	1167441	1	6	MIR200B	346	CKD14
chr12:6963649-6964019	chr12	6963649	6964019	45	27	MIR200C	370	CKD18
chr12:6963578-6963917	chr12	6963578	6963917	6	5	MIR200C	339	CKD22
chr9:70809802-70810142	chr9	70809802	70810142	6	19	MIR204	340	CKD6
chr9:70809747-70810106	chr9	70809747	70810106	0	9	MIR204	359	CKD20
chr1:209432062-209432261	chr1	209432062	209432261	0	4	MIR205	199	CKD6
chr1:209432070-209432438	chr1	209432070	209432438	0	5	MIR205	368	CKD12
chr1:209431812-209432407	chr1	209431812	209432407	96	155	MIR205	595	HM11
chr8:112643043-112643801	chr8	112643043	112643801	6	5	MIR2053	758	CKD12
chr4:125506771-125507650	chr4	125506771	125507650	32	41	MIR2054	879	CKD4
chr4:125507008-125507381	chr4	125507008	125507381	2	5	MIR2054	373	CKD7
chr4:125507166-125507527	chr4	125507166	125507527	3	8	MIR2054	361	CKD16
chr6:52144314-52144520	chr6	52144314	52144520	0	30	MIR206	206	CKD3
chr6:52144349-52144549	chr6	52144349	52144549	9	26	MIR206	200	CKD20
chr6:52144087-52144981	chr6	52144087	52144981	8	6	MIR206	894	CKD25
chr14:23387836-23391882	chr14	23387836	23391882	3	9	MIR208A	4046	CKD16
chr14:23417983-23418174	chr14	23417983	23418174	28	62	MIR208B	191	CKD12
chr14:23417983-23418174	chr14	23417983	23418174	24	123	MIR208B	191	CKD13
chr14:23417983-23418174	chr14	23417983	23418174	37	129	MIR208B	191	CKD14
chr14:23417983-23418174	chr14	23417983	23418174	145	425	MIR208B	191	CKD22
chr14:23417963-23418322	chr14	23417963	23418322	205	84	MIR208B	359	HF3
chr14:23417985-23418337	chr14	23417985	23418337	18	10	MIR208B	352	HM1
chrX:134168011-134169914	chrX	134168011	134169914	9	5	MIR20B	1903	CKD13
chrX:134167220-134170034	chrX	134167220	134170034	0	14	MIR20B	2814	CKD16
chr17:59840200-59841347	chr17	59840200	59841347	8	20	MIR21	1147	CKD13
chr10:114173635-114174207	chr10	114173635	114174207	5	8	MIR2110	572	CKD4
chr10:114173999-114174221	chr10	114173999	114174221	2	6	MIR2110	222	CKD6
chrX:150227723-150228083	chrX	150227723	150228083	4	21	MIR2114	360	HF4
chrX:150227854-150228609	chrX	150227854	150228609	12	6	MIR2114	755	HF5
chr15:59170987-59171491	chr15	59170987	59171491	12	12	MIR2116	504	CKD12
chr15:59170965-59171702	chr15	59170965	59171702	36	88	MIR2116	737	HM5
chr17:43444640-43445187	chr17	43444640	43445187	30	35	MIR2117	547	CKD6
chr17:43444782-43445187	chr17	43444782	43445187	12	6	MIR2117	405	CKD21
chr1:220117842-220118196	chr1	220117842	220118196	6	8	MIR215	354	CKD14
chr2:55988798-55991039	chr2	55988798	55991039	5	12	MIR216A	2241	CKD3
chr2:55988941-55989288	chr2	55988941	55989288	0	42	MIR216A	347	CKD20
chr2:55988609-55989283	chr2	55988609	55989283	4	13	MIR216A	674	CKD25
chr2:56000698-56001490	chr2	56000698	56001490	3	6	MIR216B	792	CKD13
chr4:20528098-20528740	chr4	20528098	20528740	24	9	MIR218-1	642	CKD13
chr5:168767616-168768655	chr5	168767616	168768655	54	14	MIR218-2	1039	HM1
chr5:168767617-168768655	chr5	168767617	168768655	36	88	MIR218-2	1038	HM1
chr9:128392245-128392740	chr9	128392245	128392740	20	33	MIR219A2	495	CKD8
chr9:128392245-128392740	chr9	128392245	128392740	20	33	MIR219B	495	CKD8
chrX:45746019-45746386	chrX	45746019	45746386	0	20	MIR221	367	CKD4
chrX:45746019-45746575	chrX	45746019	45746575	3	5	MIR221	556	CKD13
chrX:66017881-66019339	chrX	66017881	66019339	0	4	MIR223	1458	CKD25
chrX:151958566-151958920	chrX	151958566	151958920	10	25	MIR224	354	CKD14
chr13:24162367-24162722	chr13	24162367	24162722	2	8	MIR2276	355	CKD13
chr13:24162317-24162674	chr13	24162317	24162674	13	25	MIR2276	357	HM16
chr9:94809912-94810266	chr9	94809912	94810266	2	5	MIR2278	354	CKD4
chr9:94809913-94810126	chr9	94809913	94810126	0	10	MIR2278	213	CKD10
chr9:94809639-94810159	chr9	94809639	94810159	0	4	MIR2278	520	CKD13
chr9:94809821-94810125	chr9	94809821	94810125	4	8	MIR2278	304	CKD16
chr9:94809850-94810466	chr9	94809850	94810466	4	4	MIR2278	616	CKD16
chr9:94809940-94810297	chr9	94809940	94810297	0	4	MIR2278	357	CKD25
chr2:207109909-207110494	chr2	207109909	207110494	21	20	MIR2355	585	CKD13
chr2:207108994-207110112	chr2	207108994	207110112	0	4	MIR2355	1118	CKD21
chr2:207109756-207110602	chr2	207109756	207110602	2	15	MIR2355	846	CKD25
chr2:207109800-207110362	chr2	207109800	207110362	28	17	MIR2355	562	HF1

chr2:207109919-207111148	chr2	207109919	207111148	3	9	MIR2355	1229	HM1
chr19:13835692-13837024	chr19	13835692	13837024	30	27	MIR23A	1332	CKD25
chr19:13835789-13836754	chr19	13835789	13836754	21	18	MIR23A	965	CKD25
chr9:95084978-95085329	chr9	95084978	95085329	0	6	MIR23B	351	CKD18
chr9:95085055-95086927	chr9	95085055	95086927	70	96	MIR23B	1872	HM1
chrX:20016622-20017318	chrX	20016622	20017318	4	13	MIR23C	696	CKD3
chr9:95085055-95086927	chr9	95085055	95086927	70	96	MIR24-1	1872	HM1
chr19:13835692-13837024	chr19	13835692	13837024	30	27	MIR24-2	1332	CKD25
chr19:13835789-13836754	chr19	13835789	13836754	21	18	MIR24-2	965	CKD25
chr19:13835970-13836362	chr19	13835970	13836362	2	9	MIR24-2	392	CKD25
chr19:13836086-13836600	chr19	13836086	13836600	3	14	MIR24-2	514	HF5
chr2:239351535-239352254	chr2	239351535	239352254	15	25	MIR2467	719	CKD3
chr2:239351520-239351842	chr2	239351520	239351842	0	10	MIR2467	322	CKD22
chr7:100092993-100093656	chr7	100092993	100093656	23	14	MIR25	663	CKD12
chr7:100093428-100093673	chr7	100093428	100093673	2	27	MIR25	245	CKD18
chr7:100093375-100093715	chr7	100093375	100093715	36	78	MIR25	340	HM5
chr13:101966862-101968160	chr13	101966862	101968160	3	4	MIR2681	1298	CKD3
chr13:101966904-101968813	chr13	101966904	101968813	2	4	MIR2681	1909	CKD4
chr1:98045065-98045624	chr1	98045065	98045624	0	4	MIR2682	559	CKD3
chr1:98045050-98045472	chr1	98045050	98045472	52	52	MIR2682	422	CKD20
chr1:98045175-98045446	chr1	98045175	98045446	2	16	MIR2682	271	HM19
chr12:57824240-57824793	chr12	57824240	57824793	19	44	MIR26A2	553	CKD9
chr2:218402444-218402795	chr2	218402444	218402795	150	292	MIR26B	351	CKD17
chr19:13835692-13837024	chr19	13835692	13837024	30	27	MIR27A	1332	CKD25
chr19:13835789-13836754	chr19	13835789	13836754	21	18	MIR27A	965	CKD25
chr19:13836086-13836600	chr19	13836086	13836600	3	14	MIR27A	514	HF5
chr9:95085385-95085749	chr9	95085385	95085749	138	136	MIR27B	364	CKD9
chr9:95085282-95085651	chr9	95085282	95085651	371	416	MIR27B	369	CKD14
chr9:95085055-95086927	chr9	95085055	95086927	70	96	MIR27B	1872	HM1
chr3:188687611-188689553	chr3	188687611	188689553	22	28	MIR28	1942	CKD15
chr20:58817772-58818545	chr20	58817772	58818545	12	14	MIR298	773	CKD3
chr20:58817861-58818618	chr20	58817861	58818618	70	115	MIR298	757	CKD4
chr20:58818186-58818587	chr20	58818186	58818587	16	30	MIR298	401	HM6
chr14:101023660-101024017	chr14	101023660	101024017	5	12	MIR299	357	HM5
chr1:207801599-207802625	chr1	207801599	207802625	8	9	MIR29B2	1026	CKD21
chr1:207802161-207802530	chr1	207802161	207802530	4	6	MIR29B2	369	CKD21
chr1:207802360-207802529	chr1	207802360	207802529	0	12	MIR29B2	169	CKD22
chr1:207801669-207802014	chr1	207801669	207802014	0	20	MIR29C	345	CKD8
chr1:207801599-207802625	chr1	207801599	207802625	8	9	MIR29C	1026	CKD21
chr14:101041107-101041478	chr14	101041107	101041478	0	12	MIR300	371	CKD12
chr14:101041264-101041632	chr14	101041264	101041632	6	9	MIR300	368	CKD13
chr14:101041340-101042560	chr14	101041340	101042560	12	7	MIR300	1220	CKD21
chr14:101040927-101041702	chr14	101040927	101041702	18	8	MIR300	775	HF5
chr4:112648034-112648359	chr4	112648034	112648359	0	6	MIR302A	325	CKD9
chr4:112648023-112648788	chr4	112648023	112648788	15	22	MIR302A	765	CKD13
chr4:112648012-112648395	chr4	112648012	112648395	4	6	MIR302A	383	CKD22
chr4:112648023-112648788	chr4	112648023	112648788	15	22	MIR302B	765	CKD13
chr4:112648279-112648664	chr4	112648279	112648664	0	13	MIR302B	385	CKD21
chr4:112648023-112648788	chr4	112648023	112648788	15	22	MIR302C	765	CKD13
chr4:112648279-112648664	chr4	112648279	112648664	0	13	MIR302C	385	CKD21
chr4:112647864-112648202	chr4	112647864	112648202	0	10	MIR302D	338	CKD13
chr11:7234279-7234875	chr11	7234279	7234875	4	6	MIR302E	596	CKD12
chr11:7234688-7235705	chr11	7234688	7235705	3	4	MIR302E	1017	CKD21
chr18:30298809-30299193	chr18	30298809	30299193	0	8	MIR302F	384	CKD22
chr17:64500695-64501391	chr17	64500695	64501391	12	4	MIR3064	696	CKD13
chr9:95085055-95086927	chr9	95085055	95086927	70	96	MIR3074	1872	HM1
chr1:40757172-40757529	chr1	40757172	40757529	0	15	MIR30C1	357	CKD8
chr1:40757033-40757413	chr1	40757033	40757413	4	18	MIR30C1	380	CKD9
chr1:40754191-40754525	chr1	40754191	40754525	3	21	MIR30E	334	CKD20
chr9:21511980-21512328	chr9	21511980	21512328	0	4	MIR31	348	CKD6
chr9:21511986-21512376	chr9	21511986	21512376	4	6	MIR31	390	HM6
chr1:23044022-23044665	chr1	23044022	23044665	4	16	MIR3115	643	CKD4
chr1:23043905-23044875	chr1	23043905	23044875	3	4	MIR3115	970	CKD21
chr1:66628428-66629126	chr1	66628428	66629126	12	11	MIR3117	698	CKD13
chr1:170151098-170151869	chr1	170151098	170151869	9	14	MIR3119-1	771	CKD8
chr1:170151098-170151869	chr1	170151098	170151869	9	14	MIR3119-2	771	CKD8
chr1:172138535-172138888	chr1	172138535	172138888	4	21	MIR3120	353	CKD9
chr1:241132177-241132368	chr1	241132177	241132368	0	4	MIR3123	191	CKD18
chr1:248826280-248826665	chr1	248826280	248826665	16	27	MIR3124	385	CKD8
chr1:248825962-248826684	chr1	248825962	248826684	23	37	MIR3124	722	CKD9
chr1:248826009-248826750	chr1	248826009	248826750	30	21	MIR3124	741	CKD13
chr1:248826324-248826543	chr1	248826324	248826543	5	82	MIR3124	219	CKD20
chr1:248826092-248826445	chr1	248826092	248826445	0	4	MIR3124	353	CKD22
chr1:248825702-248826614	chr1	248825702	248826614	6	6	MIR3124	912	CKD25
chr1:248826328-248826676	chr1	248826328	248826676	12	18	MIR3124	348	HM1
chr2:12737283-12737621	chr2	12737283	12737621	0	4	MIR3125	338	CKD21
chr2:69103559-69103883	chr2	69103559	69103883	0	4	MIR3126	324	CKD9
chr2:96797989-96798357	chr2	96797989	96798357	5	24	MIR3127	368	CKD4

chr2:96797788-96798456	chr2	96797788	96798456	19	45	MIR3127	668	CKD12
chr2:177255345-177256080	chr2	177255345	177256080	48	129	MIR3128	735	CKD9
chr2:177255677-177256232	chr2	177255677	177256232	6	6	MIR3128	555	CKD13
chr2:206782776-206783411	chr2	206782776	206783411	42	76	MIR3130-1	635	CKD3
chr2:206782776-206783411	chr2	206782776	206783411	42	76	MIR3130-2	635	CKD3
chr2:219058120-219058923	chr2	219058120	219058923	22	27	MIR3131	803	CKD3
chr2:219058168-219058770	chr2	219058168	219058770	57	40	MIR3131	602	CKD16
chr2:219058252-219058924	chr2	219058252	219058924	0	8	MIR3131	672	CKD21
chr2:219549036-219549361	chr2	219549036	219549361	36	94	MIR3132	325	CKD12
chr2:219549012-219549337	chr2	219549012	219549337	13	47	MIR3132	325	HM3
chr2:241477350-241478087	chr2	241477350	241478087	4	6	MIR3133	737	CKD22
chr3:15696766-15698230	chr3	15696766	15698230	9	10	MIR3134	1464	CKD15
chr3:15697101-15697451	chr3	15697101	15697451	2	6	MIR3134	350	CKD16
chr3:20137564-20137764	chr3	20137564	20137764	0	8	MIR3135A	200	CKD12
chr3:69048605-69049643	chr3	69048605	69049643	0	3	MIR3136	1038	CKD21
chr4:143343206-143343580	chr4	143343206	143343580	8	60	MIR3139	374	CKD3
chr4:143342497-143347677	chr4	143342497	143347677	10	23	MIR3139	5180	CKD3
chr4:143343369-143343581	chr4	143343369	143343581	0	74	MIR3139	212	CKD20
chr4:152489223-152489585	chr4	152489223	152489585	2	8	MIR3140	362	HM3
chr5:154595691-154596097	chr5	154595691	154596097	24	41	MIR3141	406	CKD13
chr5:160474260-160475292	chr5	160474260	160475292	4	5	MIR3142	1032	CKD16
chr6:138435150-138435488	chr6	138435150	138435488	2	6	MIR3145	338	CKD3
chr6:138434599-138435299	chr6	138434599	138435299	54	56	MIR3145	700	CKD8
chr7:19705260-19705641	chr7	19705260	19705641	0	5	MIR3146	381	CKD12
chr8:29957194-29957563	chr8	29957194	29957563	1	26	MIR3148	369	HM3
chr8:95072418-95073288	chr8	95072418	95073288	15	7	MIR3150A	870	CKD3
chr8:95072834-95073157	chr8	95072834	95073157	8	60	MIR3150A	323	CKD8
chr8:95072740-95073065	chr8	95072740	95073065	0	10	MIR3150A	325	CKD12
chr8:95072855-95073079	chr8	95072855	95073079	8	20	MIR3150A	224	CKD13
chr8:95072844-95074561	chr8	95072844	95074561	3	6	MIR3150A	1717	CKD25
chr8:95072638-95073028	chr8	95072638	95073028	51	37	MIR3150A	390	HM19
chr8:95072418-95073288	chr8	95072418	95073288	15	7	MIR3150B	870	CKD3
chr8:95072834-95073157	chr8	95072834	95073157	8	60	MIR3150B	323	CKD8
chr8:95072740-95073065	chr8	95072740	95073065	0	10	MIR3150B	325	CKD12
chr8:95072855-95073079	chr8	95072855	95073079	8	20	MIR3150B	224	CKD13
chr8:95072844-95074561	chr8	95072844	95074561	3	6	MIR3150B	1717	CKD25
chr8:95072638-95073028	chr8	95072638	95073028	51	37	MIR3150B	390	HM19
chr9:89312187-89312356	chr9	89312187	89312356	1	10	MIR3153	169	CKD6
chr9:89312014-89312386	chr9	89312014	89312386	4	12	MIR3153	372	CKD24
chr9:128244762-128245139	chr9	128244762	128245139	0	13	MIR3154	377	CKD8
chr10:6151847-6152845	chr10	6151847	6152845	18	32	MIR3155A	998	CKD12
chr10:6151639-6152363	chr10	6151639	6152363	24	35	MIR3155A	724	CKD20
chr10:6152002-6152343	chr10	6152002	6152343	10	16	MIR3155A	341	CKD20
chr10:6151847-6152845	chr10	6151847	6152845	18	32	MIR3155B	998	CKD12
chr10:6151639-6152363	chr10	6151639	6152363	24	35	MIR3155B	724	CKD20
chr10:6152002-6152343	chr10	6152002	6152343	10	16	MIR3155B	341	CKD20
chr10:45163526-45165228	chr10	45163526	45165228	0	6	MIR3156-1	1702	CKD4
chr10:45163787-45164368	chr10	45163787	45164368	0	23	MIR3156-1	581	CKD4
chr11:46451649-46452913	chr11	46451649	46452913	1	15	MIR3160-1	1264	CKD16
chr11:46451649-46452913	chr11	46451649	46452913	1	15	MIR3160-2	1264	CKD16
chr11:48096678-48097042	chr11	48096678	48097042	2	8	MIR3161	364	CKD3
chr11:48096651-48097967	chr11	48096651	48097967	15	38	MIR3161	1316	CKD16
chr11:126988241-126988614	chr11	126988241	126988614	2	14	MIR3167	373	CKD20
chr11:126988272-126988614	chr11	126988272	126988614	20	16	MIR3167	342	CKD21
chr13:61199793-61200989	chr13	61199793	61200989	3	13	MIR3169	1196	CKD16
chr13:61199768-61199979	chr13	61199768	61199979	1	61	MIR3169	211	HM8
chr13:98207975-98208767	chr13	98207975	98208767	0	4	MIR3170	792	CKD21
chr14:95137803-95138043	chr14	95137803	95138043	0	37	MIR3173	240	CKD18
chr14:95137916-95138354	chr14	95137916	95138354	8	6	MIR3173	438	HM19
chr15:90006715-90007151	chr15	90006715	90007151	9	6	MIR3174	436	CKD12
chr15:90006653-90007082	chr15	90006653	90007082	0	6	MIR3174	429	CKD25
chr16:543199-543561	chr16	543199	543561	28	59	MIR3176	362	CKD14
chr16:541754-543446	chr16	541754	543446	24	47	MIR3176	1692	CKD16
chr16:1734799-1735338	chr16	1734799	1735338	3	15	MIR3177	539	CKD3
chr16:1734801-1735125	chr16	1734801	1735125	6	14	MIR3177	324	CKD4
chr16:1734780-1735159	chr16	1734780	1735159	4	18	MIR3177	379	CKD21
chr16:1734800-1735129	chr16	1734800	1735129	3	21	MIR3177	329	HM5
chr17:30116849-30117242	chr17	30116849	30117242	0	4	MIR3184	393	CKD3
chr17:30117017-30117221	chr17	30117017	30117221	0	16	MIR3184	204	CKD12
chr17:30116999-30117181	chr17	30116999	30117181	1	12	MIR3184	182	CKD14
chr17:30116829-30117178	chr17	30116829	30117178	10	18	MIR3184	349	CKD16
chr17:30116848-30117221	chr17	30116848	30117221	18	8	MIR3184	373	CKD22
chr17:30117059-30117462	chr17	30117059	30117462	36	41	MIR3184	403	HM11
chr17:48724034-48724656	chr17	48724034	48724656	14	9	MIR3185	622	CKD3
chr17:48724297-48724713	chr17	48724297	48724713	27	20	MIR3185	416	CKD22
chr17:48724283-48724655	chr17	48724283	48724655	5	10	MIR3185	372	HF5
chr17:81450673-81451193	chr17	81450673	81451193	3	7	MIR3186	520	CKD3
chr19:813161-814137	chr19	813161	814137	40	60	MIR3187	976	CKD25

chr19:18386125-18386782	chr19	18386125	18386782	30	54	MIR3189	657	CKD8
chr20:18470363-18470725	chr20	18470363	18470725	3	10	MIR3192	362	CKD6
chr20:18470370-18470725	chr20	18470370	18470725	1	4	MIR3192	355	CKD8
chr20:18470316-18470695	chr20	18470316	18470695	4	30	MIR3192	379	CKD14
chr20:18470395-18472061	chr20	18470395	18472061	2	14	MIR3192	1666	CKD14
chr20:18470611-18470936	chr20	18470611	18470936	0	45	MIR3192	325	CKD14
chr20:18470543-18470934	chr20	18470543	18470934	4	6	MIR3192	391	HM6
chr20:18470592-18470792	chr20	18470592	18470792	93	308	MIR3192	200	HM7
chr20:18470466-18470801	chr20	18470466	18470801	16	6	MIR3192	335	HM15
chr20:31606990-31607570	chr20	31606990	31607570	18	31	MIR3193	580	CKD16
chr20:51452786-51452992	chr20	51452786	51452992	0	266	MIR3194	206	CKD12
chr20:63238486-63238869	chr20	63238486	63238869	34	25	MIR3196	383	HM19
chr22:17764153-17764549	chr22	17764153	17764549	23	17	MIR3198-1	396	CKD22
chr12:54230512-54231576	chr12	54230512	54231576	2	6	MIR3198-2	1064	CKD3
chr12:54231268-54231606	chr12	54231268	54231606	1	15	MIR3198-2	338	CKD9
chr12:54231374-54231609	chr12	54231374	54231609	0	143	MIR3198-2	235	CKD25
chr22:27920423-27920980	chr22	27920423	27920980	8	8	MIR3199-1	557	CKD13
chr22:27920467-27920790	chr22	27920467	27920790	0	6	MIR3199-1	323	HM1
chr22:27920423-27920980	chr22	27920423	27920980	8	8	MIR3199-2	557	CKD13
chr22:27920467-27920790	chr22	27920467	27920790	0	6	MIR3199-2	323	HM1
chr22:30731133-30731822	chr22	30731133	30731822	52	67	MIR3200	689	CKD3
chr22:30731413-30731757	chr22	30731413	30731757	12	86	MIR3200	344	CKD6
chr22:30731414-30731947	chr22	30731414	30731947	16	19	MIR3200	533	CKD12
chr22:30731413-30731740	chr22	30731413	30731740	10	26	MIR3200	327	CKD13
chr22:30731147-30731756	chr22	30731147	30731756	32	58	MIR3200	609	CKD16
chr22:30731336-30731703	chr22	30731336	30731703	78	87	MIR3200	367	CKD20
chr22:30731485-30731856	chr22	30731485	30731856	0	5	MIR3200	371	CKD21
chr22:30731414-30731969	chr22	30731414	30731969	1	5	MIR3200	555	CKD22
chr22:48274238-48274934	chr22	48274238	48274934	52	106	MIR3201	696	HM11
chrX:153980585-153981275	chrX	153980585	153981275	27	52	MIR3202-1	690	CKD13
chrX:153980585-153981275	chrX	153980585	153981275	27	52	MIR3202-2	690	CKD13
chr1:116671604-116671956	chr1	116671604	116671956	4	9	MIR320B1	352	CKD8
chr1:116671603-116671984	chr1	116671603	116671984	7	15	MIR320B1	381	HM6
chr18:24321336-24321779	chr18	24321336	24321779	6	5	MIR320C2	443	CKD16
chrX:140921626-140926327	chrX	140921626	140926327	1	5	MIR320D2	4701	CKD25
chr14:101025605-101025944	chr14	101025605	101025944	0	12	MIR323A	339	CKD13
chr14:101056047-101056411	chr14	101056047	101056411	8	19	MIR323B	364	CKD4
chr14:101056047-101056636	chr14	101056047	101056636	8	14	MIR323B	589	HM9
chr14:101056114-101056510	chr14	101056114	101056510	8	14	MIR323B	396	HM19
chr17:7223124-7223472	chr17	7223124	7223472	8	14	MIR324	348	CKD9
chr17:7223172-7223488	chr17	7223172	7223488	0	11	MIR324	316	CKD21
chrX:77005313-77006023	chrX	77005313	77006023	3	8	MIR325	710	CKD25
chr11:75334497-75336166	chr11	75334497	75336166	12	15	MIR326	1669	CKD12
chr16:67202008-67202677	chr16	67202008	67202677	21	11	MIR328	669	CKD4
chr16:67202292-67202996	chr16	67202292	67202996	6	33	MIR328	704	CKD4
chr14:101026384-101027104	chr14	101026384	101027104	9	18	MIR329-1	720	CKD21
chr14:101026883-101027762	chr14	101026883	101027762	24	10	MIR329-2	879	CKD3
chr14:101027037-101027382	chr14	101027037	101027382	0	10	MIR329-2	345	HM1
chr19:45638903-45639167	chr19	45638903	45639167	0	6	MIR330	264	CKD4
chr19:45638652-45639340	chr19	45638652	45639340	8	9	MIR330	688	CKD12
chr19:45638504-45639139	chr19	45638504	45639139	51	98	MIR330	635	CKD22
chr19:45638912-45639237	chr19	45638912	45639237	42	148	MIR330	325	HM1
chr12:95308210-95308597	chr12	95308210	95308597	2	28	MIR331	387	CKD6
chr12:95308368-95308713	chr12	95308368	95308713	9	12	MIR331	345	CKD22
chr12:95308317-95308520	chr12	95308317	95308520	1	7	MIR331	203	HM6
chr7:1022896-1023268	chr7	1022896	1023268	19	30	MIR339	372	HM5
chr22:41900899-41901225	chr22	41900899	41901225	3	13	MIR33A	326	CKD6
chr22:41900592-41901132	chr22	41900592	41901132	6	17	MIR33A	540	CKD9
chr17:17813634-17813979	chr17	17813634	17813979	4	19	MIR33B	345	CKD20
chr17:17813357-17814474	chr17	17813357	17814474	14	13	MIR33B	1117	HF5
chr17:17813777-17814119	chr17	17813777	17814119	17	33	MIR33B	342	HM19
chr14:100109387-100109919	chr14	100109387	100109919	52	42	MIR342	532	CKD8
chr14:100109388-100109772	chr14	100109388	100109772	6	7	MIR342	384	CKD20
chr14:100307697-100308089	chr14	100307697	100308089	0	6	MIR345	392	CKD16
chr10:86264574-86264967	chr10	86264574	86264967	32	55	MIR346	393	CKD24
chr1:9151412-9152178	chr1	9151412	9152178	0	10	MIR34A	766	CKD3
chr1:9151618-9152647	chr1	9151618	9152647	4	16	MIR34A	1029	CKD8
chr11:111512274-111513438	chr11	111512274	111513438	9	14	MIR34B	1164	HM1
chr11:111512706-111513109	chr11	111512706	111513109	4	12	MIR34B	403	HM5
chr11:111512272-111513312	chr11	111512272	111513312	25	62	MIR34B	1040	HM6
chr11:111513264-111513602	chr11	111513264	111513602	4	12	MIR34C	338	CKD4
chr11:111513406-111514115	chr11	111513406	111514115	40	69	MIR34C	709	CKD10
chr11:111513364-111513692	chr11	111513364	111513692	0	24	MIR34C	328	CKD21
chr11:111513224-111513781	chr11	111513224	111513781	54	45	MIR34C	557	CKD22
chr11:111513336-111513682	chr11	111513336	111513682	0	10	MIR34C	346	HM1
chr15:88611746-88612079	chr15	88611746	88612079	4	8	MIR3529	333	CKD6
chr18:58450990-58451193	chr18	58450990	58451193	0	21	MIR3591	203	CKD9
chr18:58451054-58451408	chr18	58451054	58451408	2	7	MIR3591	354	CKD9

chr18:58450517-58451547	chr18	58450517	58451547	24	6	MIR3591	1030	CKD12
chr1:33332221-33332563	chr1	33332221	33332563	166	571	MIR3605	342	CKD18
chr1:33332175-33332559	chr1	33332175	33332559	24	29	MIR3605	384	CKD22
chr2:188995287-188995817	chr2	188995287	188995817	0	4	MIR3606	530	CKD25
chr7:98881635-98883150	chr7	98881635	98883150	9	36	MIR3609	1515	CKD13
chr7:98881211-98881883	chr7	98881211	98881883	12	7	MIR3609	672	CKD25
chrX:85903373-85903758	chrX	85903373	85903758	2	16	MIR361	385	CKD14
chr10:35079412-35079806	chr10	35079412	35079806	10	6	MIR3611	394	CKD20
chr10:35079417-35079777	chr10	35079417	35079777	6	8	MIR3611	360	CKD20
chr12:128293932-128294287	chr12	128293932	128294287	0	6	MIR3612	355	CKD3
chr12:128293950-128294747	chr12	128293950	128294747	15	14	MIR3612	797	CKD13
chr12:128293902-128294280	chr12	128293902	128294280	10	8	MIR3612	378	HF4
chr20:47166737-47168110	chr20	47166737	47168110	4	20	MIR3616	1373	CKD25
chr20:47166723-47167065	chr20	47166723	47167065	16	96	MIR3616	342	HM9
chr20:45704991-45705190	chr20	45704991	45705190	13	50	MIR3617	199	CKD9
chr20:45704990-45705382	chr20	45704990	45705382	8	5	MIR3617	392	CKD14
chr22:46091006-46091350	chr22	46091006	46091350	1	8	MIR3619	344	CKD10
chr22:46090813-46091200	chr22	46090813	46091200	2	6	MIR3619	387	CKD18
chr22:46090514-46091163	chr22	46090514	46091163	3	5	MIR3619	649	CKD21
chr22:46090923-46091290	chr22	46090923	46091290	6	7	MIR3619	367	CKD21
chr22:46091028-46091397	chr22	46091028	46091397	12	42	MIR3619	369	HM1
chrX:50008388-50009177	chrX	50008388	50009177	16	35	MIR362	789	CKD16
chr1:228097165-228098656	chr1	228097165	228098656	8	11	MIR3620	1491	CKD16
chrX:134169186-134169564	chrX	134169186	134169564	2	6	MIR363	378	CKD6
chrX:134168011-134169914	chrX	134168011	134169914	9	5	MIR363	1903	CKD13
chrX:134167220-134170034	chrX	134167220	134170034	0	14	MIR363	2814	CKD16
chr20:44407891-44408261	chr20	44407891	44408261	3	7	MIR3646	370	CKD4
chr20:44408050-44408386	chr20	44408050	44408386	4	10	MIR3646	336	CKD4
chr20:44407854-44408219	chr20	44407854	44408219	4	11	MIR3646	365	CKD8
chr20:44407891-44408204	chr20	44407891	44408204	39	172	MIR3646	313	HF1
chr12:1660307-1660697	chr12	1660307	1660697	1	4	MIR3649	390	CKD8
chr12:1660159-1660573	chr12	1660159	1660573	7	17	MIR3649	414	CKD20
chr12:1660211-1660588	chr12	1660211	1660588	0	10	MIR3649	377	CKD25
chr12:1660246-1660631	chr12	1660246	1660631	30	28	MIR3649	385	HM16
chr5:38557480-38558643	chr5	38557480	38558643	4	12	MIR3650	1163	CKD3
chr5:38557408-38557766	chr5	38557408	38557766	0	9	MIR3650	358	CKD9
chr5:38557241-38557659	chr5	38557241	38557659	16	15	MIR3650	418	CKD16
chr5:38557298-38557635	chr5	38557298	38557635	39	138	MIR3650	337	HM11
chr9:92292347-92292680	chr9	92292347	92292680	0	3	MIR3651	333	CKD8
chr9:92292374-92292716	chr9	92292374	92292716	0	9	MIR3651	342	CKD9
chr9:92292254-92292650	chr9	92292254	92292650	14	12	MIR3651	396	CKD25
chr9:92292208-92292597	chr9	92292208	92292597	78	101	MIR3651	389	HM11
chr1:38089163-38089541	chr1	38089163	38089541	15	18	MIR3659	378	CKD9
chr1:38089208-38089542	chr1	38089208	38089542	0	5	MIR3659	334	CKD21
chr1:38088561-38089620	chr1	38088561	38089620	8	11	MIR3659	1059	CKD25
chr1:38088981-38089359	chr1	38088981	38089359	4	16	MIR3659	378	CKD25
chr16:14309166-14309946	chr16	14309166	14309946	50	35	MIR365A	780	CKD4
chr16:14309165-14310163	chr16	14309165	14310163	32	62	MIR365A	998	CKD12
chr16:14309183-14309543	chr16	14309183	14309543	8	20	MIR365A	360	CKD12
chr17:31574900-31575926	chr17	31574900	31575926	9	17	MIR365B	1026	CKD16
chr17:31575229-31575571	chr17	31575229	31575571	1	19	MIR365B	342	CKD25
chr5:90016366-90018672	chr5	90016366	90018672	3	6	MIR3660	2306	CKD16
chr10:117167626-117168009	chr10	117167626	117168009	7	5	MIR3663	383	CKD24
chr10:117167448-117167783	chr10	117167448	117167783	30	92	MIR3663	335	HF8
chr13:77697971-77698195	chr13	77697971	77698195	0	6	MIR3665	224	HM5
chr7:114651450-114653554	chr7	114651450	114653554	2	11	MIR3666	2104	CKD3
chr7:114653247-114653601	chr7	114653247	114653601	26	272	MIR3666	354	CKD22
chr7:114653341-114653740	chr7	114653341	114653740	5	17	MIR3666	399	HM1
chr22:49543190-49543749	chr22	49543190	49543749	6	8	MIR3667	559	CKD3
chr22:49543091-49543482	chr22	49543091	49543482	17	13	MIR3667	391	CKD16
chr22:49543157-49543545	chr22	49543157	49543545	4	29	MIR3667	388	CKD20
chr22:49543311-49543659	chr22	49543311	49543659	2	6	MIR3667	348	CKD21
chr22:49542683-49544442	chr22	49542683	49544442	12	10	MIR3667	1759	CKD25
chr4:112647864-112648202	chr4	112647864	112648202	0	10	MIR367	338	CKD13
chr1:65057361-65058050	chr1	65057361	65058050	21	40	MIR3671	689	HM6
chr16:2270693-2271392	chr16	2270693	2271392	0	5	MIR3677	699	CKD4
chr16:2270405-2270806	chr16	2270405	2270806	12	8	MIR3677	401	CKD9
chr16:2270085-2270883	chr16	2270085	2270883	0	18	MIR3677	798	CKD25
chr2:134126612-134128509	chr2	134126612	134128509	0	9	MIR3679	1897	CKD25
chr2:12198900-12199258	chr2	12198900	12199258	8	47	MIR3681	358	CKD13
chr2:12199000-12199201	chr2	12199000	12199201	8	8	MIR3681	201	CKD22
chr7:7066857-7067146	chr7	7066857	7067146	0	4	MIR3683	289	CKD8
chr7:7066847-7067198	chr7	7066847	7067198	5	8	MIR3683	351	CKD9
chr7:7065895-7067527	chr7	7065895	7067527	15	4	MIR3683	1632	CKD21
chr7:7065925-7067807	chr7	7065925	7067807	0	10	MIR3683	1882	CKD25
chr7:7066949-7067324	chr7	7066949	7067324	3	19	MIR3683	375	HF6
chr7:7066876-7067086	chr7	7066876	7067086	0	28	MIR3683	210	HM5
chr4:98997159-98997492	chr4	98997159	98997492	6	10	MIR3684	333	CKD7

chr12:95309647-95310936	chr12	95309647	95310936	0	10	MIR3685	1289	CKD3
chr4:159128546-159128896	chr4	159128546	159128896	0	15	MIR3688-1	350	CKD16
chr4:159128546-159128896	chr4	159128546	159128896	0	15	MIR3688-2	350	CKD16
chr14:101065340-101065706	chr14	101065340	101065706	22	46	MIR369	366	CKD4
chr14:101065085-101065707	chr14	101065085	101065707	18	6	MIR369	622	CKD21
chr14:101065315-101065688	chr14	101065315	101065688	12	6	MIR369	373	CKD21
chr14:101065500-101065899	chr14	101065500	101065899	3	12	MIR369	399	CKD21
chr14:101065151-101065698	chr14	101065151	101065698	8	5	MIR369	547	CKD22
chr6:5147770-5148472	chr6	5147770	5148472	16	10	MIR3691	702	CKD12
chr6:157529116-157529523	chr6	157529116	157529523	2	7	MIR3692	407	HM6
chr3:16933137-16933457	chr3	16933137	16933457	0	10	MIR3714	320	CKD3
chr3:16933124-16933466	chr3	16933124	16933466	9	14	MIR3714	342	CKD20
chr3:16933121-16933735	chr3	16933121	16933735	22	13	MIR3714	614	CKD21
chr3:16933137-16933535	chr3	16933137	16933535	4	16	MIR3714	398	CKD21
chr3:16933163-16933508	chr3	16933163	16933508	70	154	MIR3714	345	HM7
chr19:53787507-53787879	chr19	53787507	53787879	5	24	MIR371A	372	CKD4
chr19:53786800-53788213	chr19	53786800	53788213	3	6	MIR371A	1413	CKD16
chr19:53787507-53787879	chr19	53787507	53787879	5	24	MIR371B	372	CKD4
chr19:53786800-53788213	chr19	53786800	53788213	3	6	MIR371B	1413	CKD16
chr19:53786800-53788213	chr19	53786800	53788213	3	6	MIR372	1413	CKD16
chr19:53787790-53788521	chr19	53787790	53788521	36	108	MIR372	731	CKD20
chr19:53787697-53788043	chr19	53787697	53788043	0	5	MIR372	346	CKD21
chr19:53787680-53788058	chr19	53787680	53788058	7	12	MIR372	378	HF5
chrX:74218523-74219737	chrX	74218523	74219737	3	10	MIR374B	1214	CKD21
chrX:74218523-74219737	chrX	74218523	74219737	3	10	MIR374C	1214	CKD21
chr2:219001543-219001779	chr2	219001543	219001779	7	25	MIR375	236	CKD13
chr14:101040041-101040392	chr14	101040041	101040392	61	242	MIR376A2	351	CKD20
chr14:101039393-101040541	chr14	101039393	101040541	6	13	MIR376A2	1148	CKD21
chr14:101039393-101040541	chr14	101039393	101040541	6	13	MIR376B	1148	CKD21
chr14:101039392-101039813	chr14	101039392	101039813	23	95	MIR376C	421	CKD3
chr14:101039008-101039796	chr14	101039008	101039796	10	15	MIR376C	788	CKD12
chr14:101039393-101040541	chr14	101039393	101040541	6	13	MIR376C	1148	CKD21
chr14:101061536-101062141	chr14	101061536	101062141	22	64	MIR377	605	CKD16
chr5:149732523-149733223	chr5	149732523	149733223	148	221	MIR378A	700	HM3
chr3:10330184-10331196	chr3	10330184	10331196	8	10	MIR378B	1012	CKD21
chr10:130962478-130962726	chr10	130962478	130962726	0	18	MIR378C	248	CKD6
chr10:130962172-130962719	chr10	130962172	130962719	17	28	MIR378C	547	CKD10
chr10:130962465-130962801	chr10	130962465	130962801	31	158	MIR378C	336	CKD22
chr10:130962373-130962809	chr10	130962373	130962809	131	140	MIR378C	436	HM3
chr4:5922900-5923449	chr4	5922900	5923449	18	43	MIR378D1	549	CKD12
chr4:5923085-5923909	chr4	5923085	5923909	2	5	MIR378D1	824	HM1
chr8:93915998-93916574	chr8	93915998	93916574	24	12	MIR378D2	576	CKD3
chr1:23929018-23929779	chr1	23929018	23929779	4	5	MIR378F	761	HM5
chr1:94745714-94746110	chr1	94745714	94746110	0	14	MIR378G	396	CKD3
chr1:94745781-94746441	chr1	94745781	94746441	9	25	MIR378G	660	CKD8
chr1:94745830-94746026	chr1	94745830	94746026	2	13	MIR378G	196	CKD12
chr5:154829283-154829967	chr5	154829283	154829967	1	5	MIR378H	684	CKD13
chr5:154829406-154829619	chr5	154829406	154829619	0	16	MIR378H	213	HF9
chr22:41922862-41923427	chr22	41922862	41923427	16	12	MIR378I	565	CKD12
chr14:101021839-101022570	chr14	101021839	101022570	81	163	MIR379	731	CKD9
chr14:101021979-101022355	chr14	101021979	101022355	14	47	MIR379	376	CKD24
chr14:101021495-101022224	chr14	101021495	101022224	8	9	MIR379	729	CKD25
chr14:101022011-101022380	chr14	101022011	101022380	2	8	MIR379	369	HM19
chr14:101024993-101025719	chr14	101024993	101025719	21	44	MIR380	726	CKD4
chr14:101054024-101054610	chr14	101054024	101054610	16	5	MIR382	586	CKD4
chr14:101054024-101054400	chr14	101054024	101054400	8	44	MIR382	376	CKD6
chr14:101054023-101054400	chr14	101054023	101054400	14	41	MIR382	377	CKD8
chr14:101054042-101054434	chr14	101054042	101054434	0	12	MIR382	392	CKD11
chr14:101054023-101054400	chr14	101054023	101054400	4	17	MIR382	377	CKD12
chr14:101054216-101054612	chr14	101054216	101054612	26	45	MIR382	396	HM13
chr8:14853334-14853535	chr8	14853334	14853535	0	14	MIR383	201	CKD4
chr8:14853279-14853673	chr8	14853279	14853673	20	6	MIR383	394	CKD16
chr8:14852830-14853605	chr8	14852830	14853605	6	18	MIR383	775	CKD25
chr7:151433372-151433708	chr7	151433372	151433708	4	34	MIR3907	336	CKD15
chr7:151433357-151433714	chr7	151433357	151433714	523	1190	MIR3907	357	HM14
chr22:35335615-35335963	chr22	35335615	35335963	5	4	MIR3909	348	CKD20
chr22:35335396-35336211	chr22	35335396	35336211	2	5	MIR3909	815	CKD21
chr9:91635877-91636632	chr9	91635877	91636632	4	20	MIR3910-1	755	CKD13
chr9:91635877-91636632	chr9	91635877	91636632	4	20	MIR3910-2	755	CKD13
chr9:127690529-127690851	chr9	127690529	127690851	0	15	MIR3911	322	HM1
chr9:127690519-127690899	chr9	127690519	127690899	36	60	MIR3911	380	HM9
chrX:32583200-32583771	chrX	32583200	32583771	4	12	MIR3915	571	CKD3
chr1:25905825-25906524	chr1	25905825	25906524	6	3	MIR3917	699	HM5
chr6:158764128-158765162	chr6	158764128	158765162	4	20	MIR3918	1034	CKD3
chr6:158764133-158765161	chr6	158764133	158765161	2	3	MIR3918	1028	CKD3
chr6:158764635-158764820	chr6	158764635	158764820	0	14	MIR3918	185	CKD10
chr6:158764532-158765430	chr6	158764532	158765430	30	22	MIR3918	898	CKD13
chr6:158764496-158765199	chr6	158764496	158765199	6	27	MIR3918	703	CKD25

chr11:101519542-101520236	chr11	101519542	101520236	4	22	MIR3920	694	CKD25
chr3:99963561-99964563	chr3	99963561	99964563	9	27	MIR3921	1002	CKD25
chr6:36622344-36622694	chr6	36622344	36622694	2	6	MIR3925	350	CKD3
chr6:36622252-36622646	chr6	36622252	36622646	4	16	MIR3925	394	CKD20
chr6:36622369-36622713	chr6	36622369	36622713	6	14	MIR3925	344	CKD20
chr8:12727142-12727480	chr8	12727142	12727480	0	9	MIR3926-1	338	CKD3
chr8:12727144-12727860	chr8	12727144	12727860	0	6	MIR3926-1	716	CKD16
chr8:12727142-12727480	chr8	12727142	12727480	0	9	MIR3926-2	338	CKD3
chr8:12727144-12727860	chr8	12727144	12727860	0	6	MIR3926-2	716	CKD16
chr9:109511218-109511872	chr9	109511218	109511872	28	29	MIR3927	654	CKD9
chr9:109511457-109512040	chr9	109511457	109512040	0	14	MIR3927	583	CKD21
chr18:35934005-35934351	chr18	35934005	35934351	0	8	MIR3929	346	CKD13
chr18:35933534-35934830	chr18	35933534	35934830	2	5	MIR3929	1296	CKD21
chr18:35933909-35934271	chr18	35933909	35934271	0	7	MIR3929	362	CKD21
chr16:56245177-56245837	chr16	56245177	56245837	16	14	MIR3935	660	CKD13
chr16:56245245-56245882	chr16	56245245	56245882	12	7	MIR3935	637	CKD13
chr16:56245343-56245732	chr16	56245343	56245732	8	6	MIR3935	389	HM6
chr16:56245419-56246528	chr16	56245419	56246528	45	60	MIR3935	1109	HM16
chrX:39660415-39661321	chrX	39660415	39661321	1	10	MIR3937	906	CKD3
chrX:39661061-39661397	chrX	39661061	39661397	3	15	MIR3937	336	CKD16
chrX:39661127-39661477	chrX	39661127	39661477	4	6	MIR3937	350	CKD20
chrX:39660718-39661357	chrX	39660718	39661357	3	9	MIR3937	639	CKD25
chr3:55852313-55852639	chr3	55852313	55852639	3	18	MIR3938	326	CKD6
chr3:55851954-55852632	chr3	55851954	55852632	0	10	MIR3938	678	CKD21
chr3:55852445-55852656	chr3	55852445	55852656	0	7	MIR3938	211	CKD22
chr3:55852305-55852656	chr3	55852305	55852656	0	4	MIR3938	351	HF4
chr19:6416404-6417048	chr19	6416404	6417048	38	57	MIR3940	644	HM16
chr15:35372008-35372368	chr15	35372008	35372368	0	6	MIR3942	360	CKD8
chr7:43150890-43151308	chr7	43150890	43151308	8	18	MIR3943	418	CKD9
chr7:43150698-43151065	chr7	43150698	43151065	6	31	MIR3943	367	CKD18
chr7:43150868-43151249	chr7	43150868	43151249	28	21	MIR3943	381	HM1
chr10:133371333-133372020	chr10	133371333	133372020	57	72	MIR3944	687	CKD14
chr4:184850352-184851495	chr4	184850352	184851495	8	12	MIR3945	1143	CKD3
chr4:184850795-184851322	chr4	184850795	184851322	0	6	MIR3945	527	CKD3
chr4:184850937-184851664	chr4	184850937	184851664	10	14	MIR3945	727	CKD6
chr4:184850504-184851253	chr4	184850504	184851253	20	10	MIR3945	749	CKD12
chr4:184850987-184851423	chr4	184850987	184851423	7	22	MIR3945	436	CKD22
chr1:17277761-17278150	chr1	17277761	17278150	2	14	MIR3972	389	CKD8
chr1:17277768-17278483	chr1	17277768	17278483	6	13	MIR3972	715	CKD12
chr1:17277481-17278103	chr1	17277481	17278103	52	103	MIR3972	622	CKD22
chr11:36009304-36010322	chr11	36009304	36010322	4	15	MIR3973	1018	CKD22
chr18:5839757-5840965	chr18	5839757	5840965	0	10	MIR3976	1208	CKD21
chr18:5840605-5841046	chr18	5840605	5841046	8	11	MIR3976	441	CKD22
chr5:82839410-82841435	chr5	82839410	82841435	12	15	MIR3977	2025	CKD16
chr5:82839719-82840471	chr5	82839719	82840471	4	4	MIR3977	752	CKD16
chr14:101064932-101065583	chr14	101064932	101065583	3	11	MIR409	651	CKD3
chr14:101064658-101065456	chr14	101064658	101065456	52	58	MIR409	798	CKD6
chr14:101065240-101065563	chr14	101065240	101065563	84	194	MIR409	323	CKD20
chr14:101065085-101065707	chr14	101065085	101065707	18	6	MIR409	622	CKD21
chr14:101065151-101065698	chr14	101065151	101065698	8	5	MIR409	547	CKD22
chr14:101065742-101066416	chr14	101065742	101066416	48	46	MIR410	674	HM19
chr14:101023039-101023792	chr14	101023039	101023792	8	28	MIR411	753	CKD3
chr14:101023282-101023690	chr14	101023282	101023690	9	14	MIR411	408	CKD8
chr14:101023281-101023641	chr14	101023281	101023641	91	271	MIR411	360	CKD24
chr14:101064932-101065583	chr14	101064932	101065583	3	11	MIR412	651	CKD3
chr14:101065340-101065706	chr14	101065340	101065706	22	46	MIR412	366	CKD4
chr14:101065240-101065563	chr14	101065240	101065563	84	194	MIR412	323	CKD20
chr14:101065085-101065707	chr14	101065085	101065707	18	6	MIR412	622	CKD21
chr14:101065315-101065688	chr14	101065315	101065688	12	6	MIR412	373	CKD21
chr14:101065151-101065698	chr14	101065151	101065698	8	5	MIR412	547	CKD22
chr15:63869693-63871199	chr15	63869693	63871199	35	66	MIR422A	1506	CKD9
chr15:63870713-63871039	chr15	63870713	63871039	2	8	MIR422A	326	CKD22
chr15:63870712-63871057	chr15	63870712	63871057	0	6	MIR422A	345	HM1
chr15:63870824-63871199	chr15	63870824	63871199	171	280	MIR422A	375	HM3
chr17:30116849-30117242	chr17	30116849	30117242	0	4	MIR423	393	CKD3
chr17:30117017-30117221	chr17	30117017	30117221	0	16	MIR423	204	CKD12
chr17:30116999-30117181	chr17	30116999	30117181	1	12	MIR423	182	CKD14
chr17:30116829-30117178	chr17	30116829	30117178	10	18	MIR423	349	CKD16
chr17:30116848-30117221	chr17	30116848	30117221	18	8	MIR423	373	CKD22
chr17:30117059-30117462	chr17	30117059	30117462	36	41	MIR423	403	HM11
chrX:134546543-134546790	chrX	134546543	134546790	0	15	MIR424	247	CKD10
chrX:134546539-134546930	chrX	134546539	134546930	5	7	MIR424	391	HM19
chr1:22862876-22864124	chr1	22862876	22864124	1	15	MIR4253	1248	CKD8
chr1:31758179-31758748	chr1	31758179	31758748	55	65	MIR4254	569	CKD8
chr1:37161381-37161753	chr1	37161381	37161753	13	5	MIR4255	372	CKD6
chr1:37161143-37161755	chr1	37161143	37161755	6	4	MIR4255	612	CKD12
chr1:112461076-112462356	chr1	112461076	112462356	54	12	MIR4256	1280	CKD3
chr1:112461559-112462281	chr1	112461559	112462281	27	48	MIR4256	722	CKD3

chr1:112460466-112462377	chr1	112460466	112462377	7	23	MIR4256	1911	CKD4
chr1:112461537-112461905	chr1	112461537	112461905	0	20	MIR4256	368	HM1
chr1:112461545-112461905	chr1	112461545	112461905	4	15	MIR4256	360	HM3
chr1:150551630-150552020	chr1	150551630	150552020	8	6	MIR4257	390	CKD12
chr1:150551783-150552141	chr1	150551783	150552141	42	29	MIR4257	358	HM11
chr1:154975692-154976603	chr1	154975692	154976603	10	8	MIR4258	911	CKD13
chr1:209623207-209623541	chr1	209623207	209623541	6	9	MIR4260	334	CKD9
chr1:209623411-209623801	chr1	209623411	209623801	3	7	MIR4260	390	CKD16
chr2:10192244-10192863	chr2	10192244	10192863	9	4	MIR4261	619	CKD12
chr2:10192311-10192696	chr2	10192311	10192696	18	65	MIR4261	385	HM19
chr2:27996050-27996957	chr2	27996050	27996957	3	16	MIR4263	907	CKD4
chr2:27996241-27996610	chr2	27996241	27996610	0	4	MIR4263	369	CKD21
chr2:79649066-79649658	chr2	79649066	79649658	4	9	MIR4264	592	CKD8
chr2:109141353-109141717	chr2	109141353	109141717	0	7	MIR4265	364	CKD21
chr2:109141109-109141689	chr2	109141109	109141689	10	41	MIR4265	580	CKD25
chr2:109141440-109141662	chr2	109141440	109141662	12	32	MIR4265	222	HM1
chr2:219906479-219906852	chr2	219906479	219906852	2	4	MIR4268	373	CKD13
chr2:219906368-219906745	chr2	219906368	219906745	4	47	MIR4268	377	CKD14
chr2:219906353-219906724	chr2	219906353	219906724	6	8	MIR4268	371	CKD22
chr2:239305105-239305695	chr2	239305105	239305695	26	51	MIR4269	590	CKD8
chr2:239305012-239305701	chr2	239305012	239305701	6	21	MIR4269	689	CKD13
chr3:49274005-49275306	chr3	49274005	49275306	3	11	MIR4271	1301	CKD25
chr3:67225220-67225644	chr3	67225220	67225644	4	6	MIR4272	424	CKD8
chr3:67224931-67225642	chr3	67224931	67225642	6	22	MIR4272	711	CKD16
chr4:7459209-7460277	chr4	7459209	7460277	100	18	MIR4274	1068	CKD8
chr4:7459487-7460184	chr4	7459487	7460184	14	26	MIR4274	697	HM1
chr4:28819540-28819984	chr4	28819540	28819984	9	6	MIR4275	444	CKD21
chr5:1708662-1709040	chr5	1708662	1709040	11	37	MIR4277	378	HM5
chr5:6827641-6827987	chr5	6827641	6827987	0	6	MIR4278	346	CKD3
chr5:6827552-6827921	chr5	6827552	6827921	4	10	MIR4278	369	CKD4
chr5:6827775-6828159	chr5	6827775	6828159	0	6	MIR4278	384	CKD4
chr5:6827552-6827921	chr5	6827552	6827921	8	16	MIR4278	369	CKD6
chr5:6826886-6828055	chr5	6826886	6828055	8	6	MIR4278	1169	CKD16
chr5:6827779-6829108	chr5	6827779	6829108	40	48	MIR4278	1329	CKD16
chr5:6827283-6828030	chr5	6827283	6828030	18	14	MIR4278	747	CKD25
chr5:31935764-31936751	chr5	31935764	31936751	2	9	MIR4279	987	CKD9
chr5:31935909-31936259	chr5	31935909	31936259	1	10	MIR4279	350	CKD13
chr5:31935972-31936218	chr5	31935972	31936218	0	8	MIR4279	246	CKD18
chr5:31935938-31936344	chr5	31935938	31936344	11	12	MIR4279	406	HM19
chr5:87113800-87116375	chr5	87113800	87116375	12	22	MIR4280	2575	CKD3
chr5:87114576-87115157	chr5	87114576	87115157	8	40	MIR4280	581	CKD8
chr5:87114347-87115070	chr5	87114347	87115070	9	5	MIR4280	723	CKD13
chr5:87114791-87115156	chr5	87114791	87115156	12	4	MIR4280	365	CKD13
chr7:73711047-73711406	chr7	73711047	73711406	10	6	MIR4284	359	CKD3
chr8:27885938-27886834	chr8	27885938	27886834	12	6	MIR4287	896	CKD9
chr8:27885644-27886409	chr8	27885644	27886409	10	13	MIR4287	765	HM19
chr8:28504402-28505660	chr8	28504402	28505660	18	138	MIR4288	1258	CKD13
chr9:88745738-88745952	chr9	88745738	88745952	0	10	MIR4289	214	CKD4
chr1:1168489-1169154	chr1	1168489	1169154	0	4	MIR429	665	CKD21
chr1:1168802-1169669	chr1	1168802	1169669	6	12	MIR429	867	HM1
chr1:1168769-1169824	chr1	1168769	1169824	54	28	MIR429	1055	HM16
chr9:90021123-90023733	chr9	90021123	90023733	12	12	MIR4290	2610	CKD25
chr9:90023333-90023718	chr9	90023333	90023718	2	7	MIR4290	385	HM1
chr9:93819311-93819722	chr9	93819311	93819722	63	41	MIR4291	411	CKD9
chr9:93819215-93819569	chr9	93819215	93819569	42	55	MIR4291	354	CKD16
chr9:93819264-93820032	chr9	93819264	93820032	6	20	MIR4291	768	CKD21
chr9:93819159-93819515	chr9	93819159	93819515	30	71	MIR4291	356	CKD22
chr9:93819257-93819451	chr9	93819257	93819451	0	16	MIR4291	194	CKD24
chr9:93819080-93819429	chr9	93819080	93819429	61	155	MIR4291	349	HM6
chr9:136830935-136831513	chr9	136830935	136831513	8	8	MIR4292	578	CKD3
chr9:136830868-136831267	chr9	136830868	136831267	2	6	MIR4292	399	CKD4
chr9:136830940-136831338	chr9	136830940	136831338	29	32	MIR4292	398	HF3
chr10:14382765-14384016	chr10	14382765	14384016	3	10	MIR4293	1251	CKD3
chr10:14383001-14383369	chr10	14383001	14383369	0	16	MIR4293	368	CKD4
chr10:14382788-14383552	chr10	14382788	14383552	2	33	MIR4293	764	CKD13
chr10:14383001-14383372	chr10	14383001	14383372	10	26	MIR4293	371	CKD24
chr10:14382639-14383699	chr10	14382639	14383699	4	23	MIR4293	1060	CKD25
chr10:14383119-14383454	chr10	14383119	14383454	14	35	MIR4293	335	HM13
chr10:48985402-48985990	chr10	48985402	48985990	2	7	MIR4294	588	HM11
chr10:112633898-112635213	chr10	112633898	112635213	15	40	MIR4295	1315	CKD16
chr10:125032662-125033043	chr10	125032662	125033043	9	5	MIR4296	381	CKD3
chr10:129842824-129843445	chr10	129842824	129843445	78	175	MIR4297	621	CKD22
chr11:1859193-1859551	chr11	1859193	1859551	4	10	MIR4298	358	CKD14
chr11:81890629-81891030	chr11	81890629	81891030	2	8	MIR4300	401	CKD3
chr11:113449747-113450114	chr11	113449747	113450114	11	40	MIR4301	367	CKD3
chr11:113449835-113450210	chr11	113449835	113450210	327	140	MIR4301	375	CKD20
chr11:113449759-113450138	chr11	113449759	113450138	0	11	MIR4301	379	HM5
chr12:97995116-97996084	chr12	97995116	97996084	14	35	MIR4303	968	HM11

chr12:123010585-123010999	chr12	123010585	123010999	4	3	MIR4304	414	CKD14
chr14:26908214-26908786	chr14	26908214	26908786	2	8	MIR4307	572	CKD22
chr14:54877687-54879151	chr14	54877687	54879151	18	5	MIR4308	1464	CKD3
chr14:54877830-54878207	chr14	54877830	54878207	6	5	MIR4308	377	CKD4
chr14:54877610-54878558	chr14	54877610	54878558	8	10	MIR4308	948	CKD16
chr14:54877997-54878353	chr14	54877997	54878353	4	10	MIR4308	356	HF4
chr14:102539527-102540043	chr14	102539527	102540043	44	64	MIR4309	516	CKD8
chr14:100880966-100881335	chr14	100880966	100881335	10	15	MIR431	369	CKD25
chr15:68801807-68802127	chr15	68801807	68802127	0	11	MIR4312	320	CKD9
chr15:68801574-68802559	chr15	68801574	68802559	0	12	MIR4312	985	CKD14
chr15:68801781-68802046	chr15	68801781	68802046	0	9	MIR4312	265	CKD16
chr15:68801771-68801967	chr15	68801771	68801967	0	54	MIR4312	196	CKD18
chr15:68801796-68802001	chr15	68801796	68802001	1	27	MIR4312	205	CKD22
chr15:68801398-68802144	chr15	68801398	68802144	1	8	MIR4312	746	HF5
chr15:68801711-68802045	chr15	68801711	68802045	68	146	MIR4312	334	HM2
chr15:68801646-68802050	chr15	68801646	68802050	6	7	MIR4312	404	HM19
chr17:77396830-77397163	chr17	77396830	77397163	1	42	MIR4316	333	CKD12
chr17:77396877-77397235	chr17	77396877	77397235	0	5	MIR4316	358	HM3
chr17:77396337-77397067	chr17	77396337	77397067	110	256	MIR4316	730	HM5
chr18:6374161-6374932	chr18	6374161	6374932	21	8	MIR4317	771	CKD8
chr18:6374212-6374613	chr18	6374212	6374613	2	9	MIR4317	401	CKD13
chr18:6373984-6374541	chr18	6373984	6374541	33	113	MIR4317	557	CKD24
chr18:44969861-44970220	chr18	44969861	44970220	9	8	MIR4319	359	CKD10
chr18:44969895-44970264	chr18	44969895	44970264	12	22	MIR4319	369	CKD10
chr18:44969705-44970282	chr18	44969705	44970282	0	4	MIR4319	577	CKD25
chr14:100884006-100884780	chr14	100884006	100884780	42	48	MIR432	774	CKD9
chr14:100884077-100885212	chr14	100884077	100885212	2	6	MIR432	1135	CKD21
chr14:100884326-100884714	chr14	100884326	100884714	4	4	MIR432	388	CKD25
chr14:100884208-100884603	chr14	100884208	100884603	0	18	MIR432	395	HM6
chr18:50126351-50126679	chr18	50126351	50126679	7	17	MIR4320	328	CKD8
chr18:50126340-50126730	chr18	50126340	50126730	0	10	MIR4320	390	CKD12
chr18:50126340-50126679	chr18	50126340	50126679	3	7	MIR4320	339	CKD16
chr19:2250010-2250919	chr19	2250010	2250919	4	6	MIR4321	909	CKD12
chr19:10230334-10230957	chr19	10230334	10230957	60	82	MIR4322	623	HM1
chr19:42133384-42133744	chr19	42133384	42133744	3	7	MIR4323	360	CKD14
chr19:42133370-42134026	chr19	42133370	42134026	8	27	MIR4323	656	CKD16
chr19:42133368-42134024	chr19	42133368	42134024	0	8	MIR4323	656	CKD25
chr19:49308710-49308931	chr19	49308710	49308931	0	5	MIR4324	221	CKD16
chr20:57319661-57321920	chr20	57319661	57321920	2	12	MIR4325	2259	CKD16
chr20:63286772-63287135	chr20	63286772	63287135	2	18	MIR4326	363	CKD8
chr20:63286674-63287202	chr20	63286674	63287202	107	159	MIR4326	528	HM19
chr21:30375274-30375609	chr21	30375274	30375609	13	12	MIR4327	335	CKD14
chrX:112780295-112780989	chrX	112780295	112780989	42	168	MIR4329	694	CKD9
chrX:112780610-112780990	chrX	112780610	112780990	8	20	MIR4329	380	HF5
chr14:100881727-100882097	chr14	100881727	100882097	7	19	MIR433	370	CKD6
chr1:51059201-51059971	chr1	51059201	51059971	4	20	MIR4421	770	CKD13
chr1:55225528-55225889	chr1	55225528	55225889	0	8	MIR4422	361	CKD21
chr1:85133384-85134261	chr1	85133384	85134261	2	10	MIR4423	877	CKD16
chr1:178676607-178678861	chr1	178676607	178678861	2	16	MIR4424	2254	CKD16
chr1:25023232-25023698	chr1	25023232	25023698	2	7	MIR4425	466	CKD12
chr1:25023307-25023666	chr1	25023307	25023666	8	10	MIR4425	359	HM1
chr1:233623985-233624654	chr1	233623985	233624654	9	9	MIR4427	669	CKD3
chr1:233624007-233624380	chr1	233624007	233624380	4	14	MIR4427	373	CKD12
chr1:233624079-233624956	chr1	233624079	233624956	6	20	MIR4427	877	CKD21
chr1:233623647-233625191	chr1	233623647	233625191	3	36	MIR4427	1544	CKD25
chr1:237470937-237471324	chr1	237470937	237471324	0	5	MIR4428	387	HM1
chr2:33418217-33418597	chr2	33418217	33418597	21	8	MIR4430	380	CKD14
chr2:52702294-52702916	chr2	52702294	52702916	6	12	MIR4431	622	CKD21
chr2:60387263-60387468	chr2	60387263	60387468	0	5	MIR4432	205	CKD6
chr2:60387097-60387467	chr2	60387097	60387467	4	5	MIR4432	370	CKD13
chr2:64525077-64525688	chr2	64525077	64525688	3	12	MIR4434	611	HF5
chr2:181305176-181305948	chr2	181305176	181305948	9	13	MIR4437	772	CKD13
chr2:181305338-181305724	chr2	181305338	181305724	6	29	MIR4437	386	CKD16
chr2:181305444-181305806	chr2	181305444	181305806	0	18	MIR4437	362	CKD22
chr2:181304927-181305665	chr2	181304927	181305665	4	4	MIR4437	738	CKD25
chr2:181305316-181305723	chr2	181305316	181305723	42	38	MIR4437	407	HM6
chr2:213757449-213758182	chr2	213757449	213758182	5	4	MIR4438	733	HF5
chr2:225009955-225010993	chr2	225009955	225010993	9	19	MIR4439	1038	CKD3
chr2:225010229-225010609	chr2	225010229	225010609	6	4	MIR4439	380	CKD4
chr2:225009928-225010620	chr2	225009928	225010620	30	43	MIR4439	692	CKD14
chr2:225010265-225010645	chr2	225010265	225010645	2	6	MIR4439	380	HF5
chr3:25664305-25665087	chr3	25664305	25665087	18	16	MIR4442	782	CKD12
chr3:25664702-25665260	chr3	25664702	25665260	3	7	MIR4442	558	CKD21
chr3:48195680-48196876	chr3	48195680	48196876	20	58	MIR4443	1196	CKD13
chr3:48196272-48197013	chr3	48196272	48197013	36	30	MIR4443	741	CKD16
chr3:48196240-48196648	chr3	48196240	48196648	0	27	MIR4443	408	CKD21
chr3:113594418-113594982	chr3	113594418	113594982	16	18	MIR4446	564	CKD9
chr3:113594566-113595283	chr3	113594566	113595283	3	22	MIR4446	717	CKD13

chr3:113594847-113595396	chr3	113594847	113595396	45	50	MIR4446	549	CKD22
chr3:116849759-116850433	chr3	116849759	116850433	4	10	MIR4447	674	CKD24
chr3:183886364-183887250	chr3	183886364	183887250	9	6	MIR4448	886	CKD9
chr4:76573509-76573848	chr4	76573509	76573848	0	15	MIR4450	339	HM1
chr4:86541360-86542987	chr4	86541360	86542987	16	10	MIR4452	1627	CKD3
chr4:152536425-152536790	chr4	152536425	152536790	14	13	MIR4453	365	CKD13
chr4:152536133-152536525	chr4	152536133	152536525	2	8	MIR4453	392	HM5
chr5:1309105-1309462	chr5	1309105	1309462	6	13	MIR4457	357	CKD13
chr5:1309136-1309479	chr5	1309136	1309479	104	409	MIR4457	343	HM2
chr5:8460209-8461720	chr5	8460209	8461720	5	6	MIR4458	1511	CKD25
chr5:129396972-129397338	chr5	129396972	129397338	0	6	MIR4460	366	CKD14
chr6:37555143-37555536	chr6	37555143	37555536	11	10	MIR4462	393	CKD10
chr6:75428156-75428687	chr6	75428156	75428687	12	107	MIR4463	531	CKD13
chr6:90312710-90313076	chr6	90312710	90313076	2	6	MIR4464	366	CKD20
chr6:140683648-140684003	chr6	140683648	140684003	4	8	MIR4465	355	CKD7
chr6:140683435-140683929	chr6	140683435	140683929	6	21	MIR4465	494	CKD10
chr6:156779675-156779925	chr6	156779675	156779925	0	12	MIR4466	250	CKD9
chr6:156779675-156780451	chr6	156779675	156780451	21	11	MIR4466	776	CKD12
chr7:102471308-102472315	chr7	102471308	102472315	3	3	MIR4467	1007	CKD21
chr8:42896156-42896444	chr8	42896156	42896444	13	13	MIR4469	288	CKD4
chr8:42896095-42896425	chr8	42896095	42896425	0	22	MIR4469	330	CKD14
chr8:100382604-100382965	chr8	100382604	100382965	3	16	MIR4471	361	CKD20
chr8:142176254-142176601	chr8	142176254	142176601	132	171	MIR4472-1	347	CKD6
chr12:116426955-116429898	chr12	116426955	116429898	14	4	MIR4472-2	2943	CKD15
chr12:116427614-116428695	chr12	116427614	116428695	18	16	MIR4472-2	1081	CKD21
chr9:20410996-20411377	chr9	20410996	20411377	18	50	MIR4473	381	CKD22
chr9:20501464-20503209	chr9	20501464	20503209	16	18	MIR4474	1745	HM16
chr9:36823361-36823724	chr9	36823361	36823724	14	65	MIR4475	363	CKD6
chr9:36823156-36823929	chr9	36823156	36823929	0	4	MIR4475	773	CKD11
chr9:36893214-36893586	chr9	36893214	36893586	12	28	MIR4476	372	CKD10
chr9:122119721-122120415	chr9	122119721	122120415	16	9	MIR4478	694	CKD12
chr9:122120032-122120232	chr9	122120032	122120232	2	8	MIR4478	200	CKD18
chrX:114823441-114823811	chrX	114823441	114823811	19	23	MIR448	370	CKD22
chr10:12578702-12579059	chr10	12578702	12579059	3	13	MIR4480	357	CKD8
chr10:12577747-12579146	chr10	12577747	12579146	0	6	MIR4480	1399	CKD21
chr10:12578722-12579102	chr10	12578722	12579102	11	6	MIR4480	380	HF3
chr10:12653108-12653309	chr10	12653108	12653309	2	10	MIR4481	201	CKD12
chr10:104268243-104268620	chr10	104268243	104268620	0	7	MIR4482	377	CKD3
chr10:104268255-104268898	chr10	104268255	104268898	3	8	MIR4482	643	CKD12
chr10:104267706-104268736	chr10	104267706	104268736	4	35	MIR4482	1030	CKD13
chr10:104267504-104268821	chr10	104267504	104268821	5	4	MIR4482	1317	HM1
chr10:113777789-113778171	chr10	113777789	113778171	5	8	MIR4483	382	CKD8
chr10:113777878-113778270	chr10	113777878	113778270	5	6	MIR4483	392	CKD8
chr10:113777980-113778553	chr10	113777980	113778553	16	44	MIR4483	573	CKD9
chr10:113776364-113778401	chr10	113776364	113778401	22	10	MIR4483	2037	CKD13
chr10:113776927-113778373	chr10	113776927	113778373	0	17	MIR4483	1446	CKD21
chr10:113777879-113778066	chr10	113777879	113778066	0	23	MIR4483	187	HM6
chr11:19575131-19575913	chr11	19575131	19575913	24	39	MIR4486	782	CKD3
chr11:19574884-19576025	chr11	19574884	19576025	12	39	MIR4486	1141	CKD9
chr11:19575228-19575431	chr11	19575228	19575431	0	16	MIR4486	203	HM6
chr11:47400846-47401070	chr11	47400846	47401070	4	10	MIR4487	224	CKD3
chr11:65648961-65649516	chr11	65648961	65649516	44	29	MIR4489	555	CKD3
chr11:65649080-65649443	chr11	65649080	65649443	0	8	MIR4489	363	CKD12
chr11:118910591-118910946	chr11	118910591	118910946	2	4	MIR4492	355	CKD3
chr11:123380461-123381739	chr11	123380461	123381739	23	45	MIR4493	1278	CKD4
chr12:47364177-47364549	chr12	47364177	47364549	30	53	MIR4494	372	HF8
chr12:97938939-97939318	chr12	97938939	97939318	2	8	MIR4495	379	CKD6
chr12:97938841-97939178	chr12	97938841	97939178	0	6	MIR4495	337	CKD16
chr12:97938927-97939935	chr12	97938927	97939935	0	4	MIR4495	1008	CKD21
chr12:108635806-108636200	chr12	108635806	108636200	63	19	MIR4496	394	CKD21
chr12:108635753-108636111	chr12	108635753	108636111	28	124	MIR4496	358	CKD22
chr12:108635806-108636078	chr12	108635806	108636078	0	39	MIR4496	272	HM3
chr12:109833178-109834144	chr12	109833178	109834144	12	24	MIR4497	966	HM11
chr12:120154884-120156186	chr12	120154884	120156186	12	20	MIR4498	1302	HM1
chr12:120155238-120155572	chr12	120155238	120155572	9	52	MIR4498	334	HM5
chr5:55170417-55170840	chr5	55170417	55170840	4	20	MIR449A	423	CKD10
chr5:55170381-55170704	chr5	55170381	55170704	0	4	MIR449A	323	CKD14
chr5:55170417-55170840	chr5	55170417	55170840	4	20	MIR449B	423	CKD10
chr13:114273110-114274125	chr13	114273110	114274125	4	8	MIR4502	1015	CKD3
chr13:114273386-114273965	chr13	114273386	114273965	33	50	MIR4502	579	HM11
chr15:23561795-23562187	chr15	23561795	23562187	4	7	MIR4508	392	CKD3
chr15:23561392-23562398	chr15	23561392	23562398	14	31	MIR4508	1006	CKD8
chr15:23561852-23562226	chr15	23561852	23562226	6	27	MIR4508	374	CKD10
chr15:23561571-23562279	chr15	23561571	23562279	5	32	MIR4508	708	CKD13
chr15:23561865-23562275	chr15	23561865	23562275	2	10	MIR4508	410	CKD14
chr15:23561847-23562225	chr15	23561847	23562225	2	6	MIR4508	378	CKD22
chr15:23561959-23562302	chr15	23561959	23562302	6	7	MIR4508	343	CKD22
chr15:66496950-66497278	chr15	66496950	66497278	2	19	MIR4512	328	CKD22

chr15:66496950-66497324	chr15	66496950	66497324	5	17	MIR4512	374	HM6
chr15:74788517-74788829	chr15	74788517	74788829	78	136	MIR4513	312	CKD12
chr15:74788500-74788832	chr15	74788500	74788832	166	300	MIR4513	332	CKD18
chr15:74788553-74788934	chr15	74788553	74788934	0	7	MIR4513	381	CKD21
chr15:74788478-74789482	chr15	74788478	74789482	6	4	MIR4513	1004	HM5
chr17:28861221-28861555	chr17	28861221	28861555	2	5	MIR451A	334	CKD18
chr17:28861221-28861555	chr17	28861221	28861555	2	5	MIR451B	334	CKD18
chr17:6654651-6655662	chr17	6654651	6655662	4	15	MIR4520-1	1011	CKD3
chr17:6654651-6655662	chr17	6654651	6655662	4	15	MIR4520-2	1011	CKD3
chr17:8186166-8187143	chr17	8186166	8187143	4	7	MIR4521	977	CKD3
chr17:8186792-8187009	chr17	8186792	8187009	1	46	MIR4521	217	CKD10
chr17:8186792-8187009	chr17	8186792	8187009	0	6	MIR4521	217	CKD11
chr17:8186838-8187089	chr17	8186838	8187089	0	5	MIR4521	251	CKD13
chr17:8186911-8187100	chr17	8186911	8187100	0	15	MIR4521	189	CKD16
chr17:8186382-8187077	chr17	8186382	8187077	10	18	MIR4521	695	CKD22
chr17:8186841-8187177	chr17	8186841	8187177	6	17	MIR4521	336	HM16
chr17:29390593-29390802	chr17	29390593	29390802	2	4	MIR4523	209	CKD4
chr17:29390583-29390796	chr17	29390583	29390796	11	68	MIR4523	213	CKD18
chr17:29390504-29390796	chr17	29390504	29390796	209	710	MIR4523	292	HM7
chr17:29390384-29390798	chr17	29390384	29390798	433	308	MIR4523	414	HM19
chr17:69098971-69099723	chr17	69098971	69099723	42	58	MIR4524A	752	CKD24
chr17:69099407-69100418	chr17	69099407	69100418	6	14	MIR4524A	1011	HM1
chr17:69098971-69099723	chr17	69098971	69099723	42	58	MIR4524B	752	CKD24
chr17:69099407-69100418	chr17	69099407	69100418	6	14	MIR4524B	1011	HM1
chr18:13610634-13611488	chr18	13610634	13611488	74	39	MIR4526	854	CKD4
chr18:13611111-13611620	chr18	13611111	13611620	15	38	MIR4526	509	CKD12
chr18:13611086-13611458	chr18	13611086	13611458	6	31	MIR4526	372	CKD14
chr18:13611002-13611371	chr18	13611002	13611371	4	22	MIR4526	369	CKD22
chr18:13610964-13611499	chr18	13610964	13611499	54	92	MIR4526	535	HM1
chr18:47379586-47381893	chr18	47379586	47381893	6	8	MIR4527	2307	CKD3
chr18:47380466-47381100	chr18	47380466	47381100	0	16	MIR4527	634	CKD3
chr18:47379877-47380613	chr18	47379877	47380613	10	4	MIR4527	736	CKD12
chr18:47379926-47381082	chr18	47379926	47381082	36	31	MIR4527	1156	CKD16
chr18:55479207-55479536	chr18	55479207	55479536	0	14	MIR4529	329	CKD3
chr18:55479111-55479463	chr18	55479111	55479463	0	6	MIR4529	352	CKD20
chr19:44653473-44653794	chr19	44653473	44653794	10	49	MIR4531	321	CKD9
chr19:44653477-44653826	chr19	44653477	44653826	0	8	MIR4531	349	CKD21
chr20:60477744-60478332	chr20	60477744	60478332	0	6	MIR4533	588	CKD3
chr20:60478032-60478435	chr20	60478032	60478435	0	5	MIR4533	403	CKD21
chr20:60478082-60478438	chr20	60478082	60478438	36	63	MIR4533	356	HF2
chr20:60478013-60478437	chr20	60478013	60478437	8	6	MIR4533	424	HM19
chr22:37988762-37989121	chr22	37988762	37989121	2	6	MIR4534	359	CKD6
chr22:48780047-48780759	chr22	48780047	48780759	36	115	MIR4535	712	CKD4
chr22:48780037-48780586	chr22	48780037	48780586	6	31	MIR4535	549	CKD21
chr22:48780246-48781237	chr22	48780246	48781237	12	6	MIR4535	991	CKD21
chrX:55451215-55451641	chrX	55451215	55451641	0	4	MIR4536-1	426	CKD25
chrX:55451215-55451641	chrX	55451215	55451641	0	4	MIR4536-2	426	CKD25
chr9:114208857-114209640	chr9	114208857	114209640	54	22	MIR455	783	CKD9
chr1:12191533-12191888	chr1	12191533	12191888	5	17	MIR4632	355	CKD16
chr5:129097563-129097909	chr5	129097563	129097909	12	48	MIR4633	346	CKD16
chr5:129097520-129097896	chr5	129097520	129097896	3	6	MIR4633	376	HF4
chr5:129097564-129097782	chr5	129097564	129097782	0	21	MIR4633	218	HM13
chr5:174751733-174751932	chr5	174751733	174751932	0	32	MIR4634	199	CKD6
chr5:174751597-174751799	chr5	174751597	174751799	0	4	MIR4634	202	CKD14
chr5:174751426-174752252	chr5	174751426	174752252	56	88	MIR4634	826	HM6
chr5:9053230-9054248	chr5	9053230	9054248	24	28	MIR4636	1018	CKD13
chr5:14825771-14826166	chr5	14825771	14826166	10	20	MIR4637	395	CKD9
chr5:14825783-14826472	chr5	14825783	14826472	6	14	MIR4637	689	CKD21
chr5:14825757-14826084	chr5	14825757	14826084	6	40	MIR4637	327	CKD22
chr5:14825804-14826149	chr5	14825804	14826149	3	28	MIR4637	345	CKD25
chr5:181222468-181223077	chr5	181222468	181223077	36	24	MIR4638	609	CKD4
chr5:181222479-181223052	chr5	181222479	181223052	16	24	MIR4638	573	CKD4
chr5:181222497-181223163	chr5	181222497	181223163	4	6	MIR4638	666	CKD21
chr5:181222455-181222640	chr5	181222455	181222640	1	4	MIR4638	185	HM13
chr6:41598502-41598861	chr6	41598502	41598861	1	5	MIR4641	359	CKD12
chr6:41598549-41598875	chr6	41598549	41598875	99	209	MIR4641	326	HF1
chr6:44435487-44435847	chr6	44435487	44435847	0	6	MIR4642	360	CKD9
chr6:91521589-91522732	chr6	91521589	91522732	6	8	MIR4643	1143	CKD3
chr6:2853867-2854543	chr6	2853867	2854543	20	34	MIR4645	676	CKD3
chr6:2854015-2854262	chr6	2854015	2854262	0	13	MIR4645	247	CKD16
chr6:2853939-2854160	chr6	2853939	2854160	2	5	MIR4645	221	HM7
chr6:44254098-44254755	chr6	44254098	44254755	18	54	MIR4647	657	CKD13
chr6:44253847-44254417	chr6	44253847	44254417	9	9	MIR4647	570	CKD16
chr7:44110808-44111194	chr7	44110808	44111194	51	39	MIR4649	386	CKD25
chr7:67113522-67114592	chr7	67113522	67114592	124	216	MIR4650-1	1070	HF1
chr1:162156513-162157401	chr1	162156513	162157401	0	4	MIR4654	888	CKD21
chr1:162156672-162157913	chr1	162156672	162157913	4	3	MIR4654	1241	CKD21
chr1:162156989-162157227	chr1	162156989	162157227	0	15	MIR4654	238	CKD22

chr7:1843915-1844279	chr7	1843915	1844279	8	27	MIR4655	364	CKD12
chr7:1844130-1844525	chr7	1844130	1844525	10	18	MIR4655	395	HM5
chr7:4788222-4788842	chr7	4788222	4788842	24	50	MIR4656	620	HM1
chr7:44880855-44881975	chr7	44880855	44881975	2	7	MIR4657	1120	CKD3
chr7:100156413-100157302	chr7	100156413	100157302	18	11	MIR4658	889	CKD4
chr7:100156472-100156809	chr7	100156472	100156809	2	10	MIR4658	337	CKD14
chr7:100156450-100156810	chr7	100156450	100156810	3	21	MIR4658	360	HM13
chr8:6744929-6745298	chr8	6744929	6745298	9	16	MIR4659A	369	CKD4
chr8:6744929-6745298	chr8	6744929	6745298	9	16	MIR4659B	369	CKD4
chr8:9047612-9048544	chr8	9047612	9048544	32	9	MIR4660	932	CKD9
chr8:9048417-9049069	chr8	9048417	9049069	12	12	MIR4660	652	CKD13
chr8:123214894-123215992	chr8	123214894	123215992	20	16	MIR4663	1098	CKD21
chr8:123215631-123216019	chr8	123215631	123216019	1	8	MIR4663	388	HM5
chr9:35607815-35608462	chr9	35607815	35608462	2	5	MIR4667	647	CKD25
chr9:111931803-111932205	chr9	111931803	111932205	6	8	MIR4668	402	CKD16
chr9:92527539-92528242	chr9	92527539	92528242	12	16	MIR4670	703	CKD3
chr9:92527535-92528278	chr9	92527535	92528278	2	6	MIR4670	743	CKD21
chr9:92527904-92528496	chr9	92527904	92528496	4	6	MIR4670	592	CKD25
chr1:234305076-234306856	chr1	234305076	234306856	20	10	MIR4671	1780	CKD3
chr1:234305947-234306694	chr1	234305947	234306694	18	20	MIR4671	747	CKD12
chr9:127869295-127869677	chr9	127869295	127869677	16	15	MIR4672	382	HM6
chr9:136519566-136519965	chr9	136519566	136519965	92	65	MIR4673	399	HM5
chr9:136519486-136519828	chr9	136519486	136519828	0	15	MIR4673	342	HM19
chr10:20551686-20552180	chr10	20551686	20552180	4	27	MIR4675	494	CKD21
chr10:87503246-87504175	chr10	87503246	87504175	4	10	MIR4678	929	CKD12
chr10:119377833-119378243	chr10	119377833	119378243	0	6	MIR4681	410	CKD12
chr10:119377875-119378255	chr10	119377875	119378255	2	7	MIR4681	380	CKD16
chr10:119377885-119378256	chr10	119377885	119378256	6	21	MIR4681	371	CKD20
chr10:119955797-119959560	chr10	119955797	119959560	6	6	MIR4682	3763	CKD3
chr10:35640980-35641403	chr10	35640980	35641403	5	11	MIR4683	423	HM5
chr1:22719458-22719840	chr1	22719458	22719840	2	13	MIR4684	382	CKD8
chr1:22719494-22719714	chr1	22719494	22719714	2	4	MIR4684	220	CKD13
chr1:22719394-22719739	chr1	22719394	22719739	41	105	MIR4684	345	CKD18
chr1:22719491-22719829	chr1	22719491	22719829	49	125	MIR4684	338	CKD18
chr10:98431060-98431421	chr10	98431060	98431421	4	18	MIR4685	361	CKD22
chr10:98431183-98431750	chr10	98431183	98431750	46	63	MIR4685	567	HF3
chr11:2173044-2173388	chr11	2173044	2173388	0	20	MIR4686	344	CKD12
chr11:3856018-3856696	chr11	3856018	3856696	7	4	MIR4687	678	HF4
chr11:46376353-46376724	chr11	46376353	46376724	4	5	MIR4688	371	HM1
chr1:5862183-5862766	chr1	5862183	5862766	30	68	MIR4689	583	CKD10
chr1:5862279-5863042	chr1	5862279	5863042	0	9	MIR4689	763	CKD12
chr11:72783132-72783833	chr11	72783132	72783833	3	8	MIR4692	701	CKD3
chr11:72783254-72783599	chr11	72783254	72783599	78	118	MIR4692	345	CKD22
chr11:72783378-72783767	chr11	72783378	72783767	12	36	MIR4692	389	HM1
chr11:103849537-103850647	chr11	103849537	103850647	4	11	MIR4693	1110	CKD11
chr11:19759740-19761254	chr11	19759740	19761254	6	16	MIR4694	1514	CKD12
chr11:19759953-19760668	chr11	19759953	19760668	8	14	MIR4694	715	CKD16
chr1:18882949-18884272	chr1	18882949	18884272	3	31	MIR4695	1323	CKD13
chr11:133898418-133898629	chr11	133898418	133898629	10	34	MIR4697	211	CKD13
chr11:133898485-133898664	chr11	133898485	133898664	0	4	MIR4697	179	CKD13
chr11:133898418-133898763	chr11	133898418	133898763	10	57	MIR4697	345	CKD18
chr11:133898047-133898677	chr11	133898047	133898677	4	10	MIR4697	630	CKD21
chr12:120723160-120723352	chr12	120723160	120723352	3	9	MIR4700	192	CKD18
chr12:48771607-48772733	chr12	48771607	48772733	38	48	MIR4701	1126	CKD16
chr13:66218150-66218697	chr13	66218150	66218697	4	4	MIR4704	547	CKD14
chr13:66218188-66218591	chr13	66218188	66218591	0	8	MIR4704	403	CKD14
chr13:66218111-66218705	chr13	66218111	66218705	15	21	MIR4704	594	HM11
chr13:102045847-102046245	chr13	102045847	102046245	61	111	MIR4705	398	CKD4
chr13:102045688-102046013	chr13	102045688	102046013	0	20	MIR4705	325	CKD13
chr14:65044484-65044883	chr14	65044484	65044883	3	8	MIR4706	399	CKD12
chr14:22956581-22957266	chr14	22956581	22957266	10	70	MIR4707	685	CKD8
chr14:65334382-65335363	chr14	65334382	65335363	2	18	MIR4708	981	CKD12
chr14:65335017-65335364	chr14	65335017	65335364	6	23	MIR4708	347	CKD20
chr14:65334841-65335186	chr14	65334841	65335186	8	17	MIR4708	345	CKD22
chr14:74479637-74480383	chr14	74479637	74480383	8	10	MIR4709	746	CKD3
chr14:74480100-74480450	chr14	74480100	74480450	2	13	MIR4709	350	CKD16
chr1:59732732-59733455	chr1	59732732	59733455	2	4	MIR4711	723	CKD12
chr1:59732992-59733362	chr1	59732992	59733362	4	10	MIR4711	370	CKD16
chr15:50360031-50360587	chr15	50360031	50360587	2	10	MIR4712	556	CKD6
chr15:50360309-50360644	chr15	50360309	50360644	0	8	MIR4712	335	CKD12
chr15:50360321-50360834	chr15	50360321	50360834	16	24	MIR4712	513	CKD13
chr15:50360219-50360793	chr15	50360219	50360793	0	6	MIR4712	574	CKD21
chr15:51241936-51242320	chr15	51241936	51242320	10	6	MIR4713	384	CKD20
chr15:51241898-51243480	chr15	51241898	51243480	0	8	MIR4713	1582	CKD25
chr15:98784221-98784571	chr15	98784221	98784571	0	6	MIR4714	350	CKD4
chr15:98784379-98785003	chr15	98784379	98785003	18	9	MIR4714	624	CKD9
chr15:98784222-98784560	chr15	98784222	98784560	6	14	MIR4714	338	HF5
chr15:25848465-25849258	chr15	25848465	25849258	6	8	MIR4715	793	CKD13

chr16:2274508-2275047	chr16	2274508	2275047	30	75	MIR4717	539	CKD3
chr16:2274402-2274766	chr16	2274402	2274766	8	30	MIR4717	364	CKD6
chr16:12719534-12720390	chr16	12719534	12720390	15	44	MIR4718	856	CKD8
chr16:12720100-12721176	chr16	12720100	12721176	40	82	MIR4718	1076	HM6
chr16:76868312-76869355	chr16	76868312	76869355	0	4	MIR4719	1043	CKD13
chr16:81384727-81385110	chr16	81384727	81385110	24	36	MIR4720	383	CKD16
chr16:81384932-81385300	chr16	81384932	81385300	6	15	MIR4720	368	CKD16
chr16:81384781-81385136	chr16	81384781	81385136	177	429	MIR4720	355	CKD22
chr16:28843426-28844434	chr16	28843426	28844434	2	6	MIR4721	1008	CKD3
chr16:28843189-28844065	chr16	28843189	28844065	2	4	MIR4721	876	CKD11
chr16:88716131-88716652	chr16	88716131	88716652	10	19	MIR4722	521	CKD13
chr16:88715815-88716407	chr16	88715815	88716407	12	10	MIR4722	592	CKD25
chr17:28360490-28360826	chr17	28360490	28360826	0	8	MIR4723	336	CKD12
chr17:31574900-31575926	chr17	31574900	31575926	9	17	MIR4725	1026	CKD16
chr17:31575229-31575571	chr17	31575229	31575571	1	19	MIR4725	342	CKD25
chr17:39725976-39726827	chr17	39725976	39726827	6	11	MIR4728	851	CKD12
chr17:39726438-39727112	chr17	39726438	39727112	370	548	MIR4728	674	CKD18
chr17:59365608-59367141	chr17	59365608	59367141	12	26	MIR4729	1533	CKD3
chr17:59365943-59366270	chr17	59365943	59366270	1	7	MIR4729	327	CKD9
chr17:59365373-59366353	chr17	59365373	59366353	18	39	MIR4729	980	CKD13
chr17:59365933-59366158	chr17	59365933	59366158	4	8	MIR4729	225	CKD18
chr17:59365879-59366207	chr17	59365879	59366207	6	25	MIR4729	328	CKD25
chr17:59365879-59366466	chr17	59365879	59366466	32	24	MIR4729	587	HM6
chr17:80419104-80419661	chr17	80419104	80419661	114	47	MIR4730	557	CKD22
chr17:15251373-15251701	chr17	15251373	15251701	0	11	MIR4731	328	CKD14
chr17:31094262-31094643	chr17	31094262	31094643	0	8	MIR4733	381	CKD14
chr17:31094285-31094588	chr17	31094285	31094588	17	51	MIR4733	303	CKD24
chr1:196582401-196582756	chr1	196582401	196582756	2	6	MIR4735	355	CKD22
chr1:196581946-196582545	chr1	196581946	196582545	12	20	MIR4735	599	HM11
chr17:58335916-58336268	chr17	58335916	58336268	6	33	MIR4736	352	CKD20
chr17:58335932-58336660	chr17	58335932	58336660	12	76	MIR4736	728	HM1
chr17:58335712-58336478	chr17	58335712	58336478	169	137	MIR4736	766	HM9
chr17:58335723-58336059	chr17	58335723	58336059	0	7	MIR4736	336	HM19
chr17:79707122-79707489	chr17	79707122	79707489	4	5	MIR4739	367	CKD8
chr17:79707090-79707494	chr17	79707090	79707494	4	24	MIR4739	404	CKD14
chr17:79706989-79707365	chr17	79706989	79707365	1668	1708	MIR4739	376	CKD17
chr17:79706989-79707365	chr17	79706989	79707365	3	6	MIR4739	376	CKD18
chr17:79706988-79707365	chr17	79706988	79707365	0	6	MIR4739	377	CKD25
chr17:81400675-81401003	chr17	81400675	81401003	13	42	MIR4740	328	CKD10
chr1:224398078-224398432	chr1	224398078	224398432	20	53	MIR4742	354	CKD24
chr18:49049591-49049785	chr18	49049591	49049785	2	18	MIR4744	194	HM13
chr19:4445816-4446195	chr19	4445816	4446195	14	31	MIR4746	379	CKD9
chr19:4445830-4446166	chr19	4445830	4446166	3	16	MIR4746	336	CKD13
chr19:4445821-4446176	chr19	4445821	4446176	189	333	MIR4746	355	CKD20
chr19:4445817-4446155	chr19	4445817	4446155	5	6	MIR4746	338	CKD25
chr19:4932555-4933232	chr19	4932555	4933232	34	89	MIR4747	677	CKD8
chr19:4932541-4932878	chr19	4932541	4932878	12	88	MIR4747	337	CKD18
chr19:4932361-4932746	chr19	4932361	4932746	31	47	MIR4747	385	CKD20
chr19:4932198-4933521	chr19	4932198	4933521	92	80	MIR4747	1323	HM1
chr19:10780012-10780360	chr19	10780012	10780360	19	53	MIR4748	348	CKD4
chr19:10780057-10780389	chr19	10780057	10780389	10	27	MIR4748	332	CKD7
chr19:10779129-10780520	chr19	10779129	10780520	2	6	MIR4748	1391	CKD16
chr19:49854301-49855314	chr19	49854301	49855314	12	57	MIR4749	1013	CKD16
chr19:49854559-49854749	chr19	49854559	49854749	6	189	MIR4749	190	HM13
chr19:49887558-49888292	chr19	49887558	49888292	7	11	MIR4750	734	CKD13
chr19:49887969-49888285	chr19	49887969	49888285	6	18	MIR4750	316	CKD18
chr19:49932868-49933203	chr19	49932868	49933203	0	10	MIR4751	335	CKD14
chr1:235189962-235190329	chr1	235189962	235190329	229	663	MIR4753	367	CKD4
chr1:235189655-235190384	chr1	235189655	235190384	15	18	MIR4753	729	CKD13
chr1:235189588-235191074	chr1	235189588	235191074	8	7	MIR4753	1486	CKD21
chr1:235189864-235190223	chr1	235189864	235190223	8	6	MIR4753	359	CKD25
chr19:58386756-58387126	chr19	58386756	58387126	360	1032	MIR4754	370	HM13
chr20:34049086-34049268	chr20	34049086	34049268	0	6	MIR4755	182	HM14
chr20:54067712-54068783	chr20	54067712	54068783	35	30	MIR4756	1071	CKD3
chr20:54068364-54068709	chr20	54068364	54068709	6	5	MIR4756	345	CKD16
chr20:54068105-54069092	chr20	54068105	54069092	4	8	MIR4756	987	CKD25
chr20:54068131-54069261	chr20	54068131	54069261	8	14	MIR4756	1130	CKD25
chr21:26953204-26954761	chr21	26953204	26954761	8	27	MIR4759	1557	CKD13
chr21:40212162-40212531	chr21	40212162	40212531	0	15	MIR4760	369	CKD3
chr21:40212264-40212599	chr21	40212264	40212599	0	6	MIR4760	335	CKD25
chr22:19963680-19964049	chr22	19963680	19964049	3	6	MIR4761	369	CKD8
chr22:19963506-19963861	chr22	19963506	19963861	4	20	MIR4761	355	HF6
chr22:45760230-45761179	chr22	45760230	45761179	0	26	MIR4762	949	CKD13
chr22:45760387-45760774	chr22	45760387	45760774	6	8	MIR4762	387	CKD18
chr22:45759930-45760985	chr22	45759930	45760985	90	106	MIR4762	1055	HF3
chr22:46113471-46113854	chr22	46113471	46113854	2	12	MIR4763	383	CKD13
chr22:46113465-46113793	chr22	46113465	46113793	5	23	MIR4763	328	CKD20
chr22:33436325-33437631	chr22	33436325	33437631	4	5	MIR4764	1306	CKD13

chr2:32635020-32635377	chr2	32635020	32635377	12	45	MIR4765	357	CKD8
chr2:32635209-32635373	chr2	32635209	32635373	0	27	MIR4765	164	CKD14
chr2:32635165-32635549	chr2	32635165	32635549	51	69	MIR4765	384	CKD24
chrX:6383784-6384163	chrX	6383784	6384163	4	8	MIR4770	379	HF5
chr2:102432215-102432961	chr2	102432215	102432961	10	23	MIR4772	746	HF5
chr2:168582326-168583415	chr2	168582326	168583415	2	8	MIR4774	1089	CKD3
chr2:168582938-168583285	chr2	168582938	168583285	0	8	MIR4774	347	CKD15
chr2:207754521-207754913	chr2	207754521	207754913	0	6	MIR4775	392	CKD9
chr2:207754740-207755109	chr2	207754740	207755109	1	10	MIR4775	369	CKD16
chr2:212926023-212926370	chr2	212926023	212926370	0	6	MIR4776-1	347	CKD8
chr2:212925761-212926478	chr2	212925761	212926478	2	10	MIR4776-1	717	CKD9
chr2:212926076-212926408	chr2	212926076	212926408	4	33	MIR4776-1	332	CKD16
chr2:212926023-212926370	chr2	212926023	212926370	0	6	MIR4776-2	347	CKD8
chr2:212925761-212926478	chr2	212925761	212926478	2	10	MIR4776-2	717	CKD9
chr2:212926076-212926408	chr2	212926076	212926408	4	33	MIR4776-2	332	CKD16
chr2:231362484-231362856	chr2	231362484	231362856	12	25	MIR4777	372	CKD8
chr2:66358000-66360978	chr2	66358000	66360978	0	15	MIR4778	2978	CKD25
chr2:88082392-88083343	chr2	88082392	88083343	6	15	MIR4780	951	CKD25
chr2:88082500-88082736	chr2	88082500	88082736	2	18	MIR4780	236	HM19
chr1:54053904-54054446	chr1	54053904	54054446	16	15	MIR4781	542	CKD4
chr1:54053949-54054199	chr1	54053949	54054199	0	14	MIR4781	250	CKD4
chr1:54054044-54054415	chr1	54054044	54054415	2	7	MIR4781	371	CKD12
chr2:127423501-127423818	chr2	127423501	127423818	1	7	MIR4783	317	CKD20
chr2:131491160-131491495	chr2	131491160	131491495	17	33	MIR4784	335	HF5
chr2:239942125-239943111	chr2	239942125	239943111	3	12	MIR4786	986	CKD3
chr2:239942736-239943359	chr2	239942736	239943359	6	13	MIR4786	623	CKD22
chr2:239942727-239943101	chr2	239942727	239943101	4	10	MIR4786	374	CKD25
chr3:19314535-19315487	chr3	19314535	19315487	0	4	MIR4791	952	CKD3
chr1:64579733-64580116	chr1	64579733	64580116	6	12	MIR4794	383	CKD9
chr3:87225353-87226353	chr3	87225353	87226353	4	11	MIR4795	1000	CKD3
chr3:87226118-87226455	chr3	87226118	87226455	0	10	MIR4795	337	CKD14
chr4:147782326-147782765	chr4	147782326	147782765	2	3	MIR4799	439	CKD4
chr4:147782489-147782710	chr4	147782489	147782710	0	18	MIR4799	221	CKD9
chr4:147782569-147782747	chr4	147782569	147782747	1	19	MIR4799	178	CKD22
chr4:2249684-2250687	chr4	2249684	2250687	8	24	MIR4800	1003	CKD13
chr4:37241168-37242916	chr4	37241168	37242916	9	16	MIR4801	1748	CKD3
chr4:40501665-40502198	chr4	40501665	40502198	7	7	MIR4802	533	CKD3
chr4:40501975-40502531	chr4	40501975	40502531	48	40	MIR4802	556	CKD3
chr4:40501917-40502253	chr4	40501917	40502253	6	4	MIR4802	336	CKD12
chr4:40502007-40502198	chr4	40502007	40502198	2	14	MIR4802	191	CKD12
chr4:40502006-40502398	chr4	40502006	40502398	30	34	MIR4802	392	CKD13
chr4:40500382-40502152	chr4	40500382	40502152	4	37	MIR4802	1770	CKD16
chr4:40502008-40502198	chr4	40502008	40502198	0	28	MIR4802	190	CKD18
chr5:72168999-72169875	chr5	72168999	72169875	48	29	MIR4803	876	CKD13
chr5:72169198-72169547	chr5	72169198	72169547	0	8	MIR4803	349	CKD13
chr11:2133968-2134324	chr11	2133968	2134324	2	9	MIR483	356	CKD12
chr8:41659453-41660544	chr8	41659453	41660544	15	12	MIR486-1	1091	CKD3
chr8:41659453-41660544	chr8	41659453	41660544	15	12	MIR486-2	1091	CKD3
chr14:101052281-101053050	chr14	101052281	101053050	3	7	MIR487A	769	CKD13
chr14:101046086-101046632	chr14	101046086	101046632	108	38	MIR487B	546	CKD10
chr14:101046316-101046701	chr14	101046316	101046701	0	6	MIR487B	385	CKD12
chr14:101046324-101046704	chr14	101046324	101046704	16	27	MIR487B	380	CKD20
chr14:101046350-101046862	chr14	101046350	101046862	0	21	MIR487B	512	CKD25
chr1:177029018-177030007	chr1	177029018	177030007	12	11	MIR488	989	CKD16
chr7:93483361-93484082	chr7	93483361	93484082	3	8	MIR489	721	CKD13
chr7:93483836-93484229	chr7	93483836	93484229	8	8	MIR489	393	HM3
chr7:136902664-136903868	chr7	136902664	136903868	6	10	MIR490	1204	CKD13
chr9:20715864-20716238	chr9	20715864	20716238	11	12	MIR491	374	HM3
chr12:94834331-94834660	chr12	94834331	94834660	4	5	MIR492	329	CKD12
chr12:94834316-94834710	chr12	94834316	94834710	30	50	MIR492	394	HM6
chr14:101033755-101034460	chr14	101033755	101034460	12	23	MIR495	705	CKD25
chr14:101033744-101033946	chr14	101033744	101033946	1	12	MIR495	202	HM1
chr14:101032424-101033998	chr14	101032424	101033998	14	10	MIR495	1574	HM9
chr14:101060395-101060746	chr14	101060395	101060746	6	9	MIR496	351	CKD21
chr17:7017723-7018096	chr17	7017723	7018096	10	12	MIR497	373	HM5
chr19:53673988-53674415	chr19	53673988	53674415	4	33	MIR498	427	HM6
chr20:34990210-34990538	chr20	34990210	34990538	5	12	MIR499A	328	HM9
chr20:34990210-34990538	chr20	34990210	34990538	5	12	MIR499B	328	HM9
chr2:75090422-75090941	chr2	75090422	75090941	12	20	MIR5000	519	CKD9
chr2:75090748-75091121	chr2	75090748	75091121	8	23	MIR5000	373	CKD22
chr3:124132907-124133261	chr3	124132907	124133261	0	6	MIR5002	354	CKD3
chr3:124132753-124133111	chr3	124132753	124133111	18	26	MIR5002	358	CKD20
chr3:124132646-124133269	chr3	124132646	124133269	108	130	MIR5002	623	CKD22
chr5:172662016-172662375	chr5	172662016	172662375	22	32	MIR5003	359	CKD10
chr13:41568103-41569204	chr13	41568103	41569204	8	22	MIR5006	1101	CKD3
chr13:41568248-41568630	chr13	41568248	41568630	16	14	MIR5006	382	CKD8
chr13:41567817-41569369	chr13	41567817	41569369	9	14	MIR5006	1552	CKD21
chr15:89883717-89884085	chr15	89883717	89884085	5	8	MIR5009	368	CKD10

chrX:50008428-50008965	chrX	50008428	50008965	28	13	MIR500A	537	CKD4
chrX:50008388-50009177	chrX	50008388	50009177	16	35	MIR500A	789	CKD16
chrX:50010563-50010926	chrX	50010563	50010926	4	14	MIR500B	363	CKD3
chrX:50009278-50011419	chrX	50009278	50011419	6	4	MIR500B	2141	CKD25
chrX:50009273-50009826	chrX	50009273	50009826	15	27	MIR501	553	CKD9
chrX:50009427-50009827	chrX	50009427	50009827	3	6	MIR501	400	CKD16
chrX:50009278-50011419	chrX	50009278	50011419	6	4	MIR501	2141	CKD25
chr17:42513816-42515098	chr17	42513816	42515098	2	18	MIR5010	1282	CKD16
chr17:42513880-42514692	chr17	42513880	42514692	4	10	MIR5010	812	CKD25
chrX:50014375-50014721	chrX	50014375	50014721	14	8	MIR502	346	CKD12
chrX:134546045-134546413	chrX	134546045	134546413	1	7	MIR503	368	CKD15
chrX:134546213-134546532	chrX	134546213	134546532	26	103	MIR503	319	CKD20
chrX:138667628-138667868	chrX	138667628	138667868	0	4	MIR504	240	CKD4
chrX:138667591-138667941	chrX	138667591	138667941	9	44	MIR504	350	CKD20
chr17:64500695-64501391	chr17	64500695	64501391	12	4	MIR5047	696	CKD13
chr17:64501123-64501462	chr17	64501123	64501462	12	38	MIR5047	339	CKD14
chrX:139924035-139925582	chrX	139924035	139925582	0	7	MIR505	1547	CKD25
chrX:147230619-147231000	chrX	147230619	147231000	1	6	MIR506	381	CKD13
chrX:147230641-147230999	chrX	147230641	147230999	33	73	MIR506	358	CKD22
chrX:147230954-147231155	chrX	147230954	147231155	0	6	MIR507	201	CKD6
chrX:147236860-147237164	chrX	147236860	147237164	2	11	MIR508	304	CKD21
chr1:148334430-148334797	chr1	148334430	148334797	2	19	MIR5087	367	CKD3
chr1:148334225-148334573	chr1	148334225	148334573	7	18	MIR5087	348	CKD4
chr1:148334445-148334874	chr1	148334445	148334874	2	7	MIR5087	429	CKD21
chr17:46972625-46974557	chr17	46972625	46974557	4	18	MIR5089	1932	HF5
chr7:102465542-102466258	chr7	102465542	102466258	2	13	MIR5090	716	CKD3
chr7:102465599-102466245	chr7	102465599	102466245	0	4	MIR5090	646	CKD25
chr7:102465412-102466362	chr7	102465412	102466362	9	16	MIR5090	950	CKD25
chr4:13627802-13628074	chr4	13627802	13628074	6	23	MIR5091	272	CKD8
chr3:125151273-125152075	chr3	125151273	125152075	4	12	MIR5092	802	CKD9
chr3:125151093-125151804	chr3	125151093	125151804	16	28	MIR5092	711	HM6
chrX:147257774-147258930	chrX	147257774	147258930	2	7	MIR509-2	1156	CKD25
chr16:85306120-85306335	chr16	85306120	85306335	0	9	MIR5093	215	CKD13
chr16:85306152-85306559	chr16	85306152	85306559	17	23	MIR5093	407	CKD16
chr16:85306154-85307550	chr16	85306154	85307550	88	156	MIR5093	1396	HM1
chr15:89850473-89850995	chr15	89850473	89850995	8	10	MIR5094	522	CKD8
chrX:147272282-147272641	chrX	147272282	147272641	5	11	MIR510	359	HF4
chr10:42997352-42997859	chr10	42997352	42997859	4	8	MIR5100	507	CKD3
chr10:42997540-42997900	chr10	42997540	42997900	8	55	MIR5100	360	HM5
chr10:17845037-17845226	chr10	17845037	17845226	1	20	MIR511	189	CKD7
chr10:17845041-17845407	chr10	17845041	17845407	10	13	MIR511	366	CKD10
chr10:17845061-17845413	chr10	17845061	17845413	2	9	MIR511	352	HM1
chrX:147212915-147213968	chrX	147212915	147213968	22	32	MIR513A1	1053	CKD13
chrX:147213217-147213910	chrX	147213217	147213910	9	55	MIR513A1	693	CKD25
chrX:147198598-147199268	chrX	147198598	147199268	9	9	MIR513B	670	CKD21
chrX:147189433-147189789	chrX	147189433	147189789	0	5	MIR513C	356	CKD13
chr19:53678808-53679333	chr19	53678808	53679333	45	14	MIR515-1	525	CKD12
chr19:53678905-53679333	chr19	53678905	53679333	14	11	MIR515-1	428	HM19
chr19:53756224-53757173	chr19	53756224	53757173	21	29	MIR516A1	949	CKD3
chr19:53756721-53757307	chr19	53756721	53757307	9	13	MIR516A1	586	CKD12
chr19:53756477-53757651	chr19	53756477	53757651	2	13	MIR516A1	1174	CKD21
chr19:53760806-53761415	chr19	53760806	53761415	3	8	MIR516A2	609	CKD8
chr19:53736718-53737078	chr19	53736718	53737078	0	5	MIR516B1	360	CKD8
chr19:53736719-53737079	chr19	53736719	53737079	12	16	MIR516B1	360	CKD10
chr19:53725088-53726184	chr19	53725088	53726184	33	65	MIR516B2	1096	CKD13
chr19:53710942-53712653	chr19	53710942	53712653	14	40	MIR517A	1711	CKD16
chr19:53720974-53721355	chr19	53720974	53721355	1	4	MIR517B	381	CKD25
chr1:161227126-161227497	chr1	161227126	161227497	3	7	MIR5187	371	CKD13
chr1:161227125-161227482	chr1	161227125	161227482	5	10	MIR5187	357	HF5
chr12:124915527-124915939	chr12	124915527	124915939	8	10	MIR5188	412	CKD18
chr16:88468363-88469256	chr16	88468363	88469256	27	31	MIR5189	893	HM11
chr19:53730661-53731283	chr19	53730661	53731283	22	50	MIR518A1	622	CKD3
chr19:53739285-53739684	chr19	53739285	53739684	60	51	MIR518A2	399	HM19
chr19:53701736-53703283	chr19	53701736	53703283	4	6	MIR518B	1547	CKD13
chr19:53708668-53709246	chr19	53708668	53709246	2	4	MIR518C	578	CKD11
chr19:53734752-53735196	chr19	53734752	53735196	2	5	MIR518D	444	CKD3
chr19:53729743-53729943	chr19	53729743	53729943	0	28	MIR518E	200	CKD22
chr18:13459670-13460115	chr18	13459670	13460115	8	14	MIR5190	445	CKD12
chr18:13459751-13460089	chr18	13459751	13460089	8	8	MIR5190	338	CKD20
chr18:13459941-13460296	chr18	13459941	13460296	17	39	MIR5190	355	HM7
chr1:201719130-201719912	chr1	201719130	201719912	3	4	MIR5191	782	CKD25
chr1:201719380-201719747	chr1	201719380	201719747	0	8	MIR5191	367	HM19
chr2:62205428-62205959	chr2	62205428	62205959	2	8	MIR5192	531	CKD14
chr2:62205477-62206889	chr2	62205477	62206889	5	20	MIR5192	1412	HF5
chr8:130008168-130008509	chr8	130008168	130008509	6	19	MIR5194	341	CKD8
chr8:130008245-130008603	chr8	130008245	130008603	9	9	MIR5194	358	CKD25
chr5:143679540-143679989	chr5	143679540	143679989	15	13	MIR5197	449	CKD3
chr19:53751862-53753152	chr19	53751862	53753152	89	24	MIR519A1	1290	CKD10

chr19:53751758-53752499	chr19	53751758	53752499	24	56	MIR519A1	741	CKD16
chr19:53761471-53762716	chr19	53761471	53762716	20	52	MIR519A2	1245	CKD16
chr19:53762264-53762501	chr19	53762264	53762501	0	6	MIR519A2	237	CKD18
chr19:53762021-53762786	chr19	53762021	53762786	6	10	MIR519A2	765	CKD21
chr19:53679883-53680270	chr19	53679883	53680270	3	14	MIR519E	387	CKD12
chr19:53679281-53680325	chr19	53679281	53680325	16	60	MIR519E	1044	CKD16
chr19:53690862-53691450	chr19	53690862	53691450	14	16	MIR520A	588	CKD8
chr19:53690820-53691235	chr19	53690820	53691235	10	6	MIR520A	415	CKD16
chr19:53700841-53702360	chr19	53700841	53702360	7	12	MIR520B	1519	CKD3
chr19:53706928-53708140	chr19	53706928	53708140	12	5	MIR520C	1212	CKD16
chr19:53675567-53675959	chr19	53675567	53675959	6	13	MIR520E	392	CKD8
chr19:53681886-53682458	chr19	53681886	53682458	22	16	MIR520F	572	CKD9
chr19:53682100-53683553	chr19	53682100	53683553	12	14	MIR520F	1453	CKD9
chr19:53682100-53682496	chr19	53682100	53682496	10	12	MIR520F	396	HM6
chr19:53748081-53749454	chr19	53748081	53749454	45	107	MIR521-1	1373	HM1
chr19:53716469-53716849	chr19	53716469	53716849	4	6	MIR521-2	380	HM1
chr19:53716104-53716896	chr19	53716104	53716896	82	119	MIR521-2	792	HM9
chr19:53750897-53751814	chr19	53750897	53751814	8	15	MIR522	917	CKD3
chr19:53698251-53698611	chr19	53698251	53698611	0	4	MIR523	360	CKD16
chr19:53710783-53711121	chr19	53710783	53711121	0	4	MIR524	338	CKD4
chr19:53710744-53711329	chr19	53710744	53711329	1	4	MIR524	585	CKD12
chr19:53710942-53712653	chr19	53710942	53712653	14	40	MIR524	1711	CKD16
chr19:53696673-53697854	chr19	53696673	53697854	54	14	MIR525	1181	CKD3
chr19:53697472-53697854	chr19	53697472	53697854	12	21	MIR525	382	CKD14
chr19:53705961-53706377	chr19	53705961	53706377	24	5	MIR526A1	416	CKD21
chr19:53693696-53694513	chr19	53693696	53694513	5	16	MIR526B	817	CKD22
chr19:53753540-53754719	chr19	53753540	53754719	24	42	MIR527	1179	CKD16
chr19:53753876-53754240	chr19	53753876	53754240	86	9	MIR527	364	HF3
chr19:53753930-53754110	chr19	53753930	53754110	4	10	MIR527	180	HF3
chr14:101064484-101064800	chr14	101064484	101064800	0	28	MIR541	316	CKD8
chr14:101064476-101064812	chr14	101064476	101064812	17	69	MIR541	336	CKD10
chr14:101063579-101064706	chr14	101063579	101064706	1	9	MIR541	1127	HF5
chr14:101031755-101032120	chr14	101031755	101032120	51	130	MIR543	365	CKD10
chr14:101031687-101032067	chr14	101031687	101032067	29	89	MIR543	380	CKD20
chr14:101031772-101032122	chr14	101031772	101032122	0	8	MIR543	350	CKD21
chr14:101031896-101032249	chr14	101031896	101032249	0	6	MIR543	353	CKD21
chr14:101031872-101032082	chr14	101031872	101032082	1	18	MIR543	210	HM11
chr14:101048093-101049658	chr14	101048093	101049658	10	20	MIR544A	1565	CKD21
chr3:124732038-124732547	chr3	124732038	124732547	12	8	MIR544B	509	CKD13
chr6:18569284-18573723	chr6	18569284	18573723	7	32	MIR548A1	4439	CKD3
chr6:18571497-18572246	chr6	18571497	18572246	15	12	MIR548A1	749	CKD3
chr6:135238914-135239294	chr6	135238914	135239294	0	9	MIR548A2	380	CKD4
chr6:135238899-135239263	chr6	135238899	135239263	14	282	MIR548A2	364	CKD8
chr8:104484307-104484507	chr8	104484307	104484507	0	5	MIR548A3	200	CKD6
chr8:104484308-104484668	chr8	104484308	104484668	7	10	MIR548A3	360	CKD6
chr8:104484291-104484670	chr8	104484291	104484670	2	4	MIR548A3	379	CKD22
chr8:123345942-123348424	chr8	123345942	123348424	3	20	MIR548AA1	2482	CKD16
chr8:123347838-123348680	chr8	123347838	123348680	4	4	MIR548AA1	842	CKD21
chr8:123347927-123348265	chr8	123347927	123348265	0	8	MIR548AA1	338	CKD21
chr17:67471473-67474050	chr17	67471473	67474050	0	7	MIR548AA2	2577	CKD3
chr3:103523830-103525214	chr3	103523830	103525214	6	18	MIR548AB	1384	CKD21
chr1:116559493-116560694	chr1	116559493	116560694	6	6	MIR548AC	1201	HM1
chr2:35471364-35471751	chr2	35471364	35471751	8	17	MIR548AD	387	CKD20
chr5:58528990-58530667	chr5	58528990	58530667	8	16	MIR548AE2	1677	CKD3
chr5:58529857-58530427	chr5	58529857	58530427	0	4	MIR548AE2	570	CKD16
chr20:60564496-60565049	chr20	60564496	60565049	3	15	MIR548AG2	553	CKD21
chr6:99124552-99125092	chr6	99124552	99125092	0	9	MIR548AI	540	CKD25
chr6:132114476-132115899	chr6	132114476	132115899	1	8	MIR548AJ1	1423	CKD13
chr6:132114963-132115824	chr6	132114963	132115824	9	8	MIR548AJ1	861	CKD25
chr10:12130580-12130925	chr10	12130580	12130925	16	46	MIR548AK	345	CKD4
chr10:12130670-12130868	chr10	12130670	12130868	0	9	MIR548AK	198	CKD10
chr11:74399114-74399740	chr11	74399114	74399740	6	8	MIR548AL	626	CKD3
chr15:85825413-85826256	chr15	85825413	85826256	18	19	MIR548AP	843	CKD12
chr15:85825423-85825798	chr15	85825423	85825798	0	6	MIR548AP	375	CKD12
chr15:85825612-85825949	chr15	85825612	85825949	0	42	MIR548AP	337	CKD12
chr15:85825451-85825779	chr15	85825451	85825779	3	18	MIR548AP	328	CKD20
chr15:85825200-85825739	chr15	85825200	85825739	6	9	MIR548AP	539	CKD25
chr13:114244330-114244974	chr13	114244330	114244974	8	33	MIR548AR	644	CKD12
chr17:42494688-42494952	chr17	42494688	42494952	1	10	MIR548AT	264	CKD3
chr17:42494687-42495042	chr17	42494687	42495042	0	13	MIR548AT	355	CKD8
chr9:132945408-132945788	chr9	132945408	132945788	6	6	MIR548AW	380	HM3
chr8:119325156-119325480	chr8	119325156	119325480	2	14	MIR548AZ	324	CKD22
chr3:60617545-60618127	chr3	60617545	60618127	6	9	MIR548BB	582	CKD13
chr3:60617370-60617906	chr3	60617370	60617906	6	4	MIR548BB	536	CKD21
chr12:64621685-64622739	chr12	64621685	64622739	15	6	MIR548C	1054	CKD12
chr8:123345942-123348424	chr8	123345942	123348424	3	20	MIR548D1	2482	CKD16
chr8:123347838-123348680	chr8	123347838	123348680	4	4	MIR548D1	842	CKD21
chr8:123347927-123348265	chr8	123347927	123348265	0	8	MIR548D1	338	CKD21

chr17:67471473-67474050	chr17	67471473	67474050	0	7	MIR548D2	2577	CKD3
chr10:54607479-54608165	chr10	54607479	54608165	16	12	MIR548F1	686	CKD21
chr10:54607368-54608124	chr10	54607368	54608124	12	29	MIR548F1	756	CKD22
chr7:147377977-147378730	chr7	147377977	147378730	12	20	MIR548F4	753	CKD8
chrX:32641124-32641740	chrX	32641124	32641740	0	9	MIR548F5	616	CKD3
chr14:64094204-64095141	chr14	64094204	64095141	8	8	MIR548H1	937	CKD3
chr16:11306043-11306970	chr16	11306043	11306970	10	25	MIR548H2	927	CKD21
chr17:13543416-13544436	chr17	13543416	13544436	6	6	MIR548H3	1020	CKD22
chr6:131791820-131792362	chr6	131791820	131792362	11	9	MIR548H5	542	CKD13
chr6:131791910-131792453	chr6	131791910	131792453	8	6	MIR548H5	543	CKD16
chrX:84225431-84225972	chrX	84225431	84225972	12	10	MIR548I4	541	CKD3
chrX:84225658-84225858	chrX	84225658	84225858	65	369	MIR548I4	200	HM6
chr22:26555211-26555548	chr22	26555211	26555548	0	15	MIR548J	337	CKD20
chr11:94466471-94467062	chr11	94466471	94467062	12	41	MIR548L	591	CKD20
chr11:94465215-94466605	chr11	94465215	94466605	12	30	MIR548L	1390	HM1
chr11:94466326-94466669	chr11	94466326	94466669	2	8	MIR548L	343	HM19
chr7:34940702-34940903	chr7	34940702	34940903	0	10	MIR548N	201	CKD18
chr7:102405555-102405919	chr7	102405555	102405919	30	65	MIR548O	364	CKD18
chr7:102405727-102406049	chr7	102405727	102406049	0	4	MIR548O	322	HM3
chr10:12725243-12725431	chr10	12725243	12725431	0	37	MIR548Q	188	CKD17
chr10:12725209-12726111	chr10	12725209	12726111	0	4	MIR548Q	902	CKD21
chr10:12724948-12725635	chr10	12724948	12725635	6	10	MIR548Q	687	CKD22
chr2:11767333-11767687	chr2	11767333	11767687	28	33	MIR548S	354	CKD3
chr2:11767422-11767791	chr2	11767422	11767791	27	52	MIR548S	369	CKD9
chr2:11767428-11767622	chr2	11767428	11767622	0	9	MIR548S	194	CKD18
chr2:11767138-11767531	chr2	11767138	11767531	15	15	MIR548S	393	CKD25
chr2:11767252-11767651	chr2	11767252	11767651	12	12	MIR548S	399	CKD25
chr2:11767303-11768070	chr2	11767303	11768070	12	11	MIR548S	767	CKD25
chr4:173268067-173268256	chr4	173268067	173268256	0	10	MIR548T	189	CKD8
chr4:173268124-173268490	chr4	173268124	173268490	4	27	MIR548T	366	CKD13
chr4:173267934-173268292	chr4	173267934	173268292	4	4	MIR548T	358	CKD16
chr16:26025151-26025498	chr16	26025151	26025498	6	16	MIR548W	347	CKD9
chr16:26025182-26025537	chr16	26025182	26025537	0	3	MIR548W	355	CKD21
chr13:65966198-65966537	chr13	65966198	65966537	4	32	MIR548X2	339	CKD3
chr13:65966170-65966837	chr13	65966170	65966837	9	21	MIR548X2	667	CKD12
chr13:65966141-65966766	chr13	65966141	65966766	0	3	MIR548X2	625	CKD21
chr13:65966210-65967155	chr13	65966210	65967155	3	10	MIR548X2	945	CKD21
chr14:47760972-47761716	chr14	47760972	47761716	1	8	MIR548Y	744	CKD4
chr14:47760873-47761396	chr14	47760873	47761396	24	30	MIR548Y	523	CKD6
chr14:47760827-47761532	chr14	47760827	47761532	3	7	MIR548Y	705	CKD8
chr14:47760947-47761306	chr14	47760947	47761306	6	10	MIR548Y	359	HM5
chr12:64621685-64622739	chr12	64621685	64622739	15	6	MIR548Z	1054	CKD12
chr7:30289361-30290267	chr7	30289361	30290267	12	11	MIR550A1	906	CKD3
chr7:30289361-30290267	chr7	30289361	30290267	12	11	MIR550B1	906	CKD3
chr3:168551739-168552522	chr3	168551739	168552522	15	24	MIR551B	783	CKD13
chr1:155345369-155347659	chr1	155345369	155347659	140	49	MIR555	2290	CKD15
chr1:168375300-168377023	chr1	168375300	168377023	2	10	MIR557	1723	CKD3
chr22:22886262-22886624	chr22	22886262	22886624	10	26	MIR5571	362	CKD3
chr22:22886083-22886624	chr22	22886083	22886624	18	27	MIR5571	541	CKD4
chr22:22886069-22886678	chr22	22886069	22886678	21	37	MIR5571	609	CKD21
chr22:22886030-22886444	chr22	22886030	22886444	19	17	MIR5571	414	HM11
chr15:80580950-80581314	chr15	80580950	80581314	4	12	MIR5572	364	CKD9
chr15:80580612-80581313	chr15	80580612	80581313	4	8	MIR5572	701	CKD21
chr11:79422143-79422364	chr11	79422143	79422364	0	3	MIR5579	221	CKD3
chr11:79421863-79422234	chr11	79421863	79422234	2	7	MIR5579	371	CKD16
chr2:32531836-32532518	chr2	32531836	32532518	4	4	MIR558	682	CKD12
chr2:32531902-32532570	chr2	32531902	32532570	44	164	MIR558	668	CKD24
chr14:53948405-53948750	chr14	53948405	53948750	26	48	MIR5580	345	CKD8
chr14:53948405-53948774	chr14	53948405	53948774	4	14	MIR5580	369	CKD13
chr14:53948406-53948974	chr14	53948406	53948974	32	29	MIR5580	568	CKD16
chr14:53947555-53948550	chr14	53947555	53948550	2	22	MIR5580	995	CKD25
chr1:37500652-37501234	chr1	37500652	37501234	6	16	MIR5581	582	CKD4
chr1:37500780-37501171	chr1	37500780	37501171	25	7	MIR5581	391	CKD24
chr1:37500874-37501221	chr1	37500874	37501221	21	35	MIR5581	347	CKD24
chr1:37500564-37501112	chr1	37500564	37501112	8	15	MIR5581	548	HM1
chr1:44545377-44545910	chr1	44545377	44545910	12	12	MIR5584	533	HM1
chr1:32086806-32087153	chr1	32086806	32087153	30	6	MIR5585	347	CKD20
chr16:535231-535622	chr16	535231	535622	3	5	MIR5587	391	CKD16
chr16:535142-535466	chr16	535142	535466	4	22	MIR5587	324	CKD22
chr3:185253042-185253379	chr3	185253042	185253379	1	8	MIR5588	337	CKD20
chr2:134857335-134858171	chr2	134857335	134858171	8	4	MIR5590	836	CKD3
chr4:39411468-39412163	chr4	39411468	39412163	12	17	MIR5591	695	CKD16
chr4:39411444-39412109	chr4	39411444	39412109	9	27	MIR5591	665	HM1
chr4:39410607-39412506	chr4	39410607	39412506	5	10	MIR5591	1899	HM5
chr2:232172083-232172792	chr2	232172083	232172792	30	64	MIR562	709	CKD22
chr8:102125199-102125716	chr8	102125199	102125716	15	23	MIR5680	517	CKD8
chr8:102125237-102125601	chr8	102125237	102125601	3	12	MIR5680	364	CKD9
chr8:102125188-102125951	chr8	102125188	102125951	28	12	MIR5680	763	CKD12

chr8:102125399-102126089	chr8	102125399	102126089	32	58	MIR5680	690	CKD12
chr8:102125049-102125765	chr8	102125049	102125765	15	40	MIR5680	716	CKD13
chr8:102125199-102125557	chr8	102125199	102125557	2	16	MIR5680	358	CKD24
chr8:74548446-74548642	chr8	74548446	74548642	0	17	MIR5681A	196	CKD12
chr8:74548446-74548642	chr8	74548446	74548642	0	17	MIR5681B	196	CKD12
chr6:6168694-6169969	chr6	6168694	6169969	9	24	MIR5683	1275	CKD3
chr6:6169268-6169436	chr6	6169268	6169436	0	4	MIR5683	168	CKD6
chr6:6169195-6169432	chr6	6169195	6169432	4	132	MIR5683	237	HM3
chr6:6169080-6169438	chr6	6169080	6169438	0	7	MIR5683	358	HM6
chr19:12786531-12787330	chr19	12786531	12787330	4	6	MIR5684	799	CKD3
chr19:12787083-12788132	chr19	12787083	12788132	18	20	MIR5684	1049	CKD13
chr19:12786712-12787881	chr19	12786712	12787881	6	7	MIR5684	1169	CKD21
chr19:12786023-12787360	chr19	12786023	12787360	2	5	MIR5684	1337	CKD25
chr19:12786943-12787304	chr19	12786943	12787304	15	24	MIR5684	361	HM1
chr19:12787062-12787539	chr19	12787062	12787539	45	47	MIR5684	477	HM1
chr6:53276909-53277268	chr6	53276909	53277268	25	95	MIR5685	359	CKD8
chr6:53276771-53277533	chr6	53276771	53277533	5	42	MIR5685	762	CKD21
chr6:53276908-53277249	chr6	53276908	53277249	3	19	MIR5685	341	CKD25
chr5:55508224-55508980	chr5	55508224	55508980	14	29	MIR5687	756	CKD12
chr11:9090002-9090390	chr11	9090002	9090390	1	11	MIR5691	388	HM6
chr7:97963626-97963951	chr7	97963626	97963951	0	7	MIR5692A1	325	CKD3
chr7:97963493-97963852	chr7	97963493	97963852	34	36	MIR5692A1	359	CKD8
chr7:97963493-97963852	chr7	97963493	97963852	9	23	MIR5692A1	359	CKD13
chr7:97963611-97963811	chr7	97963611	97963811	0	24	MIR5692A1	200	HM15
chr21:42950873-42951243	chr21	42950873	42951243	21	24	MIR5692B	370	CKD4
chr21:42950847-42951773	chr21	42950847	42951773	4	4	MIR5692B	926	CKD13
chr5:135802743-135803810	chr5	135802743	135803810	1	4	MIR5692C1	1067	CKD16
chr7:97964290-97964659	chr7	97964290	97964659	0	7	MIR5692C2	369	CKD3
chr13:51348428-51348832	chr13	51348428	51348832	7	35	MIR5693	404	CKD16
chr13:51348428-51348832	chr13	51348428	51348832	11	9	MIR5693	404	HF4
chr13:51348299-51349002	chr13	51348299	51349002	8	18	MIR5693	703	HM1
chr14:67441681-67442008	chr14	67441681	67442008	8	15	MIR5694	327	CKD25
chr14:67441388-67442279	chr14	67441388	67442279	22	39	MIR5694	891	HM1
chr19:12920308-12920951	chr19	12920308	12920951	10	25	MIR5695	643	CKD21
chr1:154104227-154105195	chr1	154104227	154105195	15	8	MIR5698	968	CKD13
chr1:154104268-154105036	chr1	154104268	154105036	92	24	MIR5698	768	CKD13
chr1:154104453-154104639	chr1	154104453	154104639	5	16	MIR5698	186	CKD14
chr1:154104294-154105022	chr1	154104294	154105022	20	45	MIR5698	728	CKD16
chr1:154103924-154104880	chr1	154103924	154104880	12	4	MIR5698	956	CKD21
chr10:641186-641870	chr10	641186	641870	0	6	MIR5699	684	CKD3
chr10:641622-641979	chr10	641622	641979	12	20	MIR5699	357	CKD13
chr10:641185-641984	chr10	641185	641984	45	44	MIR5699	799	CKD18
chr10:641561-641904	chr10	641561	641904	0	10	MIR5699	343	CKD21
chr12:94561764-94562139	chr12	94561764	94562139	18	14	MIR5700	375	CKD8
chr12:94561524-94561861	chr12	94561524	94561861	3	12	MIR5700	337	CKD12
chr12:94561151-94562297	chr12	94561151	94562297	20	36	MIR5700	1146	CKD13
chr5:119154442-119154803	chr5	119154442	119154803	0	10	MIR5706	361	CKD4
chr5:119154622-119154946	chr5	119154622	119154946	0	8	MIR5706	324	CKD21
chr5:119154462-119154832	chr5	119154462	119154832	0	4	MIR5706	370	CKD25
chr5:119154461-119154804	chr5	119154461	119154804	4	17	MIR5706	343	HM19
chr7:158591047-158591807	chr7	158591047	158591807	24	33	MIR5707	760	CKD13
chr7:158590979-158591835	chr7	158590979	158591835	60	96	MIR5707	856	HM6
chr4:24519491-24520926	chr4	24519491	24520926	12	7	MIR573	1435	CKD15
chr4:82752731-82753487	chr4	82752731	82753487	2	8	MIR575	756	CKD3
chr4:82752527-82754076	chr4	82752527	82754076	48	153	MIR575	1549	CKD16
chr4:82752796-82753535	chr4	82752796	82753535	10	6	MIR575	739	CKD21
chr4:109487956-109489177	chr4	109487956	109489177	3	6	MIR576	1221	CKD13
chr4:114656667-114657191	chr4	114656667	114657191	10	10	MIR577	524	CKD4
chr5:36147854-36148204	chr5	36147854	36148204	0	5	MIR580	350	CKD18
chr5:53951343-53951690	chr5	53951343	53951690	0	9	MIR581	347	CKD9
chr6:126484481-126484818	chr6	126484481	126484818	2	6	MIR588	337	CKD9
chr7:5495444-5496087	chr7	5495444	5496087	6	22	MIR589	643	CKD4
chr7:5495650-5496013	chr7	5495650	5496013	25	67	MIR589	363	HM3
chr7:74191036-74191808	chr7	74191036	74191808	8	6	MIR590	772	CKD3
chr7:74191193-74192265	chr7	74191193	74192265	48	110	MIR590	1072	HM1
chr7:74190742-74191507	chr7	74190742	74191507	70	22	MIR590	765	HM5
chr7:96219577-96219965	chr7	96219577	96219965	37	133	MIR591	388	HM3
chr7:158531871-158532821	chr7	158531871	158532821	8	22	MIR595	950	CKD25
chr7:158532627-158533893	chr7	158532627	158533893	1	8	MIR595	1266	CKD25
chr8:11034658-11035321	chr8	11034658	11035321	6	6	MIR598	663	CKD13
chr8:11034975-11035751	chr8	11034975	11035751	1	5	MIR598	776	CKD13
chr8:11035193-11035595	chr8	11035193	11035595	617	949	MIR598	402	CKD17
chr8:99536236-99536856	chr8	99536236	99536856	4	21	MIR599	620	CKD3
chr9:123402303-123402671	chr9	123402303	123402671	7	15	MIR601	368	CKD3
chr9:137838285-137838623	chr9	137838285	137838623	4	23	MIR602	338	CKD7
chr9:137838314-137838679	chr9	137838314	137838679	37	58	MIR602	365	CKD14
chr9:137838256-137838651	chr9	137838256	137838651	37	91	MIR602	395	HM6
chr10:24275625-24275983	chr10	24275625	24275983	2	8	MIR603	358	CKD20

chr10:24275632-24275988	chr10	24275632	24275988	1	6	MIR603	356	HF6
chr10:29544908-29545330	chr10	29544908	29545330	8	8	MIR604	422	CKD18
chr10:51299263-51299762	chr10	51299263	51299762	2	6	MIR605	499	CKD16
chr1:63326801-63328280	chr1	63326801	63328280	2	22	MIR6068	1479	CKD3
chr1:63326851-63327605	chr1	63326851	63327605	18	22	MIR6068	754	CKD13
chr1:63326064-63326984	chr1	63326064	63326984	14	59	MIR6068	920	CKD16
chr1:63326576-63327914	chr1	63326576	63327914	8	8	MIR6068	1338	CKD21
chr1:63326396-63327733	chr1	63326396	63327733	6	7	MIR6068	1337	CKD25
chr22:35336654-35336993	chr22	35336654	35336993	0	8	MIR6069	339	CKD4
chr22:35336401-35337067	chr22	35336401	35337067	4	18	MIR6069	666	CKD15
chr22:35336651-35336998	chr22	35336651	35336998	12	13	MIR6069	347	CKD22
chr10:96828561-96828917	chr10	96828561	96828917	0	18	MIR607	356	CKD4
chr10:96828514-96828848	chr10	96828514	96828848	2	11	MIR607	334	CKD8
chr21:43609790-43610142	chr21	43609790	43610142	20	56	MIR6070	352	CKD6
chr21:43609883-43610090	chr21	43609883	43610090	0	6	MIR6070	207	CKD6
chr21:43609222-43610762	chr21	43609222	43610762	2	6	MIR6070	1540	CKD16
chr10:2075962-2076297	chr10	2075962	2076297	3	6	MIR6072	335	CKD4
chr11:15969218-15969912	chr11	15969218	15969912	0	21	MIR6073	694	CKD22
chr12:66023576-66023930	chr12	66023576	66023930	2	7	MIR6074	354	CKD22
chr12:66023469-66024267	chr12	66023469	66024267	48	18	MIR6074	798	HM9
chr5:1510497-1510869	chr5	1510497	1510869	1	11	MIR6075	372	CKD6
chr14:49966335-49966806	chr14	49966335	49966806	34	33	MIR6076	471	CKD9
chr14:49966177-49966534	chr14	49966177	49966534	2	12	MIR6076	357	CKD14
chr14:49966319-49966613	chr14	49966319	49966613	0	9	MIR6076	294	HM6
chr1:148387457-148388535	chr1	148387457	148388535	3	7	MIR6077	1078	CKD25
chr10:3990822-3991545	chr10	3990822	3991545	3	4	MIR6078	723	CKD21
chr1:43838172-43838876	chr1	43838172	43838876	12	58	MIR6079	704	CKD22
chr17:64780308-64780967	chr17	64780308	64780967	15	9	MIR6080	659	CKD3
chr17:64780461-64781074	chr17	64780461	64781074	4	10	MIR6080	613	CKD3
chr17:64780744-64781133	chr17	64780744	64781133	133	529	MIR6080	389	CKD18
chr17:64780505-64780912	chr17	64780505	64780912	14	13	MIR6080	407	CKD22
chr17:64780314-64781012	chr17	64780314	64781012	10	12	MIR6080	698	HM5
chr9:95065251-95065599	chr9	95065251	95065599	152	303	MIR6081	348	CKD20
chr9:95065128-95065968	chr9	95065128	95065968	20	32	MIR6081	840	CKD21
chr9:95065232-95065579	chr9	95065232	95065579	2	12	MIR6081	347	CKD21
chr9:95065103-95065455	chr9	95065103	95065455	15	26	MIR6081	352	CKD22
chr9:95065184-95065550	chr9	95065184	95065550	9	11	MIR6081	366	CKD22
chr9:95065142-95065820	chr9	95065142	95065820	1233	3114	MIR6081	678	HF3
chr4:171185992-171186580	chr4	171185992	171186580	6	14	MIR6082	588	CKD13
chr4:171186075-171186437	chr4	171186075	171186437	6	9	MIR6082	362	CKD21
chr4:171185935-171186307	chr4	171185935	171186307	0	7	MIR6082	372	HM3
chr15:62341701-62343957	chr15	62341701	62343957	5	12	MIR6085	2256	CKD25
chrX:13590013-13591048	chrX	13590013	13591048	3	6	MIR6086	1035	CKD16
chr19:45436447-45436796	chr19	45436447	45436796	7	5	MIR6088	349	CKD8
chr19:45436447-45436796	chr19	45436447	45436796	34	18	MIR6088	349	CKD18
chr19:45436608-45436950	chr19	45436608	45436950	51	79	MIR6088	342	HM11
chr11:61791956-61793280	chr11	61791956	61793280	6	15	MIR611	1324	CKD3
chr11:12163123-12164212	chr11	12163123	12164212	4	19	MIR6124	1089	CKD10
chr16:3485315-3485650	chr16	3485315	3485650	0	8	MIR6126	335	CKD16
chr16:3485217-3485563	chr16	3485217	3485563	5	30	MIR6126	346	CKD18
chr16:3485352-3485731	chr16	3485352	3485731	56	62	MIR6126	379	HM9
chr16:3485217-3485582	chr16	3485217	3485582	3	5	MIR6126	365	HM11
chr1:22632981-22633555	chr1	22632981	22633555	12	14	MIR6127	574	CKD12
chr1:22633011-22633379	chr1	22633011	22633379	4	4	MIR6127	368	CKD25
chr1:22633245-22633633	chr1	22633245	22633633	6	16	MIR6127	388	CKD25
chr1:22632982-22633369	chr1	22632982	22633369	8	18	MIR6127	387	HM1
chr17:49288264-49288660	chr17	49288264	49288660	6	13	MIR6129	396	CKD3
chr12:12764565-12764945	chr12	12764565	12764945	6	31	MIR613	380	CKD4
chr5:10477705-10478451	chr5	10477705	10478451	16	18	MIR6131	746	CKD12
chr5:10478016-10478352	chr5	10478016	10478352	23	72	MIR6131	336	HF6
chr7:117019976-117020336	chr7	117019976	117020336	3	4	MIR6132	360	CKD3
chr7:117020119-117020453	chr7	117020119	117020453	14	54	MIR6132	334	CKD18
chr7:117020187-117020460	chr7	117020187	117020460	5	15	MIR6132	273	CKD22
chrX:28495369-28495718	chrX	28495369	28495718	22	40	MIR6134	349	CKD8
chrX:28495524-28495906	chrX	28495524	28495906	24	44	MIR6134	382	CKD22
chr12:12915529-12916374	chr12	12915529	12916374	3	4	MIR614	845	CKD13
chr12:12915824-12916165	chr12	12915824	12916165	8	24	MIR614	341	HM3
chr12:80832514-80833096	chr12	80832514	80833096	10	10	MIR617	582	CKD22
chr12:80935579-80935860	chr12	80935579	80935860	3	10	MIR618	281	CKD7
chr12:80935284-80935930	chr12	80935284	80935930	6	39	MIR618	646	CKD16
chr12:80935405-80936153	chr12	80935405	80936153	4	18	MIR618	748	CKD22
chr12:108836841-108837503	chr12	108836841	108837503	12	22	MIR619	662	CKD13
chr12:108836650-108837023	chr12	108836650	108837023	92	102	MIR619	373	HM3
chr13:40810217-40810925	chr13	40810217	40810925	9	10	MIR621	708	CKD12
chr13:40810555-40810926	chr13	40810555	40810926	15	42	MIR621	371	CKD12
chr13:40810625-40810995	chr13	40810625	40810995	7	26	MIR621	370	CKD13
chr13:90230944-90231650	chr13	90230944	90231650	4	9	MIR622	706	CKD12
chr13:90231107-90231875	chr13	90231107	90231875	7	22	MIR622	768	HM6

chr13:99356074-99356463	chr13	99356074	99356463	0	7	MIR623	389	CKD3
chr13:99356108-99356329	chr13	99356108	99356329	5	16	MIR623	221	CKD18
chr15:41690702-41692312	chr15	41690702	41692312	8	37	MIR626	1610	CKD16
chr15:70079309-70079648	chr15	70079309	70079648	6	3	MIR629	339	CKD8
chr15:70079331-70079736	chr15	70079331	70079736	34	58	MIR629	405	CKD10
chr15:70079226-70079869	chr15	70079226	70079869	22	29	MIR629	643	CKD25
chr15:70078830-70079799	chr15	70078830	70079799	1346	3068	MIR629	969	HF3
chr15:72586704-72587542	chr15	72586704	72587542	16	12	MIR630	838	CKD3
chr15:75353387-75353775	chr15	75353387	75353775	16	27	MIR631	388	HM5
chr17:62944134-62944483	chr17	62944134	62944483	7	7	MIR633	349	CKD14
chr17:66786898-66787256	chr17	66786898	66787256	0	10	MIR634	358	CKD3
chr17:66786906-66787254	chr17	66786906	66787254	3	6	MIR634	348	CKD4
chr17:66787015-66787546	chr17	66787015	66787546	8	18	MIR634	531	CKD4
chr17:66786924-66788153	chr17	66786924	66788153	7	10	MIR634	1229	CKD12
chr17:66786836-66787181	chr17	66786836	66787181	13	18	MIR634	345	HM19
chr17:68424205-68424584	chr17	68424205	68424584	0	7	MIR635	379	CKD3
chr17:68424293-68424638	chr17	68424293	68424638	8	15	MIR635	345	CKD4
chr17:68424071-68424736	chr17	68424071	68424736	18	64	MIR635	665	CKD13
chr17:68424295-68424639	chr17	68424295	68424639	50	164	MIR635	344	CKD16
chr17:68424413-68425137	chr17	68424413	68425137	58	21	MIR635	724	CKD25
chr19:3961387-3962142	chr19	3961387	3962142	12	23	MIR637	755	CKD3
chr19:3961218-3961547	chr19	3961218	3961547	9	7	MIR637	329	CKD9
chr19:3961189-3961915	chr19	3961189	3961915	39	57	MIR637	726	HM3
chr19:3961279-3961652	chr19	3961279	3961652	15	28	MIR637	373	HM19
chr19:40282259-40283324	chr19	40282259	40283324	15	31	MIR641	1065	HM5
chr19:52281684-52282058	chr19	52281684	52282058	0	4	MIR643	374	CKD12
chr19:52281272-52282054	chr19	52281272	52282054	3	6	MIR643	782	CKD12
chr20:50585716-50586495	chr20	50585716	50586495	124	133	MIR645	779	CKD7
chr20:50584886-50585895	chr20	50584886	50585895	2	7	MIR645	1009	CKD21
chr20:50585688-50586409	chr20	50585688	50586409	0	8	MIR645	721	CKD21
chr20:60308324-60308717	chr20	60308324	60308717	9	23	MIR646	393	CKD4
chr20:60308443-60308815	chr20	60308443	60308815	5	7	MIR646	372	CKD6
chr20:60308325-60308704	chr20	60308325	60308704	2	12	MIR646	379	CKD9
chr20:60308324-60308721	chr20	60308324	60308721	7	6	MIR646	397	CKD25
chr20:60308430-60308747	chr20	60308430	60308747	2	7	MIR646	317	CKD25
chr20:63942542-63942954	chr20	63942542	63942954	6	10	MIR647	412	HM6
chr22:17980653-17981031	chr22	17980653	17981031	13	41	MIR648	378	CKD15
chr22:17980474-17981093	chr22	17980474	17981093	2	15	MIR648	619	CKD20
chr22:17980773-17982236	chr22	17980773	17982236	3	7	MIR648	1463	CKD25
chr22:21033859-21034798	chr22	21033859	21034798	0	7	MIR649	939	CKD21
chr5:151521553-151522359	chr5	151521553	151522359	36	65	MIR6499	806	CKD13
chr5:151521901-151522251	chr5	151521901	151522251	6	7	MIR6499	350	CKD18
chr5:151521693-151522331	chr5	151521693	151522331	0	4	MIR6499	638	CKD21
chr5:151521864-151522786	chr5	151521864	151522786	10	7	MIR6499	922	CKD21
chr22:22821960-22823686	chr22	22821960	22823686	15	10	MIR650	1726	CKD21
chr22:22822659-22823348	chr22	22822659	22823348	12	18	MIR650	689	CKD21
chr1:51059900-51060548	chr1	51059900	51060548	0	5	MIR6500	648	CKD10
chr12:66250888-66251569	chr12	66250888	66251569	16	16	MIR6502	681	CKD3
chr12:66250617-66251203	chr12	66250617	66251203	6	12	MIR6502	586	CKD21
chr12:66250889-66251429	chr12	66250889	66251429	6	11	MIR6502	540	CKD25
chr16:81611098-81611431	chr16	81611098	81611431	5	12	MIR6504	333	CKD16
chr16:81611334-81611542	chr16	81611334	81611542	0	16	MIR6504	208	CKD18
chr12:48132714-48133030	chr12	48132714	48133030	0	4	MIR6505	316	CKD8
chr12:48132714-48133041	chr12	48132714	48133041	0	7	MIR6505	327	CKD8
chr12:48132684-48133388	chr12	48132684	48133388	2	11	MIR6505	704	CKD11
chr12:48132684-48133010	chr12	48132684	48133010	1	6	MIR6505	326	CKD22
chr12:48132789-48133162	chr12	48132789	48133162	13	12	MIR6505	373	HM1
chr21:39447003-39447373	chr21	39447003	39447373	0	5	MIR6508	370	HM5
chr7:135206976-135207406	chr7	135206976	135207406	6	12	MIR6509	430	CKD20
chrX:8126717-8127064	chrX	8126717	8127064	0	8	MIR651	347	CKD15
chr17:41516852-41517630	chr17	41516852	41517630	44	48	MIR6510	778	CKD3
chr17:41516700-41517618	chr17	41516700	41517618	2	6	MIR6510	918	CKD21
chr17:41517075-41517422	chr17	41517075	41517422	0	14	MIR6510	347	CKD22
chr2:177313738-177314123	chr2	177313738	177314123	25	69	MIR6512	385	CKD8
chr2:177313391-177314176	chr2	177313391	177314176	4	22	MIR6512	785	CKD16
chr11:62792252-62792954	chr11	62792252	62792954	4	5	MIR6514	702	CKD14
chr17:77089031-77089584	chr17	77089031	77089584	12	47	MIR6516	553	CKD12
chrX:110055275-110055598	chrX	110055275	110055598	3	19	MIR652	323	CKD10
chr7:93482723-93483093	chr7	93482723	93483093	0	6	MIR653	370	CKD10
chr14:101040041-101040392	chr14	101040041	101040392	61	242	MIR654	351	CKD20
chr14:101039393-101040541	chr14	101039393	101040541	6	13	MIR654	1148	CKD21
chr14:101049310-101049688	chr14	101049310	101049688	8	58	MIR655	378	CKD9
chr14:101049061-101049805	chr14	101049061	101049805	27	22	MIR655	744	CKD12
chr14:101048093-101049658	chr14	101048093	101049658	10	20	MIR655	1565	CKD21
chr14:101049431-101049804	chr14	101049431	101049804	0	6	MIR655	373	CKD21
chr14:101049432-101050553	chr14	101049432	101050553	10	5	MIR655	1121	CKD21
chr14:101049006-101050991	chr14	101049006	101050991	8	9	MIR655	1985	CKD25
chr14:101049329-101049953	chr14	101049329	101049953	28	22	MIR655	624	CKD25

chr14:101049301-101049657	chr14	101049301	101049657	21	23	MIR655	356	HM3
chr14:101066672-101066884	chr14	101066672	101066884	0	54	MIR656	212	CKD10
chr14:101066671-101066884	chr14	101066671	101066884	0	8	MIR656	213	CKD12
chr14:101066711-101067471	chr14	101066711	101067471	1	6	MIR656	760	CKD21
chr14:101066672-101066884	chr14	101066672	101066884	0	13	MIR656	212	CKD22
chr14:101066497-101067136	chr14	101066497	101067136	42	116	MIR656	639	HM1
chr14:101066627-101067187	chr14	101066627	101067187	90	272	MIR656	560	HM5
chr17:81125253-81125637	chr17	81125253	81125637	9	13	MIR657	384	HF4
chr22:37844136-37844692	chr22	37844136	37844692	6	7	MIR658	556	CKD16
chr22:37847222-37847901	chr22	37847222	37847901	12	13	MIR659	679	CKD21
chr22:37847571-37847785	chr22	37847571	37847785	1	11	MIR659	214	HM2
chr20:26208080-26208529	chr20	26208080	26208529	1392	367	MIR663A	449	CKD7
chr2:132256488-132257554	chr2	132256488	132257554	7	18	MIR663B	1066	CKD4
chr1:220200377-220200721	chr1	220200377	220200721	0	8	MIR664A	344	CKD13
chr14:101054987-101055356	chr14	101054987	101055356	8	9	MIR668	369	CKD8
chr14:101054984-101055334	chr14	101054984	101055334	4	10	MIR668	350	CKD12
chr14:101055073-101055418	chr14	101055073	101055418	0	21	MIR668	345	CKD16
chr14:101054995-101055386	chr14	101054995	101055386	8	24	MIR668	391	HF3
chr14:101055122-101055448	chr14	101055122	101055448	48	82	MIR668	326	HM5
chr11:43558945-43561506	chr11	43558945	43561506	22	14	MIR670	2561	CKD13
chr11:43559579-43559779	chr11	43559579	43559779	20	549	MIR670	200	HM15
chr10:112299511-112299881	chr10	112299511	112299881	3	12	MIR6715A	370	CKD3
chr10:112299351-112299893	chr10	112299351	112299893	2	22	MIR6715A	542	CKD13
chr10:112299552-112299952	chr10	112299552	112299952	9	8	MIR6715A	400	CKD14
chr10:112299083-112299821	chr10	112299083	112299821	16	12	MIR6715A	738	CKD25
chr10:112299531-112299882	chr10	112299531	112299882	9	45	MIR6715A	351	HM5
chr10:112299511-112299881	chr10	112299511	112299881	3	12	MIR6715B	370	CKD3
chr10:112299351-112299893	chr10	112299351	112299893	2	22	MIR6715B	542	CKD13
chr10:112299552-112299952	chr10	112299552	112299952	9	8	MIR6715B	400	CKD14
chr10:112299083-112299821	chr10	112299083	112299821	16	12	MIR6715B	738	CKD25
chr10:112299531-112299882	chr10	112299531	112299882	9	45	MIR6715B	351	HM5
chr11:118643806-118644570	chr11	118643806	118644570	20	36	MIR6716	764	CKD9
chr11:118643709-118644104	chr11	118643709	118644104	3	6	MIR6716	395	CKD22
chr14:21022140-21023414	chr14	21022140	21023414	7	12	MIR6717	1274	CKD3
chr14:21023257-21023998	chr14	21023257	21023998	8	16	MIR6717	741	CKD16
chr19:39829626-39829960	chr19	39829626	39829960	4	15	MIR6719	334	CKD6
chr19:39829641-39830011	chr19	39829641	39830011	7	7	MIR6719	370	CKD6
chr19:39829522-39829860	chr19	39829522	39829860	4	6	MIR6719	338	CKD25
chr19:39829516-39829888	chr19	39829516	39829888	38	171	MIR6719	372	HM1
chr9:136746798-136747147	chr9	136746798	136747147	8	30	MIR6722	349	CKD8
chr1:8866404-8866650	chr1	8866404	8866650	16	21	MIR6728	246	CKD16
chr1:8866427-8867096	chr1	8866427	8867096	2	5	MIR6728	669	CKD21
chr1:12028937-12029237	chr1	12028937	12029237	22	150	MIR6729	300	HM5
chr1:12578301-12579087	chr1	12578301	12579087	50	152	MIR6730	786	CKD3
chr1:12578902-12579218	chr1	12578902	12579218	7	134	MIR6730	316	CKD9
chr1:24919117-24919690	chr1	24919117	24919690	58	76	MIR6731	573	HM1
chr1:37480026-37481337	chr1	37480026	37481337	16	20	MIR6732	1311	CKD13
chr1:43171596-43171842	chr1	43171596	43171842	0	15	MIR6733	246	CKD4
chr1:43364578-43364769	chr1	43364578	43364769	0	22	MIR6734	191	CKD7
chr1:43364348-43366255	chr1	43364348	43366255	0	4	MIR6734	1907	CKD21
chr1:43448356-43448735	chr1	43448356	43448735	3	8	MIR6735	379	CKD8
chr1:43448347-43449373	chr1	43448347	43449373	0	9	MIR6735	1026	CKD12
chr1:43448159-43449272	chr1	43448159	43449272	2	9	MIR6735	1113	HM1
chr1:43448454-43448617	chr1	43448454	43448617	0	9	MIR6735	163	HM6
chr1:145850430-145850771	chr1	145850430	145850771	0	4	MIR6736	341	CKD3
chr1:145850389-145850970	chr1	145850389	145850970	14	75	MIR6736	581	CKD4
chr1:153962113-153962457	chr1	153962113	153962457	146	214	MIR6737	344	CKD7
chr1:153962240-153962618	chr1	153962240	153962618	12	28	MIR6737	378	CKD11
chr1:153962214-153962455	chr1	153962214	153962455	2	5	MIR6737	241	CKD14
chr1:201863099-201863472	chr1	201863099	201863472	9	23	MIR6739	373	HM1
chr1:202003108-202003601	chr1	202003108	202003601	42	66	MIR6740	493	CKD21
chr1:225921259-225922461	chr1	225921259	225922461	10	14	MIR6741	1202	CKD12
chr1:228396482-228397227	chr1	228396482	228397227	0	9	MIR6742	745	CKD12
chr11:209281-209581	chr11	209281	209581	17	61	MIR6743	300	CKD20
chr11:62566390-62567113	chr11	62566390	62567113	1	6	MIR6747	723	CKD25
chr11:62789563-62789928	chr11	62789563	62789928	27	45	MIR6748	365	CKD13
chr11:62789246-62790552	chr11	62789246	62790552	3	12	MIR6748	1306	CKD21
chr11:62789743-62790283	chr11	62789743	62790283	1	9	MIR6748	540	HF5
chr11:64898227-64898564	chr11	64898227	64898564	27	62	MIR6750	337	CKD18
chr11:67490175-67490395	chr11	67490175	67490395	2	11	MIR6752	220	CKD4
chr11:67490173-67490566	chr11	67490173	67490566	59	77	MIR6752	393	CKD14
chr11:67490183-67490520	chr11	67490183	67490520	5	13	MIR6752	337	CKD25
chr11:68044654-68044985	chr11	68044654	68044985	11	26	MIR6753	331	CKD18
chr11:68044707-68045081	chr11	68044707	68045081	15	43	MIR6753	374	CKD22
chr11:71473029-71473707	chr11	71473029	71473707	33	17	MIR6754	678	CKD9
chr11:119312891-119313308	chr11	119312891	119313308	12	23	MIR6756	417	CKD4
chr11:119312800-119313139	chr11	119312800	119313139	9	27	MIR6756	339	CKD10
chr12:53056406-53057726	chr12	53056406	53057726	11	14	MIR6757	1320	CKD13

chr12:53056877-53057213	chr12	53056877	53057213	2	13	MIR6757	336	HM1
chr12:57748396-57748973	chr12	57748396	57748973	14	10	MIR6759	577	CKD3
chr12:57748599-57748949	chr12	57748599	57748949	0	10	MIR6759	350	CKD13
chr12:57748187-57748807	chr12	57748187	57748807	10	14	MIR6759	620	HM6
chrX:70022772-70023143	chrX	70022772	70023143	0	6	MIR676	371	CKD4
chrX:70022757-70025956	chrX	70022757	70025956	5	24	MIR676	3199	CKD13
chr12:132581989-132582206	chr12	132581989	132582206	15	50	MIR6763	217	HM7
chr14:100277306-100278123	chr14	100277306	100278123	21	15	MIR6764	817	CKD13
chr14:100277226-100277677	chr14	100277226	100277677	4	5	MIR6764	451	CKD14
chr14:100277051-100277443	chr14	100277051	100277443	4	10	MIR6764	392	HM6
chr14:105150519-105150899	chr14	105150519	105150899	13	19	MIR6765	380	HM6
chr15:89326697-89327207	chr15	89326697	89327207	0	10	MIR6766	510	CKD16
chr16:2445337-2445683	chr16	2445337	2445683	2	12	MIR6767	346	CKD25
chr16:2463380-2464668	chr16	2463380	2464668	2	11	MIR6768	1288	CKD3
chr16:2463679-2464040	chr16	2463679	2464040	8	23	MIR6768	361	CKD8
chr16:2463702-2464062	chr16	2463702	2464062	127	136	MIR6768	360	CKD18
chr16:2463727-2464063	chr16	2463727	2464063	2	10	MIR6768	336	CKD21
chr16:50292435-50292782	chr16	50292435	50292782	12	24	MIR6771	347	CKD6
chr16:50292435-50292782	chr16	50292435	50292782	6	6	MIR6771	347	CKD13
chr16:57772239-57772430	chr16	57772239	57772430	0	6	MIR6772	191	CKD6
chr16:57772239-57772574	chr16	57772239	57772574	2	6	MIR6772	335	HM6
chr16:68233253-68233612	chr16	68233253	68233612	0	6	MIR6773	359	CKD12
chr16:85918241-85918611	chr16	85918241	85918611	12	21	MIR6774	370	CKD3
chr16:85918300-85918649	chr16	85918300	85918649	0	14	MIR6774	349	CKD3
chr16:85918302-85918646	chr16	85918302	85918646	2	4	MIR6774	344	CKD8
chr16:85918285-85918487	chr16	85918285	85918487	0	7	MIR6774	202	CKD20
chr16:85917997-85918561	chr16	85917997	85918561	90	120	MIR6774	564	HM1
chr16:85918302-85918646	chr16	85918302	85918646	4	6	MIR6774	344	HM19
chr16:87834504-87834849	chr16	87834504	87834849	2	10	MIR6775	345	CKD14
chr16:87834468-87834832	chr16	87834468	87834832	28	53	MIR6775	364	CKD24
chr17:17813250-17813914	chr17	17813250	17813914	6	21	MIR6777	664	CKD22
chr17:17813357-17814474	chr17	17813357	17814474	14	13	MIR6777	1117	HF5
chr17:18340738-18341103	chr17	18340738	18341103	45	66	MIR6778	365	HM3
chr17:42707862-42708233	chr17	42707862	42708233	12	18	MIR6780A	371	CKD6
chr6:43434356-43434697	chr6	43434356	43434697	2	5	MIR6780B	341	CKD9
chr6:43434045-43434724	chr6	43434045	43434724	12	45	MIR6780B	679	CKD16
chr17:42823860-42824218	chr17	42823860	42824218	27	46	MIR6781	358	CKD20
chr17:42823869-42824081	chr17	42823869	42824081	0	16	MIR6781	212	CKD22
chr17:42823870-42824082	chr17	42823870	42824082	0	8	MIR6781	212	HM1
chr17:42823662-42824009	chr17	42823662	42824009	9	8	MIR6781	347	HM5
chr17:44934589-44934934	chr17	44934589	44934934	5	54	MIR6783	345	CKD6
chr17:44934418-44934778	chr17	44934418	44934778	4	6	MIR6783	360	CKD13
chr17:44934455-44935016	chr17	44934455	44935016	26	37	MIR6783	561	HF5
chr17:45114337-45114719	chr17	45114337	45114719	5	20	MIR6784	382	CKD20
chr17:45114302-45114626	chr17	45114302	45114626	3	29	MIR6784	324	HM1
chr17:45114303-45114794	chr17	45114303	45114794	75	74	MIR6784	491	HM9
chr17:75497909-75499336	chr17	75497909	75499336	4	6	MIR6785	1427	CKD3
chr17:75498371-75499102	chr17	75498371	75499102	185	199	MIR6785	731	HM11
chr17:82233765-82236755	chr17	82233765	82236755	6	42	MIR6787	2990	CKD13
chr17:82236664-82237030	chr17	82236664	82237030	183	136	MIR6787	366	HF6
chr17:82236664-82237030	chr17	82236664	82237030	12	10	MIR6787	366	HF7
chr18:10759424-10760178	chr18	10759424	10760178	8	17	MIR6788	754	CKD4
chr18:10759475-10759849	chr18	10759475	10759849	22	37	MIR6788	374	CKD6
chr18:10759432-10759803	chr18	10759432	10759803	20	18	MIR6788	371	CKD12
chr18:10759475-10759831	chr18	10759475	10759831	4	6	MIR6788	356	CKD13
chr18:10759426-10759826	chr18	10759426	10759826	4	24	MIR6788	400	CKD16
chr18:10759476-10759889	chr18	10759476	10759889	38	10	MIR6788	413	HM1
chr18:10759532-10759867	chr18	10759532	10759867	9	28	MIR6788	335	HM1
chr18:10759475-10759854	chr18	10759475	10759854	53	118	MIR6788	379	HM19
chr19:6736695-6736931	chr19	6736695	6736931	0	21	MIR6791	236	CKD6
chr19:6736499-6736936	chr19	6736499	6736936	11	37	MIR6791	437	CKD22
chr19:10828922-10829104	chr19	10828922	10829104	1	12	MIR6793	182	CKD14
chr19:10828826-10829172	chr19	10828826	10829172	7	20	MIR6793	346	CKD20
chr19:15179278-15179844	chr19	15179278	15179844	30	14	MIR6795	566	CKD12
chr19:15179092-15179421	chr19	15179092	15179421	9	19	MIR6795	329	CKD25
chr19:15178905-15179418	chr19	15178905	15179418	66	93	MIR6795	513	HM6
chr19:41869272-41870230	chr19	41869272	41870230	24	12	MIR6797	958	CKD12
chr19:49009776-49010089	chr19	49009776	49010089	3	7	MIR6798	313	CKD20
chr19:52221478-52222785	chr19	52221478	52222785	4	13	MIR6801	1307	CKD3
chr19:55239905-55240288	chr19	55239905	55240288	13	16	MIR6802	383	HF4
chr19:55245104-55245463	chr19	55245104	55245463	0	5	MIR6803	359	CKD12
chr19:55245047-55245374	chr19	55245047	55245374	0	5	MIR6803	327	CKD20
chr19:55387926-55388243	chr19	55387926	55388243	17	29	MIR6805	317	CKD9
chr19:58334558-58335248	chr19	58334558	58335248	0	9	MIR6806	690	CKD3
chr19:58334593-58334784	chr19	58334593	58334784	0	6	MIR6806	191	CKD6
chr19:58334403-58334894	chr19	58334403	58334894	25	17	MIR6806	491	CKD9
chr19:58334652-58335323	chr19	58334652	58335323	6	6	MIR6806	671	CKD12
chr19:58334249-58334920	chr19	58334249	58334920	52	28	MIR6806	671	CKD18

chr19:58334650-58334985	chr19	58334650	58334985	105	509	MIR6806	335	CKD20
chr19:58334470-58334791	chr19	58334470	58334791	2	10	MIR6806	321	CKD24
chr2:218341828-218342345	chr2	218341828	218342345	6	7	MIR6810	517	CKD3
chr2:218341902-218342316	chr2	218341902	218342316	14	54	MIR6810	414	CKD22
chr2:237510193-237511422	chr2	237510193	237511422	6	11	MIR6811	1229	CKD3
chr2:237510487-237512045	chr2	237510487	237512045	27	20	MIR6811	1558	CKD14
chr2:237510926-237511590	chr2	237510926	237511590	4	8	MIR6811	664	CKD21
chr20:45425407-45425758	chr20	45425407	45425758	8	31	MIR6812	351	CKD4
chr20:45425451-45425814	chr20	45425451	45425814	117	272	MIR6812	363	CKD20
chr20:45425452-45425813	chr20	45425452	45425813	4	24	MIR6812	361	CKD21
chr20:45425452-45425678	chr20	45425452	45425678	6	49	MIR6812	226	CKD22
chr20:45425360-45425697	chr20	45425360	45425697	12	5	MIR6812	337	HM19
chr20:64076665-64077022	chr20	64076665	64077022	0	7	MIR6813	357	CKD4
chr20:64076895-64077209	chr20	64076895	64077209	0	7	MIR6813	314	CKD4
chr21:41746300-41746985	chr21	41746300	41746985	2	7	MIR6814	685	CKD3
chr21:41746326-41747005	chr21	41746326	41747005	6	6	MIR6814	679	HM11
chr22:20114511-20115326	chr22	20114511	20115326	0	6	MIR6816	815	CKD25
chr22:20114236-20115097	chr22	20114236	20115097	5	9	MIR6816	861	HM9
chr22:25454844-25455935	chr22	25454844	25455935	96	82	MIR6817	1091	CKD8
chr22:25454438-25455934	chr22	25454438	25455934	40	18	MIR6817	1496	HM1
chr22:36286381-36287040	chr22	36286381	36287040	15	10	MIR6819	659	CKD4
chr22:37967193-37968149	chr22	37967193	37968149	9	34	MIR6820	956	HF4
chr22:49962655-49962980	chr22	49962655	49962980	4	23	MIR6821	325	CKD6
chr22:49962648-49962978	chr22	49962648	49962978	21	24	MIR6821	330	CKD18
chr3:39138167-39138335	chr3	39138167	39138335	0	17	MIR6822	168	CKD14
chr3:39137905-39138295	chr3	39137905	39138295	2	5	MIR6822	390	HF5
chr3:48549787-48550147	chr3	48549787	48550147	0	6	MIR6823	360	CKD13
chr3:48633203-48634192	chr3	48633203	48634192	11	39	MIR6824	989	CKD13
chr3:129272050-129272421	chr3	129272050	129272421	15	23	MIR6826	371	HM1
chr3:134367750-134367932	chr3	134367750	134367932	0	12	MIR6827	182	CKD14
chr3:134367626-134367975	chr3	134367626	134367975	0	67	MIR6827	349	CKD22
chr3:170422823-170423221	chr3	170422823	170423221	6	36	MIR6828	398	HM19
chr3:195882091-195882419	chr3	195882091	195882419	0	28	MIR6829	328	CKD13
chr3:195882153-195882478	chr3	195882153	195882478	21	93	MIR6829	325	HM1
chr5:132217803-132218450	chr5	132217803	132218450	1	13	MIR6830	647	CKD8
chr5:140563335-140563795	chr5	140563335	140563795	2	6	MIR6831	460	CKD3
chr7:2256227-2257831	chr7	2256227	2257831	6	24	MIR6836	1604	CKD25
chr7:44051564-44051901	chr7	44051564	44051901	19	41	MIR6837	337	HM3
chr7:44073320-44073683	chr7	44073320	44073683	6	5	MIR6838	363	CKD21
chr7:44072847-44073981	chr7	44072847	44073981	34	41	MIR6838	1134	HM1
chr7:64678880-64679241	chr7	64678880	64679241	0	15	MIR6839	361	CKD14
chr7:100356612-100356949	chr7	100356612	100356949	4	12	MIR6840	337	HM6
chr8:24953436-24954018	chr8	24953436	24954018	4	17	MIR6841	582	CKD3
chr8:27433290-27434164	chr8	27433290	27434164	6	10	MIR6842	874	CKD3
chr8:27433264-27433624	chr8	27433264	27433624	6	10	MIR6842	360	CKD8
chr8:27433111-27433437	chr8	27433111	27433437	4	4	MIR6842	326	CKD14
chr8:27433167-27433494	chr8	27433167	27433494	2	6	MIR6842	327	CKD14
chr8:27432353-27433768	chr8	27432353	27433768	15	15	MIR6842	1415	CKD21
chr8:27433119-27433800	chr8	27433119	27433800	129	259	MIR6842	681	HM6
chr8:27610453-27610838	chr8	27610453	27610838	5	28	MIR6843	385	CKD13
chr8:143837726-143838063	chr8	143837726	143838063	0	8	MIR6845	337	CKD4
chr8:144056724-144057460	chr8	144056724	144057460	2	7	MIR6846	736	CKD11
chr8:144057062-144057610	chr8	144057062	144057610	62	39	MIR6846	548	HM13
chr8:144079146-144080195	chr8	144079146	144080195	2	4	MIR6847	1049	CKD3
chr8:144079730-144080057	chr8	144079730	144080057	14	20	MIR6847	327	CKD24
chr8:144317232-144317602	chr8	144317232	144317602	52	68	MIR6848	370	CKD8
chr8:144317158-144317560	chr8	144317158	144317560	2	14	MIR6848	402	CKD18
chr8:144317157-144317529	chr8	144317157	144317529	24	67	MIR6848	372	CKD20
chr8:144400163-144400490	chr8	144400163	144400490	35	100	MIR6849	327	CKD9
chr8:144400066-144400410	chr8	144400066	144400410	100	91	MIR6849	344	CKD24
chr8:144400082-144400439	chr8	144400082	144400439	4	18	MIR6849	357	HM1
chr9:35710495-35710871	chr9	35710495	35710871	0	4	MIR6852	376	CKD12
chr9:35710584-35710808	chr9	35710584	35710808	0	16	MIR6852	224	HF3
chr9:35732887-35733244	chr9	35732887	35733244	0	8	MIR6853	357	CKD3
chr9:35732635-35733308	chr9	35732635	35733308	14	14	MIR6853	673	CKD12
chr9:35732686-35733240	chr9	35732686	35733240	61	42	MIR6853	554	HF4
chr9:35732692-35733283	chr9	35732692	35733283	48	93	MIR6853	591	HM1
chr9:98228924-98229294	chr9	98228924	98229294	1	6	MIR6854	370	CKD6
chr9:98228872-98229789	chr9	98228872	98229789	8	7	MIR6854	917	CKD9
chr9:98228775-98229502	chr9	98228775	98229502	9	45	MIR6854	727	CKD16
chr9:98228846-98229927	chr9	98228846	98229927	20	40	MIR6854	1081	CKD16
chr9:98229133-98229333	chr9	98229133	98229333	0	12	MIR6854	200	CKD20
chr9:129869478-129869835	chr9	129869478	129869835	6	8	MIR6855	357	CKD12
chr9:129869163-129870262	chr9	129869163	129870262	30	50	MIR6855	1099	CKD16
chr9:129869249-129869964	chr9	129869249	129869964	110	305	MIR6855	715	CKD18
chrX:53405442-53405795	chrX	53405442	53405795	4	11	MIR6857	353	CKD8
chrX:53405608-53406364	chrX	53405608	53406364	3	4	MIR6857	756	CKD21
chrX:53405635-53405993	chrX	53405635	53405993	113	255	MIR6857	358	CKD24

chrX:154450282-154450653	chrX	154450282	154450653	4	4	MIR6858	371	CKD4
chrX:154450286-154450634	chrX	154450286	154450634	9	29	MIR6858	348	CKD6
chrX:154450076-154450444	chrX	154450076	154450444	30	32	MIR6858	368	CKD14
chrX:154450276-154450447	chrX	154450276	154450447	8	178	MIR6858	171	HF7
chr16:56904186-56904583	chr16	56904186	56904583	18	18	MIR6863	397	HF4
chr17:4969623-4970220	chr17	4969623	4970220	9	3	MIR6864	597	CKD16
chr17:4969537-4969918	chr17	4969537	4969918	9	20	MIR6864	381	HF5
chr17:4969277-4970119	chr17	4969277	4970119	10	7	MIR6864	842	HM19
chr17:4969623-4970220	chr17	4969623	4970220	9	3	MIR6865	597	CKD16
chr17:40161777-40162129	chr17	40161777	40162129	4	3	MIR6866	352	CKD13
chr17:40193331-40193679	chr17	40193331	40193679	0	10	MIR6867	348	CKD12
chr17:76097961-76098328	chr17	76097961	76098328	13	17	MIR6868	367	CKD14
chr17:76097790-76098162	chr17	76097790	76098162	6	8	MIR6868	372	HM9
chr20:10648077-10650382	chr20	10648077	10650382	1	11	MIR6870	2305	CKD3
chr20:41168732-41169392	chr20	41168732	41169392	12	73	MIR6871	660	HF5
chr20:41168750-41169089	chr20	41168750	41169089	42	150	MIR6871	339	HM19
chr3:50273157-50273473	chr3	50273157	50273473	3	12	MIR6872	316	CKD14
chr7:5711579-5711926	chr7	5711579	5711926	6	6	MIR6874	347	CKD14
chr7:100867485-100868211	chr7	100867485	100868211	30	72	MIR6875	726	CKD9
chr8:25345344-25346214	chr8	25345344	25346214	15	14	MIR6876	870	CKD21
chr1:150492271-150492473	chr1	150492271	150492473	24	159	MIR6878	202	CKD9
chr1:150492016-150492410	chr1	150492016	150492410	6	21	MIR6878	394	CKD21
chr11:65018234-65018600	chr11	65018234	65018600	2	11	MIR6879	366	CKD4
chr11:65018301-65019317	chr11	65018301	65019317	30	36	MIR6879	1016	CKD8
chr11:65018234-65019809	chr11	65018234	65019809	6	30	MIR6879	1575	CKD16
chr11:65018444-65019535	chr11	65018444	65019535	78	4	MIR6879	1091	CKD21
chr11:65017949-65018610	chr11	65017949	65018610	26	99	MIR6879	661	HM1
chr11:65018467-65018801	chr11	65018467	65018801	366	473	MIR6879	334	HM16
chr12:124337094-124337477	chr12	124337094	124337477	7	18	MIR6880	383	CKD3
chr12:124337051-124337422	chr12	124337051	124337422	2	10	MIR6880	371	HM1
chr12:124337096-124337448	chr12	124337096	124337448	39	94	MIR6880	352	HM6
chr15:74411324-74411669	chr15	74411324	74411669	0	10	MIR6881	345	CKD3
chr15:74411115-74411477	chr15	74411115	74411477	13	58	MIR6881	362	CKD20
chr15:74411209-74411556	chr15	74411209	74411556	20	49	MIR6881	347	HM11
chr17:8144795-8145464	chr17	8144795	8145464	4	5	MIR6883	669	CKD21
chr17:40025858-40027165	chr17	40025858	40027165	138	178	MIR6884	1307	CKD10
chr19:6388973-6389807	chr19	6388973	6389807	116	173	MIR6885	834	HM11
chr19:35122466-35122996	chr19	35122466	35122996	14	21	MIR6887	530	CKD16
chr19:35121597-35123140	chr19	35121597	35123140	2	22	MIR6887	1543	CKD25
chr2:159186092-159187243	chr2	159186092	159187243	16	7	MIR6888	1151	CKD3
chr22:41252976-41253313	chr22	41252976	41253313	2	13	MIR6889	337	CKD6
chr7:143382678-143382902	chr7	143382678	143382902	5	182	MIR6892	224	CKD24
chr8:144434986-144435674	chr8	144434986	144435674	2	4	MIR6893	688	CKD22
chrX:53198464-53199165	chrX	53198464	53199165	28	53	MIR6894	701	CKD9
chrX:53198607-53199145	chrX	53198607	53199145	44	68	MIR6894	538	CKD16
chrX:53195171-53195519	chrX	53195171	53195519	4	8	MIR6895	348	CKD22
chr9:83969624-83970525	chr9	83969624	83970525	24	12	MIR7-1	901	CKD3
chr9:83969711-83970050	chr9	83969711	83970050	0	10	MIR7-1	339	CKD3
chr9:83969718-83970092	chr9	83969718	83970092	18	20	MIR7-1	374	CKD6
chr9:83969399-83970400	chr9	83969399	83970400	0	6	MIR7-1	1001	CKD25
chr12:113159028-113159431	chr12	113159028	113159431	44	87	MIR7106	403	HM1
chr12:121443344-121444581	chr12	121443344	121444581	4	6	MIR7107	1237	CKD3
chr12:121444038-121444582	chr12	121444038	121444582	6	14	MIR7107	544	CKD6
chr12:121444105-121447351	chr12	121444105	121447351	9	15	MIR7107	3246	CKD13
chr12:121444079-121444472	chr12	121444079	121444472	465	476	MIR7107	393	HF8
chr22:31621466-31621794	chr22	31621466	31621794	27	59	MIR7109	328	HF6
chr22:31621457-31621792	chr22	31621457	31621792	100	31	MIR7109	335	HM19
chr3:48578819-48579504	chr3	48578819	48579504	30	14	MIR711	685	CKD3
chr3:48578795-48579507	chr3	48578795	48579507	72	88	MIR711	712	CKD8
chr3:48578648-48579012	chr3	48578648	48579012	4	28	MIR711	364	CKD21
chr3:48578760-48579115	chr3	48578760	48579115	85	78	MIR711	355	HF5
chr3:48578650-48579028	chr3	48578650	48579028	446	412	MIR711	378	HM19
chr3:123161752-123162133	chr3	123161752	123162133	8	20	MIR7110	381	CKD16
chr3:123161784-123162099	chr3	123161784	123162099	3	13	MIR7110	315	CKD24
chr6:35470413-35471308	chr6	35470413	35471308	10	11	MIR7111	895	HM19
chr9:123485489-123485741	chr9	123485489	123485741	0	5	MIR7150	252	CKD10
chr9:123485494-123485681	chr9	123485494	123485681	0	18	MIR7150	187	CKD24
chr9:123485494-123485700	chr9	123485494	123485700	3	13	MIR7150	206	CKD24
chr9:123485356-123485741	chr9	123485356	123485741	0	14	MIR7150	385	HM1
chr9:123485520-123485816	chr9	123485520	123485816	2	16	MIR7150	296	HM5
chr10:67403240-67403990	chr10	67403240	67403990	14	18	MIR7151	750	CKD25
chr10:67403024-67403421	chr10	67403024	67403421	31	36	MIR7151	397	HM3
chr10:67403292-67403651	chr10	67403292	67403651	2	11	MIR7151	359	HM5
chr10:67403290-67403679	chr10	67403290	67403679	13	9	MIR7151	389	HM11
chr18:11654712-11655108	chr18	11654712	11655108	36	52	MIR7153	396	CKD6
chr11:45690657-45692730	chr11	45690657	45692730	4	21	MIR7154	2073	CKD16
chr11:64341296-64341957	chr11	64341296	64341957	3	30	MIR7155	661	CKD3
chr1:77060080-77060425	chr1	77060080	77060425	8	93	MIR7156	345	CKD13

chr1:77059989-77060530	chr1	77059989	77060530	28	47	MIR7156	541	HM1
chr1:77060073-77060447	chr1	77060073	77060447	16	86	MIR7156	374	HM5
chr2:140586353-140586692	chr2	140586353	140586692	2	5	MIR7157	339	HM13
chr2:5974573-5974968	chr2	5974573	5974968	106	65	MIR7158	395	CKD6
chr2:5974650-5974840	chr2	5974650	5974840	1	5	MIR7158	190	CKD18
chr6:33898844-33899398	chr6	33898844	33899398	6	6	MIR7159	554	CKD3
chr6:33898967-33899327	chr6	33898967	33899327	3	5	MIR7159	360	CKD20
chr6:158609480-158610642	chr6	158609480	158610642	9	16	MIR7161	1162	CKD16
chr15:88611746-88612079	chr15	88611746	88612079	4	8	MIR7-2	333	CKD6
chr17:12081727-12082284	chr17	12081727	12082284	20	26	MIR744	557	CKD3
chr17:12081384-12082135	chr17	12081384	12082135	9	10	MIR744	751	CKD12
chr17:12081811-12082448	chr17	12081811	12082448	12	64	MIR744	637	CKD16
chr17:12081671-12082423	chr17	12081671	12082423	6	29	MIR744	752	HM5
chr2:6650140-6650481	chr2	6650140	6650481	4	20	MIR7515	341	CKD4
chr2:6649787-6650893	chr2	6649787	6650893	6	10	MIR7515	1106	CKD21
chr14:101025784-101026521	chr14	101025784	101026521	10	18	MIR758	737	CKD12
chr14:101025810-101026204	chr14	101025810	101026204	5	18	MIR758	394	CKD12
chr13:52809907-52810278	chr13	52809907	52810278	12	15	MIR759	371	CKD7
chr1:93846483-93847219	chr1	93846483	93847219	12	28	MIR760	736	CKD6
chr1:51835797-51836695	chr1	51835797	51836695	0	15	MIR761	898	HM1
chr1:156936019-156936355	chr1	156936019	156936355	2	12	MIR765	336	CKD12
chr1:156936004-156936329	chr1	156936004	156936329	14	32	MIR765	325	HM1
chr9:111271081-111271265	chr9	111271081	111271265	0	4	MIR7702	184	CKD14
chr2:176188687-176189067	chr2	176188687	176189067	3	3	MIR7704	380	CKD13
chr8:100702428-100703120	chr8	100702428	100703120	6	9	MIR7705	692	CKD21
chr8:100701901-100704331	chr8	100701901	100704331	3	6	MIR7705	2430	CKD25
chr14:72516648-72517038	chr14	72516648	72517038	3	19	MIR7843	390	CKD8
chr14:72516618-72517011	chr14	72516618	72517011	6	9	MIR7843	393	CKD13
chr14:72515995-72516984	chr14	72515995	72516984	58	89	MIR7843	989	CKD22
chr14:72516649-72516991	chr14	72516649	72516991	10	31	MIR7843	342	CKD22
chr12:94570965-94571355	chr12	94570965	94571355	0	28	MIR7844	390	CKD6
chr2:207166290-207166639	chr2	207166290	207166639	0	11	MIR7845	349	CKD16
chr11:1879439-1880193	chr11	1879439	1880193	8	44	MIR7847	754	CKD3
chr11:1879768-1880631	chr11	1879768	1880631	66	12	MIR7847	863	CKD8
chr11:1879863-1880456	chr11	1879863	1880456	21	6	MIR7847	593	CKD8
chr11:1879952-1880311	chr11	1879952	1880311	8	27	MIR7847	359	CKD18
chr8:133046279-133046623	chr8	133046279	133046623	25	53	MIR7848	344	CKD6
chr19:2630602-2630960	chr19	2630602	2630960	4	14	MIR7850	358	CKD20
chr12:42323597-42323975	chr12	42323597	42323975	0	26	MIR7851	378	CKD8
chr12:42323690-42324434	chr12	42323690	42324434	4	8	MIR7851	744	CKD21
chr12:42323660-42324720	chr12	42323660	42324720	3	13	MIR7851	1060	CKD25
chr1:107896919-107899660	chr1	107896919	107899660	12	42	MIR7852	2741	CKD16
chr1:107896964-107897329	chr1	107896964	107897329	2	5	MIR7852	365	CKD24
chr6:6168694-6169969	chr6	6168694	6169969	9	24	MIR7853	1275	CKD3
chr6:6169268-6169436	chr6	6169268	6169436	0	4	MIR7853	168	CKD6
chr6:6169080-6169438	chr6	6169080	6169438	0	7	MIR7853	358	HM6
chr16:81533808-81534170	chr16	81533808	81534170	6	4	MIR7854	362	CKD12
chr16:81533765-81534105	chr16	81533765	81534105	12	18	MIR7854	340	CKD22
chr16:81533463-81534078	chr16	81533463	81534078	24	49	MIR7854	615	HM5
chr14:64785477-64785835	chr14	64785477	64785835	0	5	MIR7855	358	HM1
chr1:86357506-86358256	chr1	86357506	86358256	2	6	MIR7856	750	CKD16
chr19:55123177-55123375	chr19	55123177	55123375	0	6	MIR7975	198	CKD13
chr19:55123033-55123402	chr19	55123033	55123402	7	17	MIR7975	369	HM7
chr3:176514980-176515346	chr3	176514980	176515346	2	4	MIR7977	366	CKD14
chr3:176514860-176515207	chr3	176514860	176515207	11	8	MIR7977	347	CKD24
chr21:35720508-35721618	chr21	35720508	35721618	0	8	MIR802	1110	CKD3
chr21:35720674-35721198	chr21	35720674	35721198	4	9	MIR802	524	CKD16
chr11:130666625-130666939	chr11	130666625	130666939	8	22	MIR8052	314	CKD9
chr11:130666410-130666939	chr11	130666410	130666939	57	100	MIR8052	529	HM1
chr11:23418917-23419279	chr11	23418917	23419279	9	15	MIR8054	362	CKD6
chr8:6621846-6622222	chr8	6621846	6622222	5	11	MIR8055	376	HM6
chr5:173347323-173347670	chr5	173347323	173347670	0	4	MIR8056	347	HM1
chr18:26591363-26591712	chr18	26591363	26591712	2	45	MIR8057	349	CKD18
chr17:50768365-50768939	chr17	50768365	50768939	33	49	MIR8059	574	CKD18
chr17:50768569-50768939	chr17	50768569	50768939	6	18	MIR8059	370	CKD22
chr17:50768336-50768734	chr17	50768336	50768734	2	5	MIR8059	398	CKD25
chr17:50768445-50769138	chr17	50768445	50769138	315	631	MIR8059	693	HF3
chr17:50768618-50768819	chr17	50768618	50768819	1	18	MIR8059	201	HM3
chr20:7371451-7371991	chr20	7371451	7371991	6	10	MIR8062	540	CKD8
chr20:7371380-7371735	chr20	7371380	7371735	2	10	MIR8062	355	CKD25
chr15:36972250-36973389	chr15	36972250	36973389	36	85	MIR8063	1139	CKD10
chr3:52846439-52846660	chr3	52846439	52846660	0	20	MIR8064	221	CKD3
chr3:52846439-52847465	chr3	52846439	52847465	6	6	MIR8064	1026	CKD21
chr3:52846448-52846824	chr3	52846448	52846824	6	8	MIR8064	376	CKD21
chr3:52846242-52846605	chr3	52846242	52846605	0	6	MIR8064	363	HF4
chr3:52846242-52846605	chr3	52846242	52846605	7	14	MIR8064	363	HM19
chr16:5632377-5632726	chr16	5632377	5632726	7	16	MIR8065	349	CKD7
chr16:5632448-5632804	chr16	5632448	5632804	0	7	MIR8065	356	CKD21

chr16:5632310-5632693	chr16	5632310	5632693	6	16	MIR8065	383	CKD25
chr4:101240620-101241350	chr4	101240620	101241350	6	41	MIR8066	730	CKD13
chr15:62304425-62304813	chr15	62304425	62304813	2	6	MIR8067	388	CKD3
chr15:62304515-62304880	chr15	62304515	62304880	2	14	MIR8067	365	CKD12
chr15:62304593-62305221	chr15	62304593	62305221	12	13	MIR8067	628	CKD22
chr11:28477142-28478973	chr11	28477142	28478973	10	10	MIR8068	1831	CKD3
chr11:11783053-11783837	chr11	11783053	11783837	6	3	MIR8070	784	CKD8
chr13:110340876-110341546	chr13	110340876	110341546	8	10	MIR8073	670	CKD8
chr13:110340844-110341159	chr13	110340844	110341159	2	10	MIR8073	315	HM19
chr19:51206051-51207320	chr19	51206051	51207320	9	6	MIR8074	1269	CKD25
chr13:113262487-113263250	chr13	113262487	113263250	0	4	MIR8075	763	CKD21
chr3:113431559-113432300	chr3	113431559	113432300	6	8	MIR8076	741	CKD8
chr3:113431889-113432261	chr3	113431889	113432261	4	6	MIR8076	372	CKD21
chr3:113431889-113432262	chr3	113431889	113432262	8	10	MIR8076	373	HM1
chr19:42351112-42351465	chr19	42351112	42351465	0	7	MIR8077	353	CKD25
chr13:44195990-44196351	chr13	44195990	44196351	9	38	MIR8079	361	CKD4
chr13:44195721-44196391	chr13	44195721	44196391	0	8	MIR8079	670	CKD12
chr2:79866456-79866783	chr2	79866456	79866783	0	8	MIR8080	327	CKD6
chr2:79866336-79866704	chr2	79866336	79866704	12	18	MIR8080	368	HF4
chr2:79866446-79866868	chr2	79866446	79866868	28	45	MIR8080	422	HM3
chr9:106600804-106601670	chr9	106600804	106601670	9	10	MIR8081	866	CKD3
chr4:113152240-113152380	chr4	113152240	113152380	0	6	MIR8082	140	CKD25
chr10:28288927-28289876	chr10	28288927	28289876	12	11	MIR8086	949	CKD3
chr10:28288764-28289371	chr10	28288764	28289371	16	21	MIR8086	607	CKD8
chr5:181043264-181043587	chr5	181043264	181043587	0	3	MIR8089	323	CKD8
chr2:50695900-50696280	chr2	50695900	50696280	52	75	MIR8485	380	HM7
chr9:28888422-28889192	chr9	28888422	28889192	6	14	MIR873	770	CKD12
chr9:28888829-28889078	chr9	28888829	28889078	1	7	MIR873	249	HM19
chr5:137647257-137648047	chr5	137647257	137648047	2	4	MIR874	790	CKD3
chr5:137647481-137647819	chr5	137647481	137647819	4	11	MIR874	338	CKD12
chr8:99536657-99537006	chr8	99536657	99537006	0	8	MIR875	349	CKD12
chr9:28863586-28863778	chr9	28863586	28863778	0	7	MIR876	192	CKD18
chr9:28863539-28864121	chr9	28863539	28864121	0	12	MIR876	582	CKD21
chr3:10394115-10394801	chr3	10394115	10394801	18	19	MIR885	686	CKD14
chr3:10394181-10394791	chr3	10394181	10394791	14	14	MIR885	610	CKD22
chrX:145997095-145997287	chrX	145997095	145997287	12	42	MIR892B	192	CKD21
chr1:156419977-156420535	chr1	156419977	156420535	8	16	MIR9-1	558	CKD16
chr1:156420076-156420742	chr1	156420076	156420742	104	255	MIR9-1	666	CKD18
chr5:88666468-88667684	chr5	88666468	88667684	5	16	MIR9-2	1216	CKD16
chr5:88666321-88666983	chr5	88666321	88666983	4	20	MIR9-2	662	HF1
chr1:166154527-166155330	chr1	166154527	166155330	4	10	MIR921	803	CKD3
chr1:166154524-166154894	chr1	166154524	166154894	6	10	MIR921	370	CKD6
chr1:166153794-166154862	chr1	166153794	166154862	18	30	MIR921	1068	CKD9
chr1:166154576-166154947	chr1	166154576	166154947	21	42	MIR921	371	CKD10
chr3:197674370-197674761	chr3	197674370	197674761	9	16	MIR922	391	CKD6
chr3:197674404-197674757	chr3	197674404	197674757	0	3	MIR922	353	CKD21
chr3:197674401-197674760	chr3	197674401	197674760	20	23	MIR922	359	HM19
chr18:39622103-39622466	chr18	39622103	39622466	6	25	MIR924	363	CKD6
chr18:39622067-39622288	chr18	39622067	39622288	2	8	MIR924	221	HM3
chr18:39621388-39622188	chr18	39621388	39622188	32	53	MIR924	800	HM11
chrX:134169391-134169749	chrX	134169391	134169749	21	175	MIR92A2	358	CKD3
chrX:134168011-134169914	chrX	134168011	134169914	9	5	MIR92A2	1903	CKD13
chrX:134167220-134170034	chrX	134167220	134170034	0	14	MIR92A2	2814	CKD16
chrX:134169464-134169820	chrX	134169464	134169820	7	13	MIR92A2	356	CKD17
chr2:175167465-175167752	chr2	175167465	175167752	16	312	MIR933	287	CKD6
chr2:175167569-175167817	chr2	175167569	175167817	6	12	MIR933	248	CKD12
chr2:175167415-175167734	chr2	175167415	175167734	5	10	MIR933	319	CKD14
chrX:136550598-136550989	chrX	136550598	136550989	8	10	MIR934	391	CKD14
chr10:104047908-104048265	chr10	104047908	104048265	14	37	MIR936	357	CKD6
chr8:144393696-144394702	chr8	144393696	144394702	12	45	MIR939	1006	CKD16
chr8:144393559-144394804	chr8	144393559	144394804	12	23	MIR939	1245	CKD25
chr8:144393690-144394658	chr8	144393690	144394658	3	9	MIR939	968	CKD25
chr8:144393983-144394347	chr8	144393983	144394347	7	9	MIR939	364	HM3
chr1:117094329-117094881	chr1	117094329	117094881	0	3	MIR942	552	CKD25
chr3:189829834-189830238	chr3	189829834	189830238	11	7	MIR944	404	CKD9
chr3:189829384-189830698	chr3	189829384	189830698	2	9	MIR944	1314	CKD25
chr4:8005082-8005440	chr4	8005082	8005440	22	50	MIR95	358	CKD3
chr4:8004444-8005439	chr4	8004444	8005439	22	47	MIR95	995	CKD9
chr4:8004362-8005676	chr4	8004362	8005676	3	10	MIR95	1314	CKD12
chr4:8005160-8005407	chr4	8005160	8005407	0	25	MIR95	247	HM6
chr2:218822892-218823202	chr2	218822892	218823202	2	11	MIR9500	310	CKD16
chr7:129774122-129774816	chr7	129774122	129774816	38	30	MIR96	694	CKD3
chr7:129773938-129774891	chr7	129773938	129774891	30	32	MIR96	953	CKD4
chr7:129774458-129774791	chr7	129774458	129774791	1	67	MIR96	333	CKD20
chr21:16539074-16539388	chr21	16539074	16539388	0	4	MIR99A	314	HF1
chr19:51692398-51692744	chr19	51692398	51692744	0	6	MIR99B	346	HM1
chr9:94175821-94176156	chr9	94175821	94176156	0	3	MIRLET7A1	335	CKD12
chr9:94175897-94176108	chr9	94175897	94176108	0	6	MIRLET7A1	211	CKD16

chr11:122145923-122146871	chr11	122145923	122146871	2	21	MIRLET7A2	948	CKD3
chr11:122145910-122146847	chr11	122145910	122146847	0	7	MIRLET7A2	937	CKD16
chr22:46112495-46112866	chr22	46112495	46112866	10	15	MIRLET7A3	371	CKD4
chr22:46112385-46113205	chr22	46112385	46113205	67	144	MIRLET7A3	820	CKD18
chr22:46112712-46113035	chr22	46112712	46113035	2	8	MIRLET7A3	323	CKD22
chr22:46112719-46113089	chr22	46112719	46113089	1	22	MIRLET7A3	370	HF5
chr22:46112239-46112962	chr22	46112239	46112962	32	44	MIRLET7A3	723	HM1
chr22:46113471-46113854	chr22	46113471	46113854	2	12	MIRLET7B	383	CKD13
chr22:46113465-46113793	chr22	46113465	46113793	5	23	MIRLET7B	328	CKD20
chr21:16539580-16540433	chr21	16539580	16540433	4	5	MIRLET7C	853	CKD21
chr9:94178791-94179117	chr9	94178791	94179117	18	6	MIRLET7D	326	CKD9
chr9:94176035-94176960	chr9	94176035	94176960	2	24	MIRLET7F1	925	CKD9
chrX:53557108-53557524	chrX	53557108	53557524	2	6	MIRLET7F2	416	CKD13
chrX:53557189-53557401	chrX	53557189	53557401	2	4	MIRLET7F2	212	CKD13
chr12:62603537-62604069	chr12	62603537	62604069	10	4	MIRLET7I	532	CKD12
chr1:5563865-5564242	chr1	5563865	5564242	10	12	Z97988.1	377	CKD4
chr1:5563984-5566288	chr1	5563984	5566288	0	5	Z97988.1	2304	CKD21

Table S4. Description of MIR3200-eccDNAs validated in Fig 7f, g.

eccDNA	Chromosome	Start	End	Discordant	Splits	sample	MiRNA	eccDNA size (bp)	Forward primer	Reverse primer	PCR product length (bp)
C1	chr22	30731133	30731822	52	67	CKD3	MIR3200	689	CGAGAGAGACAAAGAAGGAGGTGGT	ACCTCCCAAAGTGTCAGCTCTTTAT	375
C2	chr22	30731413	30731757	12	86	CKD6	MIR3200	344	CATCGAGAGAGACAAAGAAGGAGGT	AATGCCACCGTGGAAAATAGGATAT	297
C3	chr22	30731414	30731947	16	19	CKD12	MIR3200	533	GTGACCTGGATGAGGCATCAT	AGGATATGAAAAGAACAGGTCAGAT	486
C4	chr22	30731413	30731740	10	26	CKD13	MIR3200	327	GCATTTCATACAAGCCCAGAAGT	TGAAAAGAACAGGTCAGATGAATCT	308
C5	chr22	30731147	30731756	32	58	CKD16	MIR3200	609	TGGCACATGGACTTTTCCAGAACTT	GAGCTGGGTGGTCACTTTGATCTCT	538
C6	chr22	30731336	30731703	78	87	CKD20	MIR3200	367	AAGGAGGTGGTCGAGGGAAT	AGATGAATCTCCCCCCACTCTT	264
C7	chr22	30731485	30731856	0	5	CKD21	MIR3200	371	TACTTCAATGTGGGAATCTCCGTTT	TGTGGACTGTATTGGACACAAACCT	322
C8	chr22	30731414	30731969	1	5	CKD22	MIR3200	555	TTCCTTCCCTGAGCAGGAAGGT	GTAGCGCAAGGTGTGGACTGTATT	467
